# Association of environmental pollutants with asthma and allergy, and the mediating role of oxidative stress and immune markers in adolescents

**DOI:** 10.1016/j.envres.2024.120445

**Published:** 2025-01-15

**Authors:** Hamid Y. Hassen, Eva Govarts, Sylvie Remy, Bianca Cox, Nina Iszatt, Lützen Portengen, Adrian Covaci, Greet Schoeters, Elly Den Hond, Stefaan De Henauw, Liesbeth Bruckers, Gudrun Koppen, Veerle J. Verheyen

**Affiliations:** a Environmental Intelligence Unit, Flemish Institute for Technological Research (VITO), Boeretang 200, 2400, Mol, Belgium; b Division of Climate and Environmental Health, Norwegian Institute of Public Health, Oslo, Norway; c Centre for Sustainable Diets, Norwegian Institute of Public Health, Oslo, Norway; d Institute for Risk Assessment Sciences, Utrecht University, Utrecht, the Netherlands; e Toxicological Center, University of Antwerp, Universiteitsplein 1, 2610, Wilrijk, Belgium; f Department of Biomedical Sciences, University of Antwerp, Universiteitsplein 1, 2610, Wilrijk, Belgium; g Provincial Institute of Hygiene (PIH), Kronenburgstraa 45, 2000, Antwerpen, Belgium; h Family Medicine and Population health, University of Antwerp, Universiteitsplein 1, 2610, Wilrijk, Belgium; i Department of Public Health and Primary Care, Ghent university, Corneel Heymanslaan 10, 9000, Ghent, Belgium; j BioStat, Data Science Institute, Hasselt University, Martelarenlaan 42, 3500, Hasselt, Belgium

**Keywords:** Asthma, Allergy, Airway inflammation, Oxidative stress, Immune biomarkers, Human biomonitoring, Mixture, Adolescents

## Abstract

**Background:**

Asthma and allergic diseases are among the common causes of morbidity and mortality globally. Various environmental pollutants are linked to the development of asthma and allergic diseases. Evidence on the role of oxidative stress and immune markers in the association of environmental pollutants with asthma and allergy is scant. We examined cross-sectional associations between environmental pollutants and asthma and allergy, investigated mixture effects and possible mediation by oxidative stress or immune markers.

**Methods:**

We used data from the Flemish Environment and Health Study 2016–2020 (FLEHS IV), including 409 adolescents aged 13–16 years. Fifty-four pollutants, including metals, phthalates, Di(isononyl) cyclohexane-1,2-dicarboxylate (DINCH), bisphenols, currently used and legacy pesticides, flame retardants, per- and polyfluoroalkyl substances (PFAS), polyaromatic hydrocarbons (PAHs), and polychlorinated biphenyls (PCBs) were analyzed. Outcomes were self-reported asthma, rhinitis, eczema, allergies, respiratory infection, and airway inflammation, measured through fractional exhaled nitric oxide (FeNO). Single pollutant models using multiple regression analysis and multipollutant models using Bayesian Kernel Machine Regression (BKMR) were fitted. As sensitivity analysis, Bayesian model averaging (BMA) and elastic net (ENET) models were also performed. For Bayesian models, posterior inclusion probabilities (PIP) were used to identify the most important chemicals. Mediation analysis was performed to investigate the role of oxidative stress, measured by urinary 8-hydroxy-2' -deoxyguanosine (8-OHdG), and immune markers (eosinophils, basophils, InterLeukin 8, InterLeukin 6, and Interferon-ᵧ in blood).

**Results:**

In single pollutant models, FeNO was significantly higher by 20% (95% CI: 6, 36%) and 13% (95% CI: 2, 25%) per interquartile range (IQR) fold in mono-n-butyl phthalate (MnBP) and mono-benzyl phthalate (MBzP), respectively. In BKMR analysis, the group PIPs indicated phthalates and DINCH as the most important group (group PIP = 0.509), with MnBP being the most important pollutant within that group (conditional PIP = 0.564; %change = 28%; 95%CI: 6, 54%). Similar patterns were observed in all multipollutant models. Eosinophil count mediated 37.8% (p = 0.018) and 27.9% (p = 0.045) of the association between MBzP and FeNO, and the association between MnBP and FeNO, respectively. 8-OHdG plays a significant mediating role in the association of 2,4-Dichlorophenoxyacetic acid (2,4-D) (55.4%), 3,5,6-Trichloro-2-pyridinol (TCPY) (48.1%), and 1-Naphthylamine (1-NAP) (32.7%) with rhinitis, while the total effects of these chemicals on rhinitis were not statistically significant.

**Conclusions:**

This study found associations between phthalates, MnBP and MBzP, and elevated FeNO, which appeared to be mediated by eosinophil count. 8-OHdG plays a significant mediating role in the association between 2,4-D, TCPY, and 1-NAP with rhinitis, while their direct effects remain non-significant. Use of inflammatory and oxidative stress markers can enhance the understanding of inflammatory processes in asthma and allergic diseases due to environmental pollutants.

## Highlights

• The study explored links between environmental pollutants and asthma/allergy in adolescents.
• MnBP and MBzP were positively linked to FeNO, a biomarker of airway inflammation related to asthma.
• Eosinophil count mediated the relationship between phthalates and increased FeNO.
• 8-OHdG, an oxidative stress marker, may link certain pesticides and PAHs to allergic rhinitis.

**Table:** Abbreviations

1-OH PYR	1-Hydroxypyrene
2,4-D	2,4-Dichlorophenoxyacetic acid
2-OH NAPH	2-Hydroxynaphthtalene
2-OH PHE	2-Hydroxyphenantrene
3-OH PHE	3-Hydroxyphenantrene
3-PBA	3-Phenoxybenzoic acid
4-OH PHE	4-Hydroxyphenantrene
4-OH-DPHP	4-hydroxyphenyl phenyl phosphate
5cx-MEPP	mono(2-ethyl-5-carboxy- pentyl) phthalate
5-OH-EHDPHP	2-ethyl-5-hydroxyhexyl diphenyl phosphate
5OH-MEHP	mono-2-ethyl-5-hydroxyhexyl phthalate
5oxo-MEHP	mono-2-ethyl-5-oxohexyl phthalate
8-OHdG	8-hydroxy-2' -deoxyguanosine
AMPA	Aminomethylphosphonic acid
As III	Arsene(III)
As V	Arsene(V)
AsB	Arsenobetaïne
BBOEHEP	2-hydroxyethyl bis(2-butoxyethyl) phosphate
BBOEP	bis(2-butoxyethyl) phosphate
BCIPHIPP	1-hydroxy-2-propyl bis(1-chloro-2-propyl) phosphate
BCIPP	bis(1-chloro-2-propyl) phosphate
BDCIPP	bis(1,3-dichloro-2-propyl) phosphate
BDE	Brominated diphenylether
BKMR	Bayesian kernel machine regression
BMA	Bayesian model averaging
BPA	Bisphenol A
BPAF	Bisphenol AF
BPB	Bisphenol B
BPF	Bisphenol F
BPS	Bisphenol S
BPZ	Bisphenol Z
Cd	Cadmium
Cd	Cadmium
cPIP	Conditional posterior inclusion probability
Cu	Copper
CXMIDP	mono(6-carboxy-isodecyl) phthalate
DDE	Dichloro-diphenyl-dichloroethylene
DDT	Dichloro-diphenyl-trichloroethane
DINCH	Di-(iso-nonyl)-cyclohexane-1,2-dicarboxylate
DMA	Dimethyl arsenate
DNBP	di-n-butyl phosphate
DPHP	diphenyl phosphate
EHPHP	2-ethylhexyl phenyl phosphate
ENET	Elastic net
FeNO	Fractional exhaled nitric oxide
FLEHS	Flemish Environment and Health Study
Gly	Glyphosate
gPIP	Group posterior inclusion probability
HCB	Hexachlorobenzene
IFN-ᵧ	Interferon gamma
IL	Interleukin
ISCED	International Standard Classification of Education
LOD	Limit of detection
LOQ	Limit of quantification
MBzP	mono-benzyl phthalate
MCOCH	cyclohexane-1,2-dicarboxylic mono carboxyisooctyl ester
MCOCH	cyclohexane-1,2-dicarboxylic mono carboxyisooctyl ester
MCOP	mono(7-carboxy-isononyl) phthalate
MEHA	mono(2-ethylhexyl) adipate
MEHP	mono-2-ethylhexyl phthalate
MEHTP	mono(2-ethylhexyl) terephthalate
MEP	Monoethyl phthalate
MHNCH	cyclohexane-1,2-dicarboxylic mono hydroxyisononyl ester
MHNP	mono(7-hydroxy-isononyl) phthalate
MiBP	mono-isobutyl phthalate
MINCH	cyclohexane-1,2-dicarboxylic mono isononyl ester
MMA	Mono methyl arsenate
Mn	Manganese
MnBP	mono-n-butyl phthalate
OHMEHA	mono(2-ethyl-5-hydroxyhexyl) adipate
OHMEHTP	mono(2-ethyl-5-hydroxyhexyl) terephthalate
OHMIDP	mono(6-hydroxy-isodecyl) phthalate
OH-TPHP	hydroxyphenyl diphenyl phosphate
OXC	Oxychlordane
OXOMIDP	mono(6-oxo-isodecyl) phthalate
PAH	Polycyclic aromatic hydrocarbon
Pb	Lead
PCB	Polychlorinated biphenyls
PFAS	Per- and Polyfluoroalkyl Substances
PFBS	perfluorobutaansulnoic acid
PFDA	perfluorodecnoic acid
PFDoDA	perfluorododecaanoic acid
PFHpA	perfluoroheptanoic acid
PFHpS	perfluoroheptaansulfnoic acid
PFHxA	perfluorohexanoic acid
PFHxS	perfluorohexaansulfnoic acid
PFNA	perfluorononnoic acid
PFOA	perfluorooctnoic acid
PFOS	perfluorooctaansulnoic acid
PFPeA	perfluoroplonganoic acid
PFUnDA	perfluoroundecaanoic acid
PIP	Posterior inclusion probability
SDEHTM	di(2-ethylhexyl) trimellitate
SG	Specific gravity
t,t’-MA	T,t'-muconic acid
TC	Total cholesterol
TCEP	tris(chloroethyl) phosphate
TCPᵧ	3,5,6-Trichloro-2-pyridinol
TG	Triglycerides
Tl	Thallium
TN	Trans-nonachlor
β-HCH	Beta hexachlororcyclohexane

## 1 Background

Asthma and allergic diseases are prevalent conditions affecting millions of people worldwide, impacting quality of life and, in severe cases, posing significant health risks (World Health Organization). Asthma is a chronic condition characterized by inflammation of airways, leading to symptoms such as wheezing, coughing, and shortness of breath (World Health Organization, 2024). It is a significant cause of hospitalizations and emergency visits (Kang et al., 2023). Allergic rhinitis involves an immune response to allergens that leads to swelling of nasal mucosa and excessive mucus production, resulting in symptoms resembling common cold, such as runny nose, sneezing, and itching. Eczema manifests as inflamed, itchy, and dry skin due to an immune response. In 2019, the global prevalence of asthma was estimated to exceed 260 million cases, while atopic dermatitis (eczema) affected over 170 million individuals (Shin et al., 2023a), posing a substantial burden on healthcare systems worldwide. Allergic rhinitis is the most common airway disease and most costly respiratory condition at population level due to high prevalence (Dierick et al., 2020).

The development of asthma and allergic diseases involves a complex interplay between genetic and environmental factors. Various environmental pollutants may increase the risk of developing asthma and allergy and/or aggravate symptoms (Thomsen, 2015). Exposure to allergens from trees and grasses (pollen), mold, animals such as cats and dogs, insects, and pollutants are environmental risk factors (Murrison et al., 2019). Although findings are mixed, exposure to per- and polyfluoroalkyl substances (PFAS), persistent pollutants most widely used in industry and consumer products, are related to immune responses, asthma and allergy related diseases (Kvalem et al., 2020). Systemic and inhaled PFAS are found to trigger pulmonary pro-inflammatory responses (Ryu et al., 2014). However, epidemiological studies show inconsistent results, with some studies on children and adolescents reporting no significant associations between PFAS and asthma or allergies (Rappazzo et al., 2017; Gaylord et al., 2019), while few studies reported a significant positive association (Jackson-Browne et al., 2020; Averina et al., 2019) or a significant inverse relationship (van Larebeke, 2023). Similarly, some phthalates, a group of pollutants mainly used as plasticizers, might increase the risk of asthma and allergies through pathological changes or exacerbate already-existing conditions and increase severity of symptoms (Franken et al., 2017; Zhou et al., 2020). Exposure to metals including cadmium, molybdenum, copper, chromium, and selenium is suggested to be linked with increased incidence of asthma and allergic symptoms in children (Gasana et al., 2012; Huang et al., 2016). Limited epidemiological studies have examined the association of pesticides, including organophosphates, organochlorines and pyrethroids, with asthma, yielding inconsistent findings (Ratanachina et al., 2020; Ye et al., 2016). Exposure to PCBs during the prenatal period has also been linked to increased risk of eczema in childhood (Parker-Lalomio, 2018; Hara, 1985). Moreover, exposure to polyaromatic hydrocarbons (PAHs) has been linked to inflammation and impairment of lung function (Mattila et al., 2021). Moreover, PAHs are found to induce oxidative stress (Gammon et al., 2008), which could lead to lung inflammation.

Various biological mechanisms have been shown to play a role in the pathogenesis of asthma including inflammation, immune modulation, oxidative stress, and epithelial and endothelial dysfunctions (Karimi et al., 2015). Internal exposure to environmental pollutants, such as dioxins, metals, PFAS, and phthalates, has been shown to increase the production of reactive oxygen species (ROS), leading to oxidative stress and DNA damage (Omoike et al., 2021; Brassea-Pérez, 2022). Oxidative stress may underlie various physiological and pathological processes, which in turn might result in systemic inflammation and chronic diseases (Verheyen et al., 2021). While the role of oxidative stress has been extensively studied in diseases like diabetes mellitus, cardiac diseases, cancer, and neurodegenerative disorders (Senoner et al., 2019; Hayes et al., 2020), limited evidence exists regarding its involvement in the development of asthma and allergies (Franken et al., 2017). Likewise, exposure to environmental pollutants is related to adverse effects on the immune system (Rogers et al., 2021; Suzuki et al., 2020). Environmental pollutants and chemicals can trigger immune responses, resulting in the production of immune markers like cytokines, chemokines, and immunoglobulin E (IgE) (Ehrlich et al., 2023). These markers contribute to airway inflammation, bronchoconstriction, and mucus overproduction, all of which are characteristic of asthma. Pollutant exposure also disrupts immune function and induces oxidative stress, further driving inflammation (Suzuki et al., 2020). Due to chronic inflammation, oxidative stress and immune markers may play a crucial role in the development and progression of asthma and other allergic diseases (Kim et al., 2007; Lombardi et al., 2022). Thus, it is necessary to investigate the role of oxidative stress and immune markers in the association between environmental pollutants and asthma and allergic diseases. 8-hydroxy-2-deoxyguanosine (8-OHdG), a urinary product of oxidative damage to 2′-deoxyguanosine, is stabile in urine, making it a sensitive and important biomarker for oxidative stress (Valavanidis et al., 2009).

Many studies primarily focus on assessing the effects of individual pollutants, providing limited knowledge about the impact of exposure to multiple pollutants. In real life, people are exposed to multiple pollutants simultaneously, and it is crucial to investigate individual and multipollutant associations with the occurrence of asthma and allergy. Therefore, this study examined the association of a mixture of pollutants on asthma, exhaled nitric oxide (FeNO), and allergy-related health outcomes, and evaluated the individual contributions of each pollutant to the overall mixture association. We also investigated the mediation role of oxidative stress and immune markers in the association between environmental pollutants and asthma and allergy.

## 2 Methods and materials

### 2.1 Study setting and population

This study used data from the Flemish Environment and Health Study 2016–2020 (FLEHS IV). The sampling process took place between September 2017 and June 2018, employing a two-stage clustered stratified sampling procedure. The first stage involved stratification based on provinces of Flanders. The number of participants was proportional to the number of inhabitants per province. The second stage sampling units were schools, randomly selected within each province. To improve representativeness in terms of geographical coverage, schools had to be at least 20 km apart, and to ensure representation of all socio-economic categories, one school with a higher proportion of socially deprived students was included in each province. A total of 20 schools were selected across 5 provinces. Inclusion criteria were: 1) participants needed to reside in Flanders for at least five years, and 2) study participants and parents needed to have sufficient proficiency in Dutch to complete questionnaires. Exclusion criteria were: 1) failure to complete all questionnaires, 2) missing blood and urine samples, 3) repeating a school year more than once, or 4) attending a boarding school. Further details about the study are available elsewhere (Schoeters et al., 2022). A total of 428 adolescents aged 13–16 years participated in the FLEHS IV study. Among them, 19 either reported as current smokers or had missing data on smoking and were excluded from this analysis, resulting in a final sample size of 409 participants.

### 2.2 Sample collection and processing

During the clinical examination, spot urine and blood samples were obtained. Urine samples were stored in clean, metal-free polyethylene containers at 4 °C and processed for further storage within 24 h. Polypropylene tubes were used for measuring biomarkers related to benzene, PAHs, and arsenic species, whereas metal-free polyethylene tubes were used for measurement of other metal biomarkers. Arsenic species were measured in urine samples that were stored at 4 °C and analyzed within 24 h. All samples, except those for benzene and 8-OHdG biomarkers, were stored at −20 °C until analysis. The samples for benzene biomarkers were kept at −80 °C. Blood samples were immediately processed, and separate portions of whole blood and serum were obtained. These aliquots were carefully preserved at 4 °C and then stored either at −20 °C or −80 °C within 12 h in a centralized laboratory (Flemish Institute for Technological Research (VITO), Belgium). For blood cell count measurement, samples were stored at 4 °C and analyzed within 24 h. After the completion of the field work, all samples, along with field work blanks and control samples, were shipped to the analytical laboratories for analysis.

### 2.3 Measurement of exposure biomarkers

#### 2.3.1 Biomarkers measured in urine

Metals including inorganic arsenic (As), cadmium (Cd) and thallium (Tl) were measured in urine. Metabolites of PAHs and benzene were also measured in urine. Plastic compounds including metabolites of phthalates, DINCH, bisphenols and organophosphate flame retardants (OPFRs) were measured in urine. To assess exposure to currently used pesticides, biomarkers of pyrethroids, chlorpyrifos, phenoxy herbicide 2,4-dichlorophenoxyacetic acid (2,4-D) and glyphosate (GLY) and its metabolite aminomethylphosphonic acid (AMPA) were measured in urine.

#### 2.3.2 Biomarkers measured in blood

Metals including copper (Cu), lead (Pb), and manganese (Mn) were measured in whole blood. Legacy pesticides, such as beta-and gamma-hexachlorocyclohexane (β-HCH and γ-HCH), p,p’-dichloro-diphenyl-trichloroethane (DDT) and metabolites were measured in serum samples. PFAS, markers of PCBs and polybrominated diphenyl ethers (PBDEs) were measured in blood serum samples. Details of the measured pollutants, analytical methods and limits of detection and quantification are available elsewhere (Schoeters et al., 2022) and in the supplement (Table S1).

Total cholesterol (TC) and triglycerides (van Larebeke, 2023) were also measured in blood serum. The total lipid (TL) concentration was calculated using the formula: TL(mg/dL)=2.27∗TC+TG+62.3mg/dL (Bernert et al., 2007), and was used to standardize lipid-soluble serum biomarkers (${biomarker}_{lipid}=100*\;{biomarker}_{meas}/lipid$). Likewise, specific gravity was determined in urine and urinary biomarker concentrations were normalized for SG using the following formula: ${biomarker}_{SG\;}={biomarker}_{meas}*\left(1.024-1\right)/\left(SG-1\right)$, where ${biomarker}_{SG}$ is the normalized biomarker concentration, ${biomarker}_{meas}$ is the measured biomarker concentration per liter urine and SG as the specific gravity of the urine sample (Pearson et al., 2009).

#### 2.3.3 Assessment of health outcome and effect biomarkers

Before clinical examination, teenagers filled out questionnaires on health status and lifestyle patterns. The presence/absence of asthma (last year), rhinitis (ever), eczema (ever), skin allergy to products (last 5 years), any kinds of allergy (food, medicines, insect bites, metal, care products, household and maintenance products) (last 5 years), and respiratory infection (last year) were obtained from a questionnaire adapted from the International Study of Asthma and Allergies in Childhood (ISAAC) (Asher et al., 1995). The questions and algorithm used to determine health outcomes is available in the supplementary materials (Table S3). Furthermore, FeNO was measured using a breath test with the NIOX Vero device (Circassia AB, Belgium).

#### 2.3.4 Assessment of mediators (oxidative stress and immune markers)

The level of 8-OHdG was determined in urine using a competitive enzyme-linked immunosorbent assay (ELISA) kit (Japan Institute for the Control of Aging, Shizuoka, Japan), according to manufacturer's instructions. The determination range was 0.5–200 ng/mL and the anti-8-oxodG mouse monoclonal antibody (clone N45.1) was used as a primary antibody, which has an established specificity (Toyokuni et al., 1997). The values from each urine sample were calculated based on calibration sigmoid plots of absorbance (450 nm) of an 8-oxodG standard at various concentrations.

Leukocyte count and leukocyte subtype (neutrophils, lymphocytes, monocytes, eosinophils, and basophils) distribution (percentage) were assessed using a Sysmex XE-2100 instrument for hematology analysis (Sysmex Corporation, Kobe, Japan), a widely used automated hematology system that combines flow cytometry with fluorescence detection, using a diode laser bench (Nakul-Aquaronne, 2003). Counts of leukocyte subtypes were subsequently calculated by multiplying the subtype fraction with the total leukocyte count. To determine cytokine levels, a validated pro-inflammatory cytokine panel from MSD was selected (Meso Scale Discovery, Rockville, MD, USA), consisting of nine cytokines that play an important role in the immune response: interferon-gamma (IFN-ᵧ), tumor necrosis factor alpha (TNF-_α_) and the interleukins IL-2, IL-4, IL-6, IL-8, IL-10, IL-12, IL-13. Individual cytokine concentrations were determined using a high-performance immunoassay (MSD MESO QuickPlex) (MESO QuickPlex SQ 120, 2024). Each sample was measured twice according to the manufacturer's protocol to optimize the accuracy of the measurement results and expressed as picograms per milliliter of serum (pg/mL) and the average of the two measurements is used for the statistical analyses.

#### 2.3.5 Covariate assessment

Before sample collection, adolescents and their parents filled out questionnaires on health status and lifestyle patterns. Participant age, sex, highest educational level of the household (the highest of either of the parents), passive smoking (being in the house or elsewhere where people smoke at least once a week) and other relevant participant characteristics were obtained from questionnaires. The classification of the highest educational level of the household was based on the International Standard Classification of Education (ISCED) developed by the United Nations Educational, Scientific and Cultural Organization (Statistics, 2012). Low education was defined as no secondary to lower secondary education (ISCED level 0–2), medium education as having attained upper secondary to post-secondary non-tertiary education (ISCED level 3–4), and high education as having attained tertiary education or higher (ISCED level ≥5).

#### 2.3.6 Statistical analysis

We assessed the distribution of variables, including measures of exposures, mediators, and effect biomarkers. To mitigate distributional skewness, we applied natural log-transformation to exposure biomarkers, mediators, and FeNO. Exposure biomarkers and mediators with detection rates below 70% were excluded from further analysis. As a result, a total of 54 exposure biomarkers including 5 metals, 3 pesticides, 6 PCBs, 6 Organochlorine (OC) pesticides, 4 PFAS, 4 PAHs, 1 benzene metabolite - trans,trans-muconic acid, 6 OPFRs, 3 bisphenols, 16 phthalates, DINCH & alternative plasticizers, and 5 mediators (8-OHdG, eosinophils (total), basophils (total), IL8, IFN-ᵧ) were included in the present analysis. Limit of detection/Limit of quantification (LOD/LOQ) and percent above the limit are available in the supplementary material (Tables S1 and S2). For exposures and mediators included in the analysis, values below the LOD/LOQ were imputed using single random imputation from a censored lognormal distribution. Urinary exposure markers and 8-OHdG were normalized for SG and lipid-soluble blood markers (PCBs and BDEs) were standardized for total lipid using this formula: ${biomarker}_{lipid}=100*\;{biomarker}_{meas}/lipid$).

Characteristics of participants and health outcomes were summarized using absolute and relative frequencies for categorical variables and median with 1st (P25) and 3rd quartile (P75) for continuous measures. Exposure biomarkers and mediators were also summarized using median with P25 and P75. To describe the correlations between concentrations of exposure biomarkers, pairwise Pearson correlations were computed on ln-transformed values.

Associations between pollutant concentrations and FeNO were assessed using (single pollutant) linear regression models, adjusted for covariates, age (in years), sex, ISCED (low/medium/high), and passive smoking (yes/no), which were selected based on Directed Acyclic Graph (DAG) (Fig. S1). In addition, in case of pollutants measured in urine, the model incorporated SG, while for lipid-soluble biomarkers, total lipid was included as a covariate as suggested by O'Brien KM et al. (O'Brien et al., 2016). Ln-transformed pollutant concentrations and FeNO were used in the regression analysis. Associations between exposures and binary health outcomes were assessed using logistic regression adjusted for the same set of covariates.

In addition to single pollutant models, we performed multi-pollutant analysis using Bayesian kernel machine regression (BKMR). BKMR is a non-parametric approach that models the exposure-response relationship using a kernel function that considers potential interactions between exposures and captures possible nonlinear associations between exposure and outcomes (Bobb et al., 2015). The BKMR model was performed with at least 50,000 iterations by Markov chain Monte Carlo (MCMC) sampler with a hierarchical selection to group of pollutants a priori using the bkmr package in R. We used a Gaussian distribution for continuous outcome (FeNO) and a binomial distribution with a probit link function for binary health outcomes. Models were adjusted for the same set of covariates as in the single-pollutant models. We estimated the group (gPIP) and conditional (within-group) posterior inclusion probabilities (cPIP), the univariate exposure-outcome relationships with all other exposures fixed to their 50th quantile, and the overall pollutant mixture association with asthma and allergy related outcomes. To check for consistency, we performed additional multi-pollutant models using Bayesian Model Averaging (BMA) (Clyde et al., 2011) and elastic net (ENET) models (Zou et al., 2005). BMA is an algorithm for Bayesian variable selection and model averaging that operates on sampling without replacement. The BMA algorithm calculates marginal posterior inclusion probabilities (PIPs) for each pollutant. The analysis employed the R package BAS, utilizing the Jeffreys-Zellner-Siow prior for regression coefficients (Liang et al., 2008). To obtain estimates and 95% Bayesian credible intervals (CrI), the full posterior distribution of all regression coefficients was employed. ENET is a penalization approach incorporating regularization techniques of both lasso and ridge regression (Agier et al., 2016). By leveraging the strengths of both methods, ENET achieves a balanced regularization effect. To determine the optimal level of penalization, a 10-fold cross-validation error minimization approach was employed, followed by stability selection to allow finite sample control of error rates (PFER = 0.5), and mixing parameter (α) was set at 0.5. R packages glmnet (Friedman et al., 2010) and stabs (Hofner et al., 2017) were used for ENET analysis and stability selection, respectively.

We examined the conditions of mediation analysis: i) exposure association with mediator, and ii) mediator with the outcome (Richiardi et al., 2013). For those that satisfied the conditions, we explored the mediating role of oxidative stress and immune markers on the association of environmental pollutants with FeNO and health outcomes using medflex package in R (Steen et al., 2017). The medflex package is based on fitting natural effect models, which parameterize both direct and indirect effects. The mediation analysis was performed for single pollutant-outcome associations adjusting for the same set of covariates.

Estimated regression coefficients are presented as the percentage change in FeNO per interquartile range (IQR) change in pollutant concentrations. For binary health outcomes, coefficients are presented as the Odds Ratio (OR) for an IQR increase in pollutant concentrations. All analyses were performed in R version 4.3.1 (R Co re Team and R, 2022).

## 3 Results

### 3.1 Descriptive statistics

#### 3.1.1 Characteristics of study participants

Table 1 shows characteristics of the study participants. Of a total of 409 participants, the median age was 14.8 (P25: 14.5, P75: 15.1), 46% were boys, and 62% were from a household ISCED level of above 5. Over one-fourth (27%) of the participants were exposed to passive smoking. The most prevalent health outcome was rhinitis (26.8%), followed by skin allergy (24.0%), eczema (21.9%) and asthma (13.8%). The median FeNO and 8-OHdG were 12 ppb (P25: 7, P75: 20) and 17 μg/L (13, 22) respectively. The median eosinophil and basophil count was 141 per μL (85, 250) and 28 per μL (19, 39) respectively.

**Table 1:** Characteristics of adolescents (13–16 years) in FLEHS IV (2016–2018) included in this study (n = 409).

Characteristics	N	n (%)/Median (P25, P75)
**Sociodemographic and behavioral**		
Age (years)	409	14.8 (14.5, 15.1)
Sex	409	
Boy		188 (46.0)
Girl		221 (54.0)
Household education level	401	
ISCED level 0–2		25 (6.2)
ISCED level 3–4		128 (31.9)
ISCED level ≥5		248 (61.8)
Passive smoking	398	109 (27.2)
**Health outcomes and effect biomarker**		
Asthma (last year)	400	55 (13.8)
Rhinitis (ever)	400	107 (26.8)
Eczema (ever)	362	88 (21.9)
Skin allergy to products (last 5 years)	353	87 (24.0)
Allergy of any type^a^ (last 5 years)	353	143 (40.5)
Respiratory infection (last year)	382	44 (11.5)
FeNO (ppb)	406	12 (7, 20)
**Oxidative stress and immune markers**		
8-OHdG (μg/L) (normalized for SG)	396	17 (13, 22)
Total basophil (n/μL)	396	28 (19, 39)
Total eosinophil (n/μL)	397	141 (85, 250)
IL8 (pg/mL)	361	7 (5, 11)
IL6 (pg/mL)	361	0.34 (0.19, 0.49)
IFN-ᵧ (pg/mL)	361	4 (3, 5)

ISCED: International Standard Classification of Education; FeNO: Fractional exhaled nitric oxide; 8-OHdG: 8-hydroxy-2' -deoxyguanosine; IL6: Interleukin-6; IL8: Interleukin-8; IFN-ᵧ: Interferon gamma.

a Allergy; allergy to food, medicines, insect bites, metal, care products, household, maintenance products.

#### 3.1.2 Exposure levels

Table 2 shows exposure biomarker concentrations of the study participants. The metal with the highest blood concentration was Cu, with a median of 795 μg/L, followed by Mn (M = 9.35 μg/L). TCPY was the highest exposure pollutant observed within the group of pesticides, with a median of 4.5 μg/L. PCB138 was the highest among all PCBs with a median of 6.9 ng/g lipid. The highest organochlorine (OC) pesticide was p,p'-DDE (M = 36 ng/g lipid), followed by HCB (M = 7.6 ng/g lipid). For the PFAS compounds, the highest concentration was observed for PFOS (M = 2.10 μg/L), whereas within PAHs, 2-NAP had the highest exposure concentration, (M = 3.7 μg/L). The median concentration was 101 μg/L for TTMA and 1.14 μg/L for BPA. EHPHP had the highest exposure concentration from the OPFRs (M = 4.1 μg/L). Among biomarkers of phthalate, the highest concentration was found for MEP (M = 27 μg/L), whereas the highest observed DINCH was MHNCH (M = 1.16 μg/L).

**Table 2:** Biomarker levels measured in FLEHS IV adolescents normalized to specific gravity (urinary markers) or standardized for serum lipids (lipid-soluble serum biomarkers) (n = 409).

Group	Exposure	Median (P25, P75)
Metals (μg/L)	Pb (blood)	7.59 (5.96, 9.40)
	Mn (blood)	9.35 (7.90, 11.26)
	Cu (blood)	795 (724, 877)
	Cd (urine)	0.29 (0.23, 0.39)
	Tl (urine)	0.36 (0.29, 0.43)
Pesticide (μg/L)	3-PBA	0.87 (0.58, 1.58)
	2,4-D	0.27 (0.15, 0.45)
	TCPY	4.5 (2.9, 6.4)
PCBs (ng/g lipid)	PCB118	2.13 (1.55, 2.95)
	PCB153	10 (6, 16)
	PCB138	6.9 (4.7, 10.1)
	PCB187	1.10 (0.65, 1.89)
	PCB180	4.3 (2.7, 7.3)
	PCB170	2.00 (1.24, 3.26)
OC pesticides (ng/g lipid)	OXC	1.18 (0.82, 1.74)
	TN	0.80 (0.51, 1.17)
	p,p'-DDE	36 (24, 63)
	p,p'-DDT	2.4 (1.1, 4.5)
	HCB	7.6 (5.7, 9.8)
	β-HCH	1.13 (0.81, 1.50)
PFAS (μg/L)	PFOA	1.10 (0.84, 1.30)
	PFNA	0.31 (0.23, 0.44)
	PFHxS	0.48 (0.35, 0.65)
	PFOS	2.10 (1.40, 3.15)
PAH (μg/L)	1-NAP	0.07 (0.05, 0.09)
	2-NAP	3.7 (2.1, 6.9)
	2-PHEN	0.07 (0.05, 0.10)
	3-PHEN	0.07 (0.05, 0.10)
Benzene (μg/L)	TTMA	101 (57, 146)
OPFRs (μg/L)	DPHP	1.32 (0.88, 2.05)
	BDCIPP	0.34 (0.15, 0.69)
	BCIPHIPP	0.65 (0.30, 1.47)
	EHPHP	4.1 (2.8, 6.7)
	BBOEHEP	0.04 (0.02, 0.07)
	5-OH-EHDPHP	0.09 (0.06, 0.16)
Bisphenol (μg/L)	BPA	1.14 (0.68, 1.91)
	BPF	0.15 (0.08, 0.30)
	BPS	0.14 (0.07, 0.22)
Phthalates, DINCH & other alternative plasticizers (μg/L)	MEP	27 (15, 71)
	5cx-MEPP	16 (12, 22)
	5OH-MEHP	6.6 (4.3, 10.1)
	MiBP	22 (15, 41)
	MnBP	19 (12, 31)
	5oxo-MEHP	4.1 (2.8, 6.6)
	MBzP	2 (1, 6)
	MEHP	1.32 (0.83, 2.13)
	OHMEHTP	0.58 (0.35, 1.02)
	MHNP	4.3 (2.8, 6.5)
	MCOP	1.88 (1.29, 2.84)
	MHNCH	1.16 (0.72, 2.28)
	MCOCH	1.07 (0.74, 1.68)
	OHMIDP	0.73 (0.45, 1.21)
	CXMIDP	1.25 (1.06, 1.58)
	OXOMIDP	0.41 (0.28, 0.68)

Highest correlations were observed for exposures within pollutant groups, mainly PCBs (from *r* = 0.62 to 0.98). Within phthalates, high correlation was observed between 5OH-MEHP and 5oxo-MEHP (*r* = 0.97) followed by 5oxo-MEHP and 5cx-MEPP (*r* = 0.79). Detailed correlation and heatmap plots are available in the supplementary material (Fig. S2). There were only weak correlations between oxidative stress and the immune markers, except between eosinophil and basophil (r = 0.26) (Fig. S3)

#### 3.1.3 Association of exposure biomarkers with asthma and allergy related outcomes

In a single pollutant model adjusted for covariates, the estimated FeNO was significantly higher by 20% (95% CI: 6, 36%) per IQR increase in MnBP. Likewise, each IQR increase in MBzP was significantly associated with 13% (95%CI: 2, 25%) higher level of FeNO. No other pollutants were significantly associated with FeNO level. The odds of having eczema were significantly higher for each IQR increase in PCB153 (OR: 1.54; 95%CI: 1.05, 2.27), PCB180 (OR: 1.52; 95%CI: 1.04, 2.21), PCB170 (1.50; 95%CI: 1.03, 2.18), OXC (OR: 1.40, 95%CI: 1.03, 1.92), and TN (OR: 1.52, 95%CI: 1.10, 2.12). In contrast, the odds of asthma were significantly lower per IQR increase in PCB118 (OR: 0.58; 95%CI: 0.38, 0.88), PCB153 (OR: 0.58; 95%CI: 0.36, 0.93), and PCB138 (OR: 0.56; 95%CI: 0.34, 0.88). The odds of rhinitis were significantly lower per IQR increase in Tl (OR: 0.67; 95%CI: 0.48, 0.92), p,p'-DDT (OR: 0.79; 95%CI: 0.63, 1.00), MEP (OR: 0.72; 95%CI: 0.52, 0.99), MHNCH (OR: 0.64; 95%CI: 0.46, 0.86), and MCOCH (OR: 0.70; 95%CI: 0.50, 0.95). Detailed results of the single pollutant regression analyses are available in the supplementary material (Table S4).

#### 3.1.4 Association of exposure mixture with asthma and allergy related outcomes

Upon checking for correlation, one of the correlated exposure markers with *r* ≥ 0.90 was excluded from the multipollutant analysis. As a result, PCB153, PCB180, PCB138, MCOCH, 5oxo-MEHP were excluded from the mixture analyses. Therefore, BKMR, BMA, and ENET were used to analyze a total of 49 pollutants. For FeNO, using BKMR the group PIPs indicated phthalates and DINCH as the most important group (gPIP = 0.509), with MnBP being the most important pollutant within group (cPIP = 0.564, %change = 28% (95%CI: 6, 54%)). Results of BMA also showed that phthalates and DINCH are the most important group (gPIP = 0.305), and MnBP has the greatest importance within the group (cPIP = 0.752; %change = 3%; 95%CI: 0, 25%)). In the ENET model, MnBP showed the highest selection probability (0.70). For asthma, the BKMR model showed that PCBs (gPIP = 0.758) were the most important group, followed by bisphenols (gPIP = 0.479) and OC pesticides (gPIP = 0.471). The conditional-PIPs show that PCB118 (cPIP = 0.746), BPS (cPIP = 0.522) and HCB (0.279) had the greatest importance within respective pollutant groups. In the BMA analysis, PCBs were the most important pollutant groups (gPIP = 0.224). For rhinitis, using BKMR, metals showed the greatest group importance (gPIP = 0.705), with Cu being the most important pollutant within group (cPIP = 0.609). The BMA showed phthalates and DINCH are the most important group (gPIP = 0.247), and MHNCH the most important pollutant within group (cPIP = 0.766). The ENET model showed MHNCH as the most important with selection probability of 0.640. Further details of mixture analyses results and plots are available in the supplementary material (Tables S7–S13, Figs. S4–S13).

#### 3.1.5 Mediating role of 8-OHdG and immune markers

For FeNO, two exposure biomarkers (MBzP and MnBP) and one mediator (total eosinophil) met the conditions for mediation analysis. The results of the mediation analysis indicated that total eosinophil mediated 37.8% of the positive association between MBzP and FeNO (p = 0.018). Both direct and mediated associations are positive; however, the direct path is not statistically significant (p = 0.143). Similarly, total eosinophil mediated 27.9% of the positive association between MnBP and FeNO (p = 0.045). Both the direct and mediated associations are significantly positive (Table 3). Regarding health outcomes, three exposure biomarkers (2,4-D, TCPY, and 1-NAP), 1 mediator (8-OHdG), 1 outcome (rhinitis) met the condition for mediation analysis. The total effect of these exposure markers with rhinitis was not statistically significant. However, the statistically significant association between exposure biomarkers and mediators, as well as between mediators and outcomes, prompted further investigation through mediation analysis. The result showed that 8-OHdG significantly mediated 55.4%, 48.1%, and 32.7% of the association of 2,4-D, TCPY, and 1-NAP, respectively, with rhinitis. In all three cases, the direct effects were negative but not statistically significant, while the mediated effects were positive and significantly associated with rhinitis (as shown in Table 3). For a more comprehensive understanding of the mediation analysis results, we refer to the supplementary material (Tables S5 and S6).

**Table 3:** The mediating role of eosinophils and 8-OHdG in the association between exposure biomarkers and FeNO and rhinitis.

Outcome	Mediator	Exposure	Effect	% change per IQR (95%CI)	P value	Proportion mediated
FeNO	Eosinophil	MBzP	Direct	8 (−3, 200)	0.143	37.8%
			Mediated	5 (1, 9)	0.018	
			Total	13 (2, 25)	0.015	
		MnBP	Direct	15 (1, 258)	0.027	27.9%
			Mediated	5 (0.1, 201)	0.045	
			Total	20 (6, 36)	0.005	
				**OR (95% CI)**		
Rhinitis	8-OHdG	2,4-D	Direct	0.75 (0.56, 1.01)	0.056	55.4%
			Mediated	1.11 (1.01, 1.21)	0.025	
			Total	0.82 (0.61, 1.11)	0.197	
		TCPY	Direct	0.81 (0.56, 1.16)	0.239	48.1%
			Mediated	1.07 (1.01, 1.15)	0.034	
			Total	0.89 (0.67, 1.19)	0.430	
		1-NAP	Direct	0.73 (0.48, 1.12)	0.149	32.7%
			Mediated	1.08 (1.01, 1.16)	0.033	
			Total	0.86 (0.65, 1.13)	0.282	

CI: confidence interval; IQR: Interquartile range; OR: odds ratio.

## 4 Discussion

This cross-sectional analysis was performed using the FLEHS IV data aiming to explore the association of multiple environmental pollutants on asthma and allergy outcomes among adolescents and investigate the role of mediators. Our findings from a single analysis highlighted that phthalates, mainly MnBP and MBzP, were significantly associated with elevated FeNO, which is a biomarker of airway inflammation related to asthma. The multi-pollutant analysis also indicated that MnBP exhibits consistently positive associations with FeNO in various mixture analysis methods. Regarding other health outcomes, the single pollutant analysis revealed a higher risk of eczema with an increase in PCBs particularly PCB153, PCB180, PCB170, and OC pesticides including OXC and TN. In contrast, there was an inverse association observed between exposure to PCBs, specifically PCB118, PCB153, and PCB138, and the risk of asthma. Furthermore, Tl, p,p'-DDT, MEP, MHNCH, and MCOCH exhibited inverse associations with rhinitis. However, all these associations with health outcomes did not reach significance in multi-pollutant analysis. In the mediation analysis, we found that eosinophil count significantly mediated the association of MnBP and MBzP with FeNO. Likewise, 8-OHdG significantly mediated the association of 2,4-D, TCPY and 1-NAP with rhinitis.

Epidemiological studies have used fractional exhaled nitric oxide (FeNO) as a biomarker of airway inflammation in response to air pollutants (McCreanor et al., 2007; Delfino et al., 2006). The present study showed that phthalates, particularly MnBP and MBzP, were significantly positively associated with elevated FeNO. MnBP remained significant in the multi-pollutant analysis indicating that it is an independent predictor. Previous studies have also shown that phthalates are positively associated with FeNO in children (Just et al., 2012) and adults (Wu et al., 2022). These results suggest that exposure to phthalates is associated with a biomarker of airway inflammation among adolescents. Primary exposure to phthalates among adolescents and adults occurs through the consumption of foods and drinks that contain phthalates due to packaging or processing, as well as inhalation of particles in the air. Consequently, reducing exposure sources could be beneficial in lowering the risk of airway inflammation, particularly for individuals with a higher susceptibility to asthma.

The present study found that eosinophil count significantly mediated the association of MnBP and MBzP with FeNO. Consistently, a previous study showed that absolute eosinophil count mediated the association between another phthalate, diethyl phthalate (DEP), and lung function (Wang et al., 2021). Elevated immune markers particularly eosinophils have been linked to exposure to phthalates (Jaakkola et al., 2008; Shin et al., 2023b). Phthalates may trigger oxidative stress and disrupt endocrine pathways, which can activate cytokines (Franken et al., 2017; Zhou et al., 2020). Cytokines promote the production and migration of eosinophils, key immune cells involved in allergic and respiratory inflammation, into the airways (Chan et al., 2019). When eosinophils accumulate, they produce nitric oxide (NO) through inducible nitric oxide synthase (iNOS), which elevates fractional exhaled nitric oxide (FeNO) levels—a marker of airway inflammation (Zamora et al., 2000). This mediation pathway provides an insight into the underlying mechanisms through which phthalate exposure influences airway inflammation, highlighting the potential role of eosinophils. Understanding the pathway could help to develop targeted interventions aimed at halting the impact of environmental pollutants on respiratory health. Future studies should explore additional mediators and elucidate multiple pathways linking phthalate exposure to airway inflammation.

The present study indicated 8-OHdG significantly mediated the association of pesticides 2,4-D and TCPY on rhinitis although the total effect did not reach statistical significance. This finding implies a crucial role of oxidative stress in the pathway between pesticide exposure and rhinitis development. Several pesticides such as chlorpyrifos, 2,4-dichlorophenol, deltamethrin, and paraquat have been shown to induce oxidative stress (Makris et al., 2022). Activation of nuclear factor kappa-light-chain-enhancer of activated B cells (NF-κB) and thioredoxin-interacting protein (TXNIP) are possible pathways of oxidative stress leading to inflammatory processes in allergic rhinitis (Han et al., 2021). However, the contrasting directions of direct and mediated effects introduce complexity, which could be due to the involvement of intricate biological mechanisms or a result of unaccounted confounding variables. Thus, further research is needed to elucidate the interplay between pesticide exposure, oxidative stress, and rhinitis.

Although PCBs were banned from production worldwide in 2001 (United Nations Environmental Programme, 2008), due to their properties of a long half-life and fat solubility, they are still preserved in soil, water, and food chain, and consequently in human tissues (Domingo, 2012). The present study reveals an association between PCBs (PCB153, PCB180, PCB170) and OC pesticides (OXC, TN), and an increased risk of eczema in single pollutant analysis. This suggests a potential role for these pollutants in the development or exacerbation of eczema. However, the significance of this association diminishes in multipollutant analysis. This discrepancy underscores the complexity of environmental exposures and their effects on health outcomes. It's possible that the observed association in the single pollutant analysis is influenced by confounding factors or interactions between pollutants that are not captured when analyzing them individually. Further research is needed to investigate the complex relationships between multiple pollutants and their mixture effect on eczema risk.

The main strength of this study is using various methods of single and multi-pollutant regression approaches to explore the possible associations. In addition, this study explored the association of a wide range of pollutants with asthma and allergy outcomes, which provides a basis for further research and analysis. In the mediation analysis, we used natural effect models that enable flexible estimation of direct and indirect associations (Lange et al., 2012). However, interpretation of results from this study should consider the following limitations. Firstly, the data collection was cross-sectional, meaning that blood and urine samples for exposure markers, mediators, effect biomarkers, and health outcomes were collected simultaneously. Consequently, the temporal sequence of exposure, mediator, and outcome could not be established. Nevertheless, given the assumption that exposure markers serve as indicators of long-term and ubiquitous exposure, particularly for persistent pollutants, the finding of this study provides baseline evidence for further exploration and more in-depth analysis. Prospective studies are recommended to investigate temporality and causality from exposure, mediators, and health outcomes. Secondly, urine samples were collected on spot, relatively easy but larger variability in predicting urinary exposure markers due to individual differences in kidney function and water consumption (Aylward et al., 2017). Thirdly, health outcomes were measured using self-reported questionnaires on symptoms and medications used, which is prone to recall bias. In future studies, the utilization of physician-diagnosed health outcomes through linkage to routine health records or databases could improve measurement error. Finally, this study did not adjust for some characteristics such as dietary habit, use of products, comorbidities, etc. which might confound the association between pollutants with health outcomes. A comprehensive assessment of lifestyle and other characteristics are recommended in future studies aimed to investigate the causal association of environmental pollutants with asthma and allergic diseases.

## 5 Conclusions

In summary, this study showed that considering simultaneous exposure to pollutants from 10 different chemical groups, MnBP and MBzP were positively associated with FeNO levels in adolescents. Notably, eosinophil count emerged as a significant mediator in the association between phthalates, particularly MnBP and MBzP, and FeNO. Additionally, PCBs and OC pesticides demonstrated an association with eczema, contributing to the existing body of evidence. 8-OHdG, a marker of oxidative stress, seems to mediate the association between certain pesticides and PAHs and allergic rhinitis. Therefore, minimizing exposure to environmental pollutants such as phthalates and pesticides could be helpful to halt the growing burden of asthma and allergic diseases. Monitoring of inflammatory and oxidative stress markers is crucial for understanding of the inflammatory processes in asthma and allergic diseases, which will aid in improving prevention strategies. Furthermore, future prospective studies gathering all relevant individual and environmental characteristics are recommended.

## CRediT authorship contribution statement

**Hamid Y. Hassen:** Writing – review & editing, Writing – original draft, Visualization, Validation, Software, Methodology, Investigation, Formal analysis, Data curation, Conceptualization. **Eva Govarts:** Writing – review & editing, Supervision, Resources, Project administration, Methodology, Investigation, Funding acquisition, Conceptualization. **Sylvie Remy:** Writing – review & editing, Supervision, Software, Resources, Methodology, Investigation, Funding acquisition, Data curation, Conceptualization. **Bianca Cox:** Writing – review & editing, Validation, Supervision, Methodology, Investigation, Conceptualization. **Nina Iszatt:** Writing – review & editing, Validation, Methodology. **Lützen Portengen:** Writing – review & editing, Validation, Methodology, Formal analysis. **Adrian Covaci:** Writing – review & editing. **Greet Schoeters:** Writing – review & editing, Methodology, Funding acquisition. **Elly Den Hond:** Writing – review & editing, Methodology, Funding acquisition. **Stefaan De Henauw:** Writing – review & editing, Methodology, Funding acquisition. **Liesbeth Bruckers:** Writing – review & editing, Methodology, Funding acquisition. **Gudrun Koppen:** Writing – review & editing, Supervision, Project administration, Investigation, Funding acquisition, Conceptualization. **Veerle J. Verheyen:** Writing – review & editing, Supervision, Resources, Project administration, Methodology, Investigation, Funding acquisition, Conceptualization.

## Ethical considerations

Adolescents and one of the parents had to give their signed informed consent. The FLEHS IV study protocol was approved by the Antwerp University Hospital Ethics committee (Belgian registration number B300201732753). A statement was included on the report-back of the individual exposure results to the parents and adolescent, and if preferred to their general practitioner. The medical practitioner of the Provincial Institute of Hygiene (PIH) also intercepted individual questions of respondents on their results afterwards.

## Funding

This work was carried out in the framework of the European Partnership for the Assessment of Risks from Chemicals (PARC) and has received funding from the European Union's 10.13039/100018693Horizon Europe research and innovation program under Grant Agreement No 101057014. Views and opinions expressed are however those of the author(s) only and do not necessarily reflect those of the European Union or the European Health and Digital Executive Agency. Neither the 10.13039/501100000780European Union nor the granting authority can be held responsible for them. The FLEHS IV study was conducted within the framework of the Flemish Center of Expertise on Environment and Health (FLEHS, 2016–2020), funded by the Environment, Nature, and 10.13039/100000015Energy Department of the Flemish government. The views expressed herein are those of the author(s) and are not necessarily endorsed by the Flemish government Analysis of phthalates and per-and polyfluoroalkyl substances were co-funded from the EU 10.13039/501100007601Horizon 2020 Framework Project HBM4EU, Grant Agreement No 733032.

## Declaration of competing interest

The authors declare that they have no known competing financial interests or personal relationships that could have appeared to influence the work reported in this paper.

## Data Availability

The authors do not have permission to share data.
