# Supplementary material for: Association of environmental pollutants with asthma and allergy, and the mediating role of oxidative stress and immune markers in adolescents
Source: Environ Res. 2025 Jan 15;265:120445. doi: 10.1016/j.envres.2024.120445 (PMC11672208; doi:10.1016/j.envres.2024.120445)
Supplement: Multimedia component 1 [file mmc1.pdf]

Supplementary materials: Association of environmental pollutants with asthma and allergy, and the mediating role of oxidative stress and immune markers in adolescents

Hamid Y. Hassen[a*], Eva Govarts[a], Sylvie Remy[a], Bianca Cox[a], Nina Iszatt[b,c], Lützen Portengen[d], Adrian Covaci[e], Greet Schoeters[e,f], Elly Den Hond[g,h], Stefaan De Henauw[i], Liesbeth Bruckers[j], Gudrun Koppen[a¥], Veerle Verheyen[a]

Affiliations

[a] Environmental Intelligence Unit, Flemish Institute for Technological Research (VITO), Boeretang 200, 2400, Mol, Belgium

[b] Division of Climate and Environmental Health, Norwegian Institute of Public Health, Oslo, Norway

[c] Centre for Sustainable Diets, Norwegian Institute of Public Health, Oslo, Norway

[d] Institute for Risk Assessment Sciences, Utrecht University, Utrecht, The Netherlands

[e] Toxicological Center, University of Antwerp, Universiteitsplein 1, 2610 Wilrijk, Belgium

[f] Department of Biomedical Sciences, University of Antwerp, Universiteitsplein 1, 2610 Wilrijk, Belgium

[g] Provincial Institute of Hygiene (PIH), Kronenburgstraa 45, 2000 Antwerpen, Belgium

[h] Family Medicine and Population health, University of Antwerp, Universiteitsplein 1, 2610 Wilrijk, Belgium

[i] Department of Public Health and Primary Care, Ghent university, Corneel Heymanslaan 10, 9000 Ghent, Belgium

[j] BioStat, Data Science Institute, Hasselt University, Martelarenlaan 42, 3500, Hasselt, Belgium

[¥] Deceased Jan 30, 2024

*Correspondence to:

Hamid Y. Hassen, Environmental Intelligence Unit, Flemish Institute for Technological Research (VITO), Boeretang 200, 2400, Mol, Belgium

Hamid.hassen@vito.be

Table of Contents

Table of Contents 2
List of figures 3
List of tables 4
Directed Acyclic Graph (DAG) 5
Quantification of values above LOD/LOQ 6
Questionnaire used to measure health outcomes 9
Correlation matrix 10
Association between exposures and asthma & allergy outcomes and FeNO (biomarker) 12
Association between exposures with mediators 15
Association between mediators with outcomes 17
Mixture analysis 18
References 35

List of figures

Figure S 1. Directed acyclic graph (DAG) illustrating the hypothesized association of environmental pollutants with asthma and allergy, and the mediating role of 8-OHdG and immune markers. 5
Figure S 2. Correlation matrix between ln-transformed exposure biomarkers in the study population. Pearson correlation coefficients are presented. 10
Figure S 3. Correlation matrix between oxidative stress and immune markers in the study population. Pearson correlation coefficients are presented. 11
Figure S 4. Overall effect of the mixture on FeNo estimated by BKMR. 19
Figure S 5. Univariate exposure–response functions of each pollutant on asthma estimated by BKMR. 21
Figure S 6. Overall effect of the mixture on asthma estimated by BKMR. 22
Figure S 7. Individual pollutant effects on asthma estimated by BKMR (the points are point estimates (β) and the bars are 95% CIs). 23
Figure S 8. Univariate exposure–response functions of each pollutant on rhinitis estimated by BKMR. 25
Figure S 9. Overall effect of the mixture on rhinitis estimated by BKMR. 26
Figure S 10. Overall effect of the mixture on eczema estimated by BKMR. 28
Figure S 11. Overall effect of the mixture on skin allergy estimated by BKMR. 30
Figure S 12. Overall effect of the mixture on allergy (any kinds) estimated by BKMR. 32
Figure S 13. Overall effect of the mixture on lower respiratory infection estimated by BKMR. 34

List of tables

Table S 1: LOD/LOQ and percentage of values above the LOD/LOQ for each exposure biomarker 6
Table S 2. LOQ and percentage of values above the LOQ for oxidative stress, immune markers, and FeNO....... 8
Table S 3: Questionnaire used to measure asthma and allergy related outcomes 9
Table S 4. Single pollutant analysis on the associations of IQR increase in pollutants with asthma and allergy health outcomes and FeNO among teenagers. 12
Table S 5. Associations of ln transformed exposure pollutants with mediators (ln transformed) among teenagers. 15
Table S 6. Associations of ln transformed mediators with asthma and allergy health outcomes and FeNO (ln transformed) among teenagers. 17
Table S 7. Associations between exposure biomarker concentrations and FeNO in teenagers estimated by the different statistical methods.. 18
Table S 8. Associations between exposure biomarker concentrations and asthma (last year) in teenagers estimated by the different statistical methods. 20
Table S 9. Associations between exposure biomarker concentrations and rhinitis (ever) in teenagers estimated by the different statistical methods. 24
Table S 10. Associations between exposure biomarker concentrations and eczema (ever) in teenagers estimated by the different statistical methods. 27
Table S 11. Associations between exposure biomarker concentrations and skin allergy in teenagers estimated by the different statistical methods. 29
Table S 12. Associations between exposure biomarker concentrations and all kinds of allergy in teenagers estimated by the different statistical methods. 31
Table S 13. Associations between exposure biomarker concentrations and lower respiratory infection (last year) in teenagers estimated by the different statistical methods. 33

Directed Acyclic Graph (DAG)

- Do environmental pollutants increase the risk of asthma and allergy outcomes?
- Do oxidative stress and immune changes mediate the association between exposure to environmental pollutants and asthma and allergy?

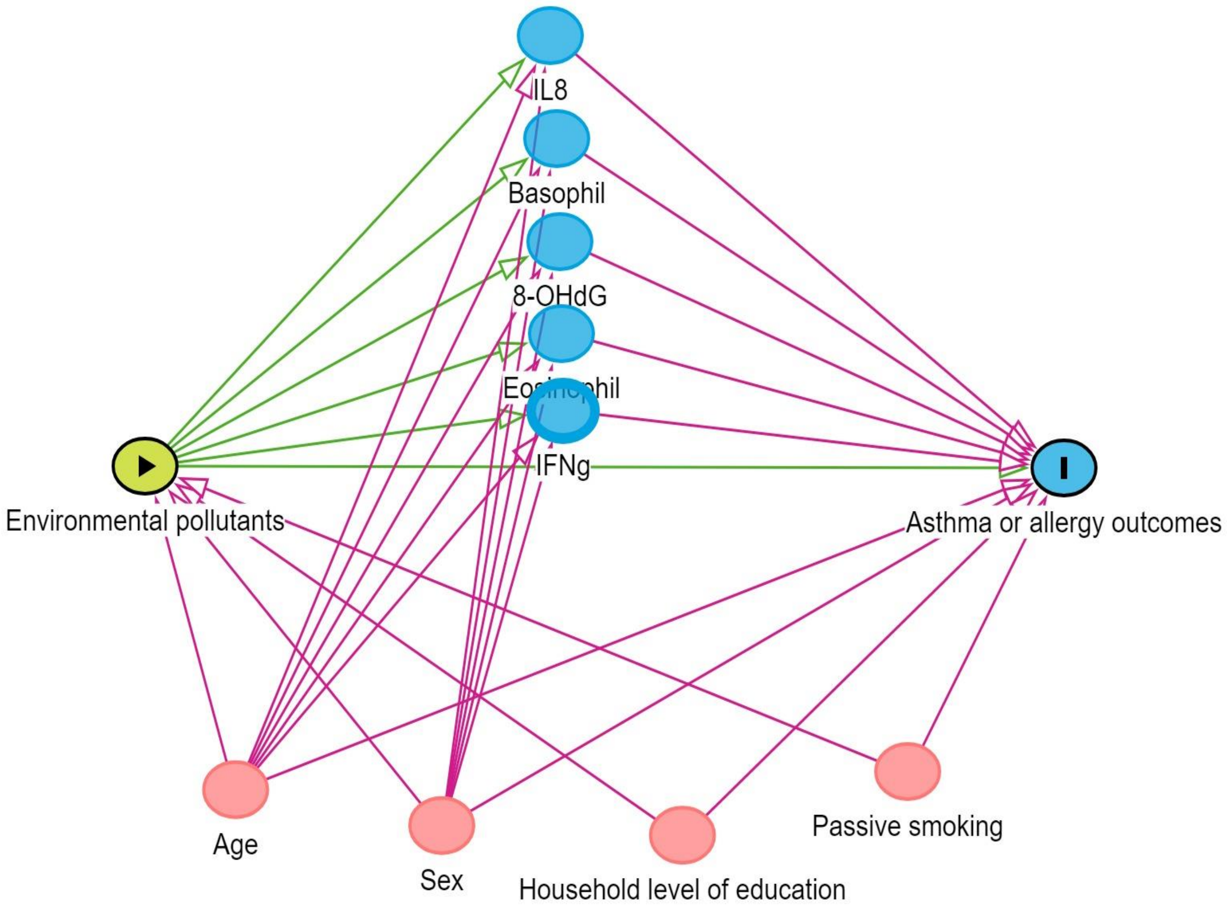

Figure S 1. Directed acyclic graph (DAG) illustrating the hypothesized association of environmental pollutants with asthma and allergy, and the mediating role of 8-OHdG and immune markers.

Quantification of values above LOD/LOQ

Table S 1: LOD/LOQ and percentage of values above the LOD/LOQ for each exposure biomarker

Substance group	Sample	Exposure biomarker	Analytical method	LOD/LOQ	% above LOQ
Pesticide (µg/L)	urine	3-Phenoxybenzoic acid (3-PBA)	HPLC-TQMS (Andersen, Dalsager et al. 2021)	LOD=0.03	99.51
		2,4-Dichlorophenoxyacetic acid (2,4-D)		LOD=0.03	95.84
		3,5,6-Trichloro-2-pyridinol (TCP_Y)		LOD=0.3	98.53
		Glyphosate (Gly)	GC-MS/MS (Lemke, Murawski et al. 2021)	LOQ=0.1	42.05
		Aminomethylphosphonic acid (AMPA)		LOQ=0.1	55.50
Metals (µg/L)	Blood	Cadmium (Cd)	HR-ICP-MS (Schroijen, Baeyens et al. 2008)	LOD=0.007	100.00
		Thallium (Tl)		LOD=0.651	100.00
		Lead (Pb)		LOD=0.048	100.00
		Manganese (Mn)		LOD=0.120	100.00
		Copper (Cu)		LOD=0.551	100.00
	Urine	Cadmium (Cd)	HR-ICP-MS (Schroijen, Baeyens et al. 2008)	LOD=0.010	100.00
		Thallium (Tl)		LOD=0.002	100.00
		Arsenobetaïne (AsB)	UPLC-MS/MS (De Craemer, Croes et al. 2017)	LOD=0.1	91.69
		Arsene(III) (As III)		LOD=0.1	85.33
		Arsene(V) (As V)		LOD=0.1	64.30
		Mono methyl arsenate (MMA)		LOD=0.1	91.69
		Dimethyl arsenate (DMA)		LOD=0.1	99.76
Polycyclic aromatic hydrocarbons (PAH) (µg/L)	Urine	1-Hydroxypyrene (1-OH PYR)	UPLC-MS/MS (Verheyen, Remy et al. 2021)	LOQ=0.015	97.31
		2-Hydroxynaphthtalene (2-OH NAPH)		LOQ=0.150	100.00
		2-Hydroxyphenantrene (2-OH PHE)		LOQ=0.015	97.31
		3-Hydroxyphenantrene (3-OH PHE)		LOQ=0.014	98.53
		4-Hydroxyphenantrene (4-OH PHE)		LOQ=0.014	7.58
Benzene (µg/L)	Urine	T,t'-muco nic acid (t,t'-MA)	HPLC-UV (Schoeters, Govarts et al. 2017)	LOD=2	99.27
Organophosphate flame retardants (OPFRs) (µg/L)	Urine	bis(1-chloro-2-propyl) phosphate (BCIPP)	LC-MS/MS (Bastiaensen, Xu et al. 2018)	LOQ=0.5	9.54
		4-hydroxyphenyl phenyl phosphate (4-OH-DPHP)		LOQ=0.1	14.91
		diphenyl phosphate (DPHP)		LOQ=0.01	99.02
		di-n-butyl phosphate (DNBP)		LOQ=0.05	18.58
		bis(1,3-dichloro-2-propyl) phosphate (BDCIPP)		LOQ=0.01	80.93
		bis(2-butoxyethyl) phosphate (BBOEP)		LOQ=0.04	11.74
		1-hydroxy-2-propyl bis(1-chloro-2-propyl) phosphate (BCIPHIPP)		LOQ=1	95.11
		tris(chloroethyl) phosphate (TCEP)		LOQ=0.04	29.58
		2-ethylhexyl phenyl phosphate (EHPHP)		LOQ=0.05	99.02
		2-hydroxyethyl bis(2-butoxyethyl) phosphate (BBOEHEP)		LOQ=0.005	95.35
		bis(2-butoxyethyl) 3'-hydroxy-2-butoxyethyl phosphate (3-OH-TBOEP)		LOQ=0.01	7.33
		hydroxyphenyl diphenyl phosphate (OH-TPHP)		LOQ=0.05	7.09
		2-ethyl-5-hydroxyhexyl diphenyl phosphate (5-OH-EHDPHP)		LOQ=0.15	98.04
Polychlorinated biphenyls (PCBs) (ng/g lipid)	Blood serum	PCB101	GC-ECNI/MS (Covaci and Voorspoels 2005)	LOQ=4.00	2.93
		PCB99		LOQ=4.00	2.93
		PCB105		LOQ=2.00	13.20
		PCB118		LOQ=2.00	99.76
		PCB153		LOQ=2.00	100.00
		PCB138		LOQ=2.00	100.00
		PCB187		LOQ=2.00	88.51
		PCB183		LOQ=2.00	68.95
		PCB180		LOQ=2.00	100.00
		PCB170		LOQ=2.00	99.76
Chlorinated pesticides (ng/g lipid)	Blood serum	Oxychlordane	GC-ECNI/MS (Covaci and Voorspoels 2005)	LOQ=2	95.35
		Trans-nonachlor		LOQ=2	85.09
		p,p'-DDE		LOQ=4	100.00

		p,p'-DDT		LOQ=4	76.04
		Hexachlorobenzene		LOQ=2	100.00
		Beta hexachlororcyclohexane		LOQ=2	95.84
		Gamma hexachlororcyclohexane		LOQ=2	18.83
Brominated Flame retardants (ng/g lipid)	Blood serum	BDE28	GC-ECNI/MS (Covaci and Voorspoels 2005)	LOQ= 1	3.18
		BDE47		LOQ= 1	55.01
		BDE100		LOQ= 1	3.67
		BDE99		LOQ= 1	42.30
		BDE154		LOQ= 2	38.88
		BDE153		LOQ= 2	23.96
		BDE183		LOQ= 2	2.93
Bisphenol (µg/L)	Urine	Bisphenol A (BPA)	GC-MS/MS (Gys, Ait Bamai et al. 2020)	LOQ=0.3	85.33
		Bisphenol F (BPF)		LOQ=0.02	96.58
		Bisphenol S (BPS)		LOQ=0.04	82.89
		Bisphenol B (BPB)		LOQ=0.02	56.72
		Bisphenol Z (BPZ)		LOQ=0.03	37.41
		Bisphenol AF (BPAF)		LOQ=0.02	13.20
PFAS (µg/L)	Blood serum	perfluoroplonganoic acid (PFPeA)	UPLC-MS/MS (Schoeters, Verheyen et al. 2022)	LOQ= 0.2	4.40
		perfluorohexanoic acid (PFHxA)		LOQ= 0.2	9.78
		perfluoroheptanoic acid (PFHpA)		LOQ= 0.2	5.62
		perfluorooctnoic acid (PFOA)		LOQ= 0.2	100.00
		perfluorononnoic acid (PFNA)		LOQ= 0.2	83.13
		perfluorodecnoic acid (PFDA)		LOQ= 0.2	44.99
		perfluoroundecaanoic acid (PFUnDA)		LOQ= 0.2	11.98
		perfluorododecaanoic acid (PFDoDA)		LOQ= 0.2	5.62
		perfluorobutaansulnoic acid (PFBS)		LOQ= 0.2	4.65
		perfluorohexaansulfnoic acid (PFHxS)		LOQ= 0.2	96.58
		perfluoroheptaansulfnoic acid (PFHpS)		LOQ= 0.2	6.85
		perfluorooctaansulnoic acid (PFOS)		LOQ= 0.2	100.00
Phthalates, DINCH & alternative plasticizers (µg/L)	Urine	Monoethyl phthalate (MEP)	LC-MS/MS (Bastiaensen, Gys et al. 2021)	LOQ= 0.5	100.00
		mono(2-ethyl-5-carboxy- pentyl) phthalate (5cx-MEPP)		LOQ= 0.5	100.00
		mono-2-ethyl-5-hydroxyhexyl phthalate (5OH-MEHP)		LOQ= 0.5	99.76
		mono-isobutyl phthalate (MiBP)		LOQ=0.2	100.00
		mono-n-butyl phthalate (MnBP)		LOQ= 0.5	100.00
		mono-2-ethyl-5-oxohexyl phthalate (5oxo-MEHP)		LOQ=0.2	99.51
		mono-benzyl phthalate (MBzP)		LOQ=0.2	98.29
		mono-2-ethylhexyl phthalate (MEHP)		LOQ= 0.5	84.35
		mono(2-ethyl-5-hydroxyhexyl) adipate (OHMEHA)		LOQ= 0.5	22.98
		mono(2-ethyl-5-hydroxyhexyl) terephthalate (OHMEHTP)		LOQ=0.2	88.51
		mono(7-hydroxy-isononyl) phthalate (MHNP)		LOQ=0.2	100.00
		mono(7-carboxy-isononyl) phthalate (MCOP)		LOQ=0.2	99.51
		cyclohexane-1,2-dicarboxylic mono hydroxyisononyl ester (MHNCH)		LOQ=0.2	95.60
		cyclohexane-1,2-dicarboxylic mono carboxyisooctyl ester (MCOCH)		LOQ=0.2	98.53
		mono(6-hydroxy-isodecyl) phthalate (OHMIDP)		LOQ=0.2	91.69
		mono(6-carboxy-isodecyl) phthalate (CXMIDP)		LOQ=0.2	100.00
		mono(6-oxo-isodecyl) phthalate (OXOMIDP)		LOQ=0.2	78.24
		mono(2-ethylhexyl) adipate (MEHA)		LOQ=0.2	7.09
		mono(2-ethylhexyl) terephthalate (MEHTP)		LOQ=0.2	3.67
		cyclohexane-1,2-dicarboxylic mono isononyl ester (MINCH)		LOQ=0.2	8.80
		di(2-ethylhexyl) trimellitate (SDEHTM)		LOQ=0.2	3.18

Table S 2. LOQ and percentage of values above the LOQ for oxidative stress, immune markers, and FeNO

Mediator	LOQ	% above LOQ
8-OHdG (µg/L)	0.50	99.02
Total basophil (n/µL)		100
Total eosinophil (n/µL)		100
IL6 (pg/mL)	0.06	86.31
IL8 (pg/mL)	0.02	100.00
IFN_{γ} (pg/mL)	0.19	100.00
IL4 (pg/mL)	0.01	29.34
IL2 (pg/mL)	0.03	29.10
IL13 (pg/mL)	0.23	17.85
IL12p70 (pg/mL)	0.04	26.89
**Effect biomarker**		
FeNO (ppb)	-1.00	86.55

Questionnaire used to measure health outcomes

Table S 3: Questionnaire used to measure asthma and allergy related outcomes

Health outcomes	Response options	Algorithm
**Asthma (last year)**		
1. At any time in the last 12 months, have you woken up with a feeling of tightness in your chest?	0. No<br>1. Yes	If Q1=Yes OR Q2 =Yes OR Q3 =Yes OR Q4 =Yes
2. Have you woken up with shortness of breath at any time in the last 12 months?	0. No<br>1. Yes	
3. Have you had an attack of asthma in the last 12 months?	0. No<br>1. Yes	
4. Have you taken medicines for asthma in the last 12 months?	0. No<br>1. Yes	
**Rhinitis (ever)**		
1. Do you have any form of nasal allergy, including hay fever/rhinitis?	0. No<br>1. Yes	If Q1=Yes OR Q2 =Yes OR Q3 =Yes
2. Have you suffered from any form of nasal allergy, including hay fever/rhinitis, in the last 12 months?	0. No<br>1. Yes	
3. Have you taken medicines for hay fever/rhinitis or nasal allergy in the last 12 months? (e.g. nasal sprays, tablets, drops, etc.)	0. No<br>1. Yes	
**Eczema (ever)**		
1. Do you suffer from eczema?	0. No<br>1. Yes	If Q1=Yes
**Skin allergy (last 5 years)**		
1. Have you had any form of allergy or skin rash in the last 5 years after contact with metal (piercing, earring, metal jewellery, etc.)?	0. No<br>1. Yes	If Q1=Yes OR Q2 =Yes OR Q3 =Yes
2. Have you had any form of allergy or skin rash in the last 5 years after contact with care products (make-up, creams, perfume, etc.)?	0. No<br>1. Yes	
3. Have you had any form of allergy or skin rash in the last 5 years after contact with household and maintenance products (dishwashing liquid, washing powder, fabric softener, soap, etc.)	0. No<br>1. Yes	
**Any kind of allergy (last 5 years)**		
1. Have you had any form of allergy or skin rash in the last 5 years after contact with foodstuffs (peanuts, chocolate, strawberries, tomatoes, ...)?	0. No<br>1. Yes	If yes to any of Q1, Q2, Q3, Q4, Q5, or Q6
2. Have you had any form of allergy or skin rash in the last 5 years after contact with medicines?	0. No<br>1. Yes	
3. Have you had any form of allergy or skin rash in the last 5 years after contact with insect bites (strong swelling at bites)?	0. No<br>1. Yes	
4. Have you had any form of allergy or skin rash in the last 5 years after contact with metal (piercing, earring, metal jewellery, etc.)?	0. No<br>1. Yes	
5. Have you had any form of allergy or skin rash in the last 5 years after contact with care products (make-up, creams, perfume, etc.)?	0. No<br>1. Yes	
6. Have you had any form of allergy or skin rash in the last 5 years after contact with household and maintenance products (dishwashing liquid, washing powder, fabric softener, soap, etc.)	0. No<br>1. Yes	
**Lower respiratory infection (last year)**		
Has your son/daughter had one of the following infections in the last year?		If Q1=Yes OR Q2 =Yes
1. Bronchitis	0. No<br>1. Yes	
2. Pneumonia	0. No<br>1. Yes	

Correlation matrix

Figure S 2. Correlation matrix between ln-transformed exposure biomarkers in the study population. Pearson correlation coefficients are presented.

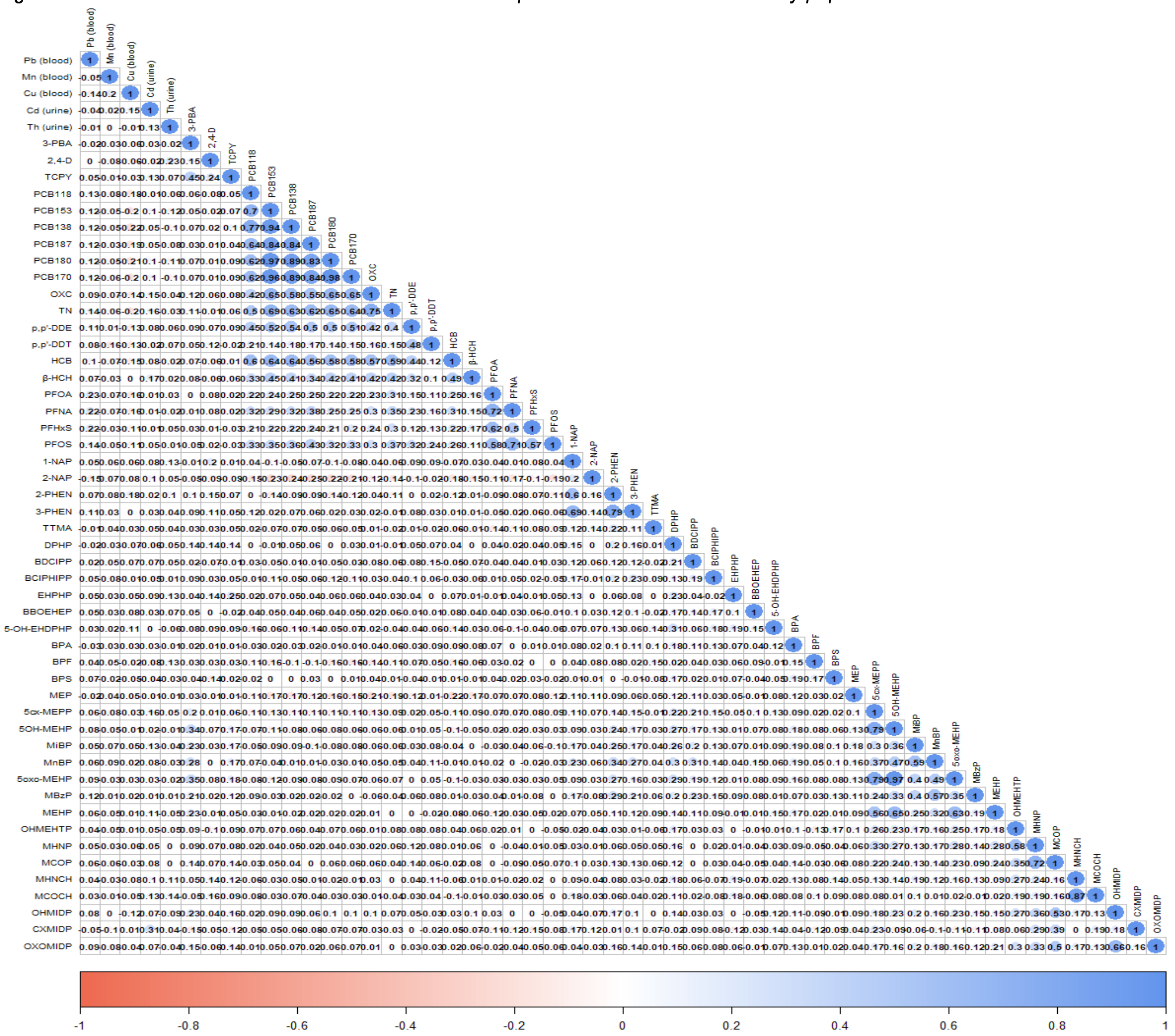

Figure S 3. Correlation matrix between oxidative stress and immune markers in the study population. Pearson correlation coefficients are presented.

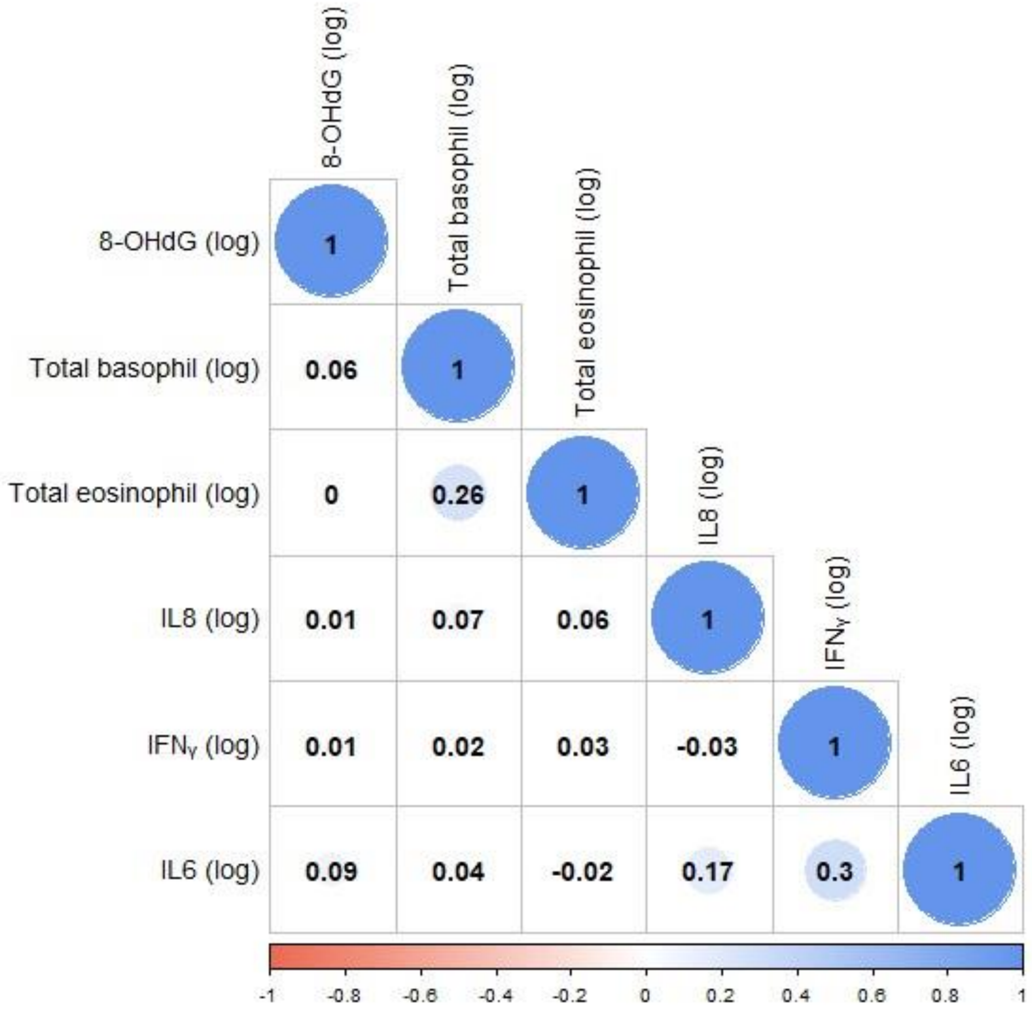

Association between exposures and asthma & allergy outcomes and FeNO (biomarker)

Table S 4. Single pollutant analysis on the associations of IQR increase in pollutants with asthma and allergy health outcomes and FeNO among teenagers.

Group	Exposure	Asthma (last year)		Rhinitis (ever)		Eczema (ever)		Skin allergy (last 5 year)		Any allergy (last 5 year)		Lower respiratory infection (last year)		FeNO	
		OR (95%CI)	p value	OR (95%CI)	p value	OR (95%CI)	p value	OR (95%CI)	p value	OR (95%CI)	p value	OR (95%CI)	p value	percentage change (decimal) per IQR (95%CI)	p value
Metals (µg/L)	Pb (blood)	0.94 (0.65, 1.36)	0.754	0.87 (0.65, 1.16)	0.356	0.93 (0.69, 1.26)	0.662	0.87 (0.62, 1.22)	0.429	0.81 (0.60, 1.08)	0.158	0.77 (0.50, 1.16)	0.215	-0.04 (-0.14, 0.07)	0.494
	Mn (blood)	1.20 (0.83, 1.74)	0.342	1.07 (0.80, 1.44)	0.630	0.90 (0.66, 1.23)	0.513	0.90 (0.64, 1.27)	0.552	1.03 (0.77, 1.39)	0.828	0.89 (0.58, 1.36)	0.593	-0.01 (-0.12, 0.10)	0.807
	Cu (blood)	1.28 (0.96, 1.70)	0.083	1.15 (0.91, 1.45)	0.250	0.99 (0.76, 1.27)	0.920	1.01 (0.79, 1.29)	0.929	0.98 (0.77, 1.23)	0.839	1.05 (0.73, 1.47)	0.790	0.06 (-0.03, 0.16)	0.197
	Cd (urine)	0.91 (0.62, 1.33)	0.623	1.04 (0.77, 1.39)	0.817	1.04 (0.76, 1.43)	0.784	0.77 (0.53, 1.09)	0.143	0.89 (0.66, 1.21)	0.463	1.03 (0.67, 1.57)	0.892	0.01 (-0.09, 0.13)	0.812
	Th (urine)	1.20 (0.80, 1.82)	0.379	**0.67 (0.48, 0.92)**	**0.015**	0.90 (0.64, 1.26)	0.528	0.82 (0.56, 1.19)	0.299	1.09 (0.79, 1.51)	0.608	0.89 (0.56, 1.40)	0.605	-0.07 (-0.18, 0.05)	0.234
Pesticide (µg/L)	3-PBA	1.04 (0.73, 1.45)	0.842	0.96 (0.73, 1.25)	0.757	1.05 (0.79, 1.38)	0.740	1.13 (0.83, 1.54)	0.434	1.09 (0.83, 1.43)	0.532	0.72 (0.48, 1.06)	0.107	-0.09 (-0.18, 0.01)	0.065
	2,4-D	0.77 (0.52, 1.12)	0.166	0.82 (0.61, 1.11)	0.197	0.90 (0.66, 1.23)	0.500	0.92 (0.65, 1.32)	0.661	1.00 (0.74, 1.36)	0.987	1.04 (0.68, 1.62)	0.848	0.01 (-0.10, 0.13)	0.902
	TCPY	1.11 (0.77, 1.63)	0.584	0.89 (0.67, 1.19)	0.430	0.84 (0.62, 1.14)	0.268	**0.66 (0.46, 0.93)**	**0.019**	0.90 (0.67, 1.20)	0.476	0.74 (0.49, 1.12)	0.153	0.02 (-0.09, 0.13)	0.749
PCBs (ng/g lipid)	PCB118	**0.58 (0.38, 0.88)**	**0.013**	0.94 (0.68, 1.28)	0.694	1.09 (0.78, 1.52)	0.602	1.19 (0.84, 1.70)	0.316	1.07 (0.79, 1.46)	0.663	1.05 (0.67, 1.61)	0.833	0.02 (-0.09, 0.15)	0.737
	PCB153	**0.58 (0.36, 0.93)**	**0.026**	1.07 (0.75, 1.53)	0.707	**1.54 (1.05, 2.27)**	**0.027**	1.27 (0.85, 1.90)	0.254	1.12 (0.79, 1.60)	0.512	0.98 (0.59, 1.62)	0.933	0.05 (-0.09, 0.20)	0.501
	PCB138	**0.56 (0.34, 0.88)**	**0.013**	0.97 (0.69, 1.38)	0.881	1.44 (0.99, 2.09)	0.055	1.27 (0.86, 1.89)	0.223	1.03 (0.73, 1.45)	0.872	0.93 (0.57, 1.50)	0.771	0.08 (-0.05, 0.24)	0.230
	PCB187	0.77 (0.54, 1.11)	0.159	0.98 (0.73, 1.32)	0.887	1.24 (0.90, 1.74)	0.199	1.24 (0.88, 1.76)	0.225	0.98 (0.73, 1.32)	0.905	1.10 (0.73, 1.70)	0.644	0.05 (-0.06, 0.18)	0.387
	PCB180	0.67 (0.42, 1.06)	0.095	1.16 (0.82, 1.65)	0.396	**1.52 (1.04, 2.21)**	**0.029**	1.30 (0.87, 1.94)	0.195	1.17 (0.83, 1.65)	0.373	1.07 (0.65, 1.76)	0.784	0.05 (-0.08, 0.20)	0.458
	PCB170	0.67 (0.42, 1.06)	0.091	1.20 (0.85, 1.70)	0.290	**1.50 (1.03, 2.18)**	**0.033**	1.25 (0.84, 1.86)	0.277	1.10 (0.79, 1.56)	0.568	1.10 (0.66, 1.80)	0.707	0.06 (-0.07, 0.21)	0.408
OC pesticides (ng/g lipid)	OXC	0.95 (0.65, 1.36)	0.768	0.97 (0.72, 1.29)	0.837	**1.40 (1.03, 1.92)**	**0.034**	1.03 (0.72, 1.46)	0.881	1.11 (0.82, 1.50)	0.499	0.93 (0.60, 1.42)	0.733	-0.03 (-0.13, 0.09)	0.628
	TN	0.81 (0.56,	0.268	1.12 (0.83,	0.463	**1.52 (1.10,**	**0.012**	1.10 (0.79,	0.585	1.10 (0.82,	0.510	1.21 (0.79,	0.391	-0.06 (-0.16,	0.264

		1.17)		1.50)		**2.12)**		1.54)		1.48)		1.86)		0.05)	
	p,p'-DDE	0.74 (0.49, 1.08)	0.139	0.83 (0.61, 1.11)	0.210	1.12 (0.83, 1.51)	0.451	1.06 (0.76, 1.46)	0.736	0.99 (0.74, 1.31)	0.922	0.72 (0.45, 1.10)	0.147	0.04 (-0.06, 0.17)	0.443
	p,p'-DDT	0.86 (0.64, 1.14)	0.283	0.79 (0.63, 1.00)	0.047	1.08 (0.84, 1.40)	0.575	1.04 (0.79, 1.40)	0.776	1.07 (0.84, 1.36)	0.582	1.01 (0.73, 1.44)	0.948	-0.03 (-0.11, 0.06)	0.533
	HCB	0.67 (0.43, 1.02)	0.067	0.94 (0.67, 1.31)	0.715	0.96 (0.67, 1.36)	0.816	0.72 (0.48, 1.07)	0.109	0.78 (0.55, 1.08)	0.135	0.66 (0.41, 1.05)	0.086	0.03 (-0.09, 0.17)	0.654
	β-HCH	0.81 (0.59, 1.09)	0.171	1.11 (0.89, 1.39)	0.358	0.93 (0.73, 1.19)	0.587	0.99 (0.77, 1.26)	0.929	0.96 (0.76, 1.19)	0.697	0.95 (0.69, 1.30)	0.762	0.01 (-0.07, 0.10)	0.789
PFAS (µg/L)	PFOA	0.89 (0.61, 1.29)	0.539	1.09 (0.82, 1.46)	0.554	0.99 (0.72, 1.34)	0.933	0.87 (0.63, 1.20)	0.384	0.80 (0.60, 1.07)	0.137	1.43 (0.94, 2.20)	0.098	-0.02 (-0.12, 0.10)	0.754
	PFNA	0.89 (0.62, 1.28)	0.526	1.00 (0.75, 1.32)	0.974	1.11 (0.82, 1.51)	0.504	1.04 (0.76, 1.42)	0.825	0.89 (0.67, 1.17)	0.404	**1.66 (1.09, 2.58)**	**0.022**	-0.01 (-0.11, 0.10)	0.791
	PFHxS	0.86 (0.62, 1.18)	0.359	0.94 (0.73, 1.20)	0.600	1.15 (0.89, 1.49)	0.275	0.87 (0.65, 1.14)	0.323	0.81 (0.63, 1.04)	0.103	1.19 (0.85, 1.65)	0.302	-0.06 (-0.14, 0.04)	0.221
	PFOS	0.76 (0.51, 1.12)	0.173	0.92 (0.69, 1.24)	0.600	1.21 (0.89, 1.65)	0.218	1.03 (0.75, 1.42)	0.845	0.92 (0.69, 1.22)	0.573	1.47 (0.99, 2.20)	0.055	-0.01 (-0.11, 0.11)	0.877
PAH (µg/L)	1-NAP	0.91 (0.64, 1.28)	0.577	0.86 (0.65, 1.13)	0.282	0.84 (0.62, 1.12)	0.227	1.05 (0.75, 1.48)	0.767	1.08 (0.81, 1.44)	0.612	1.12 (0.77, 1.63)	0.537	0.01 (-0.08, 0.12)	0.776
	2-NAP	1.03 (0.68, 1.55)	0.872	1.38 (1.00, 1.91)	0.052	1.35 (0.96, 1.90)	0.082	0.77 (0.52, 1.13)	0.185	0.99 (0.72, 1.38)	0.970	1.46 (0.93, 2.27)	0.098	0.03 (-0.09, 0.16)	0.689
	2-PHEN	1.04 (0.71, 1.52)	0.822	1.00 (0.74, 1.33)	0.974	0.92 (0.67, 1.26)	0.606	1.07 (0.75, 1.54)	0.707	1.11 (0.81, 1.51)	0.522	1.08 (0.72, 1.62)	0.699	0.03 (-0.08, 0.15)	0.585
	3-PHEN	0.96 (0.64, 1.39)	0.824	0.93 (0.69, 1.24)	0.625	0.92 (0.67, 1.25)	0.607	1.10 (0.77, 1.58)	0.589	1.03 (0.74, 1.41)	0.864	1.12 (0.75, 1.64)	0.577	0.03 (-0.08, 0.15)	0.626
TTMA (µg/L)	TTMA	0.95 (0.71, 1.27)	0.705	0.96 (0.77, 1.22)	0.757	0.90 (0.71, 1.15)	0.386	0.99 (0.77, 1.29)	0.930	1.00 (0.79, 1.25)	0.974	0.85 (0.61, 1.19)	0.331	0.04 (-0.05, 0.13)	0.407
OPFRs (µg/L)	DPHP	0.90 (0.61, 1.30)	0.578	0.95 (0.71, 1.27)	0.737	0.92 (0.67, 1.25)	0.595	0.89 (0.63, 1.25)	0.514	0.88 (0.65, 1.18)	0.384	1.10 (0.73, 1.64)	0.658	0.04 (-0.06, 0.17)	0.443
	BDCIPP	1.02 (0.75, 1.39)	0.908	0.92 (0.72, 1.17)	0.478	1.07 (0.82, 1.39)	0.624	0.88 (0.66, 1.17)	0.371	0.81 (0.62, 1.04)	0.095	0.93 (0.66, 1.31)	0.674	0.05 (-0.04, 0.15)	0.311
	BCIPHIPP	1.36 (0.96, 1.93)	0.089	0.87 (0.66, 1.14)	0.313	1.01 (0.76, 1.35)	0.932	1.35 (0.99, 1.85)	0.063	1.02 (0.78, 1.34)	0.882	1.21 (0.82, 1.80)	0.339	-0.02 (-0.12, 0.08)	0.663
	EHPHP	1.00 (0.72, 1.45)	0.986	0.80 (0.60, 1.05)	0.113	0.85 (0.63, 1.15)	0.276	0.71 (0.48, 1.02)	0.066	0.82 (0.59, 1.12)	0.204	0.87 (0.62, 1.26)	0.440	0.05 (-0.06, 0.17)	0.380
	BBOEHEP	1.11 (0.80, 1.54)	0.525	0.91 (0.70, 1.18)	0.487	0.96 (0.73, 1.25)	0.744	0.92 (0.67, 1.23)	0.565	0.81 (0.61, 1.05)	0.116	1.28 (0.89, 1.83)	0.181	-0.04 (-0.13, 0.05)	0.365
	5-OH-EHDPHP	1.03 (0.70, 1.51)	0.879	0.98 (0.73, 1.32)	0.908	1.01 (0.73, 1.39)	0.960	0.97 (0.67, 1.40)	0.858	1.07 (0.79, 1.45)	0.681	0.91 (0.59, 1.38)	0.651	0.06 (-0.05, 0.19)	0.322

Bisphenol (µg/L)	BPA	0.93 (0.68, 1.28)	0.634	0.99 (0.77, 1.27)	0.927	0.94 (0.72, 1.23)	0.640	0.84 (0.63, 1.13)	0.242	0.93 (0.72, 1.20)	0.572	1.43 (1.00, 2.06)	0.055	0.03 (-0.06, 0.13)	0.508
	BPF	1.01 (0.73, 1.39)	0.927	0.77 (0.59, 1.00)	0.052	0.96 (0.73, 1.26)	0.795	1.09 (0.80, 1.49)	0.561	1.12 (0.86, 1.46)	0.411	1.21 (0.86, 1.71)	0.275	-0.02 (-0.11, 0.07)	0.620
	BPS	0.86 (0.66, 1.12)	0.266	0.99 (0.80, 1.23)	0.940	0.93 (0.74, 1.16)	0.510	0.94 (0.72, 1.22)	0.644	1.09 (0.87, 1.37)	0.478	0.88 (0.66, 1.18)	0.401	0.06 (-0.02, 0.15)	0.139
Phthalates, DINCH, and replacements (µg/L)	MEP	1.21 (0.84, 1.72)	0.288	**0.72 (0.52, 0.99)**	0.047	1.16 (0.85, 1.56)	0.331	1.06 (0.76, 1.46)	0.729	1.17 (0.87, 1.56)	0.297	0.88 (0.55, 1.34)	0.565	-0.06 (-0.16, 0.05)	0.241
	5cx-MEPP	0.76 (0.50, 1.15)	0.199	0.88 (0.64, 1.20)	0.422	0.90 (0.65, 1.25)	0.536	0.89 (0.62, 1.27)	0.523	1.09 (0.79, 1.51)	0.613	0.63 (0.40, 0.98)	0.045	-0.05 (-0.16, 0.07)	0.401
	5OH-MEHP	0.83 (0.57, 1.19)	0.310	0.89 (0.67, 1.18)	0.419	0.93 (0.69, 1.25)	0.614	1.01 (0.73, 1.40)	0.934	1.16 (0.87, 1.56)	0.303	0.78 (0.52, 1.15)	0.211	-0.05 (-0.15, 0.05)	0.323
	MiBP	0.94 (0.63, 1.35)	0.729	0.90 (0.67, 1.21)	0.496	0.99 (0.72, 1.33)	0.943	1.01 (0.72, 1.40)	0.951	1.19 (0.89, 1.60)	0.237	0.98 (0.64, 1.46)	0.924	0.08 (-0.03, 0.20)	0.185
	MnBP	1.03 (0.66, 1.59)	0.900	1.20 (0.86, 1.68)	0.291	1.06 (0.74, 1.53)	0.740	1.02 (0.68, 1.52)	0.917	0.99 (0.70, 1.39)	0.954	0.88 (0.54, 1.40)	0.583	**0.20 (0.06, 0.36)**	**0.005**
	5oxo-MEHP	0.81 (0.57, 1.16)	0.243	0.91 (0.69, 1.19)	0.474	0.97 (0.73, 1.30)	0.850	1.03 (0.76, 1.41)	0.853	1.15 (0.88, 1.53)	0.310	0.79 (0.55, 1.15)	0.223	-0.06 (-0.16, 0.04)	0.196
	MBzP	1.00 (0.70, 1.41)	0.989	1.15 (0.88, 1.50)	0.314	0.94 (0.70, 1.26)	0.678	0.99 (0.72, 1.35)	0.927	1.00 (0.77, 1.31)	0.975	0.85 (0.57, 1.24)	0.398	**0.13 (0.02, 0.25)**	**0.015**
	MEHP	0.83 (0.61, 1.14)	0.240	0.93 (0.73, 1.19)	0.566	1.14 (0.88, 1.49)	0.334	1.06 (0.80, 1.41)	0.688	1.19 (0.93, 1.55)	0.168	0.96 (0.69, 1.34)	0.787	-0.03 (-0.12, 0.06)	0.496
	OHMEHTP	0.84 (0.63, 1.12)	0.223	1.07 (0.85, 1.34)	0.567	0.94 (0.74, 1.19)	0.611	**0.75 (0.57, 0.98)**	**0.041**	0.95 (0.76, 1.18)	0.629	0.78 (0.56, 1.08)	0.129	-0.04 (-0.12, 0.05)	0.361
	MHNP	0.69 (0.46, 0.99)	0.056	1.09 (0.85, 1.40)	0.476	0.95 (0.71, 1.24)	0.721	0.83 (0.59, 1.12)	0.244	1.02 (0.80, 1.31)	0.862	0.92 (0.62, 1.32)	0.676	0.00 (-0.09, 0.10)	0.943
	MCOP	0.83 (0.59, 1.13)	0.260	1.03 (0.81, 1.30)	0.805	0.90 (0.68, 1.16)	0.415	0.87 (0.63, 1.17)	0.353	1.03 (0.81, 1.30)	0.836	1.04 (0.74, 1.43)	0.800	0.00 (-0.09, 0.09)	0.949
	MHNCH	0.96 (0.66, 1.36)	0.835	**0.64 (0.46, 0.86)**	**0.004**	0.80 (0.58, 1.07)	0.143	0.94 (0.68, 1.28)	0.692	0.95 (0.72, 1.24)	0.692	1.21 (0.82, 1.73)	0.315	0.01 (-0.08, 0.12)	0.787
	MCOCH	1.03 (0.70, 1.50)	0.885	**0.70 (0.50, 0.95)**	**0.025**	0.72 (0.51, 1.00)	0.054	0.95 (0.66, 1.34)	0.769	1.02 (0.75, 1.37)	0.917	1.18 (0.78, 1.78)	0.426	0.05 (-0.06, 0.17)	0.400
	OHMIDP	1.07 (0.78, 1.48)	0.658	1.03 (0.80, 1.32)	0.805	0.94 (0.71, 1.23)	0.655	0.92 (0.67, 1.25)	0.596	1.02 (0.79, 1.32)	0.885	1.02 (0.72, 1.44)	0.928	0.04 (-0.06, 0.14)	0.446
	CXMIDP	0.87 (0.54, 1.23)	0.489	1.11 (0.85, 1.44)	0.432	0.99 (0.71, 1.30)	0.931	0.89 (0.60, 1.27)	0.541	1.04 (0.79, 1.36)	0.756	0.76 (0.44, 1.17)	0.288	0.03 (-0.07, 0.14)	0.547
	OXOMIDP	1.01 (0.73, 1.39)	0.948	1.03 (0.80, 1.31)	0.836	0.94 (0.72, 1.22)	0.620	0.94 (0.70, 1.26)	0.663	0.91 (0.71, 1.17)	0.466	1.18 (0.83, 1.67)	0.360	0.02 (-0.07, 0.12)	0.696

The model is adjusted for age, sex, household level of education, passive smoking
IQR: interquartile range; OR: odds ratio

Association between exposures with mediators

Table S 5. Associations of ln transformed exposure pollutants with mediators (ln transformed) among teenagers.

Group	Exposure	8-OHdG		Basophil		Eosinophil		IL8		IFN$_{\gamma}$		IL6	
		β (95%CI)	p value	β (95%CI)	p value	β (95%CI)	p value	β (95%CI)	p value	β (95%CI)	p value	β (95%CI)	p value
Metals (µg/L)	Pb (blood)	0.02 (-0.03, 0.07)	0.361	-0.02 (-0.08, 0.05)	0.574	**0.17 (0.07, 0.26)**	**0.000**	0.05 (-0.03, 0.13)	0.193	0.07 (-0.02, 0.16)	0.124	0.01 (-0.09, 0.12)	0.798
	Mn (blood)	-0.05 (-0.10, 0.01)	0.104	0.03 (-0.04, 0.11)	0.347	0.01 (-0.09, 0.12)	0.805	0.00 (-0.09, 0.08)	0.972	0.04 (-0.06, 0.14)	0.468	0.00 (-0.11, 0.11)	0.971
	Cu (blood)	-0.01 (-0.05, 0.04)	0.718	0.03 (-0.02, 0.09)	0.263	-0.01 (-0.09, 0.08)	0.891	**-0.10 (-0.17, -0.04)**	**0.002**	0.01 (-0.07, 0.09)	0.815	**0.10 (0.01, 0.19)**	**0.024**
	Cd (urine)	**0.08 (0.03, 0.13)**	**0.003**	0.03 (-0.05, 0.10)	0.456	-0.09 (-0.19, 0.02)	0.097	**-0.11 (-0.20, -0.03)**	**0.011**	0.03 (-0.07, 0.13)	0.538	0.06 (-0.06, 0.17)	0.341
	Th (urine)	0.05 (0.00, 0.10)	0.061	0.05 (-0.03, 0.13)	0.188	-0.03 (-0.14, 0.08)	0.599	0.04 (-0.05, 0.13)	0.371	0.01 (-0.10, 0.11)	0.869	0.01 (-0.11, 0.13)	0.839
Pesticide (µg/L)	3-PBA	**0.05 (0.01, 0.09)**	**0.027**	0.02 (-0.04, 0.08)	0.545	-0.01 (-0.10, 0.08)	0.842	-0.01 (-0.09, 0.07)	0.831	0.02 (-0.06, 0.11)	0.600	0.02 (-0.08, 0.12)	0.731
	2,4-D	**0.12 (0.07, 0.17)**	**0.000**	0.07 (0.00, 0.14)	0.063	0.01 (-0.09, 0.11)	0.841	0.09 (0.00, 0.17)	0.050	-0.07 (-0.17, 0.02)	0.141	-0.01 (-0.12, 0.10)	0.840
	TCPY	**0.07 (0.02, 0.12)**	**0.003**	0.02 (-0.05, 0.09)	0.584	-0.04 (-0.14, 0.06)	0.434	0.04 (-0.04, 0.13)	0.299	0.01 (-0.09, 0.10)	0.887	0.04 (-0.07, 0.15)	0.506
PCBs (ng/g lipid)	PCB118	-0.02 (-0.08, 0.04)	0.450	**-0.10 (-0.18, -0.03)**	**0.006**	-0.01 (-0.12, 0.10)	0.870	0.06 (-0.03, 0.15)	0.204	-0.02 (-0.12, 0.08)	0.697	-0.06 (-0.17, 0.06)	0.338
	PCB153	0.00 (-0.06, 0.06)	0.985	**-0.11 (-0.19, -0.03)**	**0.010**	0.05 (-0.07, 0.17)	0.379	-0.02 (-0.12, 0.07)	0.620	0.07 (-0.04, 0.18)	0.210	**-0.16 (-0.29, -0.03)**	**0.015**
	PCB138	0.00 (-0.06, 0.06)	0.981	**-0.09 (-0.17, -0.02)**	**0.018**	0.07 (-0.05, 0.18)	0.238	0.04 (-0.05, 0.14)	0.398	0.03 (-0.08, 0.14)	0.619	**-0.15 (-0.27, -0.02)**	**0.021**
	PCB187	0.00 (-0.05, 0.05)	0.993	-0.02 (-0.09, 0.05)	0.525	0.07 (-0.03, 0.17)	0.150	0.02 (-0.06, 0.10)	0.645	0.08 (-0.01, 0.18)	0.085	-0.09 (-0.20, 0.02)	0.109
	PCB180	0.01 (-0.05, 0.07)	0.745	**-0.08 (-0.16, 0.00)**	**0.041**	0.05 (-0.07, 0.17)	0.398	0.00 (-0.10, 0.09)	0.940	0.08 (-0.03, 0.19)	0.157	-0.12 (-0.24, 0.01)	0.067
	PCB170	0.01 (-0.05, 0.08)	0.657	-0.08 (-0.16, 0.00)	0.054	0.07 (-0.04, 0.19)	0.226	0.02 (-0.07, 0.12)	0.640	0.07 (-0.04, 0.18)	0.212	-0.11 (-0.23, 0.02)	0.091
OC pesticides (ng/g lipid)	OXC	0.03 (-0.02, 0.08)	0.288	-0.04 (-0.11, 0.03)	0.229	0.07 (-0.03, 0.16)	0.177	-0.03 (-0.11, 0.05)	0.441	0.10 (0.01, 0.19)	0.035	-0.03 (-0.13, 0.08)	0.595
	TN	0.02 (-0.04, 0.07)	0.551	-0.04 (-0.11, 0.02)	0.205	0.07 (-0.02, 0.17)	0.137	-0.04 (-0.12, 0.04)	0.279	0.07 (-0.02, 0.17)	0.109	-0.07 (-0.17, 0.03)	0.186
	p,p'-DDE	0.01 (-0.04, 0.07)	0.618	**-0.08 (-0.15, -0.01)**	**0.027**	0.05 (-0.05, 0.15)	0.295	0.01 (-0.07, 0.10)	0.736	-0.02 (-0.12, 0.08)	0.691	-0.09 (-0.20, 0.02)	0.114
	p,p'-DDT	0.01 (-0.04, 0.05)	0.737	-0.04 (-0.09, 0.02)	0.182	-0.05 (-0.13, 0.04)	0.268	0.00 (-0.07, 0.07)	0.941	-0.02 (-0.10, 0.05)	0.542	0.02 (-0.07, 0.11)	0.630
	HCB	-0.01 (-0.07, 0.05)	0.648	-0.06 (-0.13, 0.02)	0.137	**0.18 (0.07, 0.29)**	**0.002**	-0.01 (-0.11, 0.09)	0.838	0.07 (-0.04, 0.18)	0.187	-0.04 (-0.17, 0.08)	0.488
	β-HCH	0.00 (-0.04, 0.04)	0.957	**-0.06 (-0.11, 0.00)**	**0.035**	0.06 (-0.02, 0.13)	0.138	**-0.07 (-0.13, 0.00)**	**0.040**	0.04 (-0.03, 0.12)	0.290	0.04 (-0.05, 0.12)	0.400
PFAS (µg/L)	PFOA	0.04 (-0.01, 0.09)	0.130	0.01 (-0.06, 0.08)	0.742	**0.10 (0.00, 0.20)**	**0.040**	**0.13 (0.05, 0.21)**	**0.001**	0.03 (-0.06, 0.12)	0.526	0.01 (-0.09, 0.12)	0.798
	PFNA	0.02 (-0.03, 0.07)	0.503	-0.03 (-0.09, 0.04)	0.451	0.07 (-0.03, 0.16)	0.166	0.08 (0.01, 0.16)	0.036	0.03 (-0.06, 0.12)	0.544	-0.03 (-0.14, 0.07)	0.506
	PFHxS	0.02 (-0.02, 0.06)	0.360	-0.01 (-0.07, 0.04)	0.610	0.06 (-0.03, 0.14)	0.193	0.04 (-0.03, 0.11)	0.220	0.06 (-0.01, 0.14)	0.104	0.02 (-0.07, 0.10)	0.719
	PFOS	0.03 (-0.03, 0.08)	0.321	0.00 (-0.07, 0.07)	0.959	0.08 (-0.02, 0.19)	0.102	0.06 (-0.02, 0.14)	0.157	0.05 (-0.04, 0.15)	0.267	-0.07 (-0.18, 0.03)	0.177
PAH (µg/L)	1-NAP	**0.07 (0.02, 0.11)**	**0.004**	0.00 (-0.06, 0.07)	0.968	-0.03 (-0.12, 0.07)	0.571	0.06 (-0.02, 0.14)	0.138	0.03 (-0.06, 0.12)	0.545	0.09 (-0.01, 0.19)	0.078

	2-NAP	0.03 (-0.03, 0.09)	0.303	0.03 (-0.05, 0.11)	0.500	**-0.17 (-0.28, -0.05)**	**0.004**	0.00 (-0.09, 0.10)	0.973	-0.05 (-0.16, 0.06)	0.344	0.04 (-0.08, 0.17)	0.504
	2-PHEN	**0.07 (0.02, 0.12)**	**0.004**	0.06 (-0.01, 0.13)	0.116	0.04 (-0.06, 0.14)	0.430	0.05 (-0.04, 0.13)	0.280	0.04 (-0.05, 0.13)	0.399	**0.16 (0.06, 0.27)**	**0.003**
	3-PHEN	0.05 (0.00, 0.10)	0.058	0.03 (-0.04, 0.10)	0.388	0.06 (-0.04, 0.16)	0.241	0.04 (-0.04, 0.13)	0.312	0.06 (-0.04, 0.15)	0.244	**0.11 (0.00, 0.22)**	**0.042**
TTMA (µg/L)	TTMA	0.03 (-0.01, 0.07)	0.088	**0.08 (0.03, 0.14)**	**0.003**	0.02 (-0.06, 0.10)	0.644	0.07 (0.00, 0.14)	0.063	0.08 (0.00, 0.16)	0.047	**0.10 (0.01, 0.19)**	**0.024**
OPFRs (µg/L)	DPHP	**0.07 (0.02, 0.12)**	**0.008**	0.07 (0.00, 0.14)	0.056	**0.14 (0.04, 0.24)**	**0.006**	0.03 (-0.06, 0.11)	0.541	0.02 (-0.08, 0.11)	0.722	0.06 (-0.05, 0.17)	0.268
	BDCIPP	-0.01 (-0.05, 0.04)	0.774	0.02 (-0.04, 0.08)	0.441	0.04 (-0.04, 0.12)	0.345	-0.02 (-0.09, 0.05)	0.650	0.03 (-0.05, 0.11)	0.450	0.08 (-0.01, 0.17)	0.095
	BCIPHIPP	-0.01 (-0.05, 0.04)	0.812	-0.01 (-0.07, 0.06)	0.787	**0.12 (0.03, 0.21)**	**0.013**	0.05 (-0.03, 0.13)	0.225	-0.02 (-0.11, 0.07)	0.737	0.02 (-0.08, 0.12)	0.682
	EHPHP	**0.06 (0.01, 0.11)**	**0.013**	-0.01 (-0.07, 0.06)	0.872	0.01 (-0.09, 0.11)	0.819	-0.03 (-0.11, 0.05)	0.430	-0.03 (-0.12, 0.06)	0.551	-0.03 (-0.14, 0.07)	0.555
	BBOEHEP	0.01 (-0.04, 0.05)	0.713	0.04 (-0.02, 0.10)	0.225	0.06 (-0.03, 0.15)	0.198	-0.05 (-0.13, 0.02)	0.178	0.00 (-0.08, 0.09)	0.950	-0.02 (-0.11, 0.08)	0.738
	5-OH-EHDPHP	0.04 (-0.01, 0.10)	0.091	**0.08 (0.01, 0.16)**	**0.024**	**0.18 (0.07, 0.28)**	**0.001**	-0.04 (-0.14, 0.05)	0.329	**0.12 (0.02, 0.23)**	**0.020**	0.10 (-0.01, 0.22)	0.081
Bisphenol (µg/L)	BPA	**0.05 (0.01, 0.10)**	**0.011**	0.03 (-0.03, 0.09)	0.385	0.04 (-0.04, 0.13)	0.306	0.07 (0.00, 0.14)	0.066	-0.06 (-0.14, 0.03)	0.190	0.00 (-0.10, 0.09)	0.951
	BPF	0.04 (-0.01, 0.08)	0.105	0.03 (-0.03, 0.09)	0.346	0.01 (-0.07, 0.10)	0.743	0.03 (-0.04, 0.11)	0.359	0.00 (-0.08, 0.09)	0.918	0.06 (-0.03, 0.16)	0.186
	BPS	-0.01 (-0.05, 0.03)	0.529	0.04 (-0.02, 0.09)	0.166	0.05 (-0.03, 0.12)	0.194	0.03 (-0.03, 0.09)	0.343	-0.06 (-0.13, 0.01)	0.089	0.00 (-0.08, 0.08)	0.985
Phthalates, DINCH and replacement (µg/L)	MEP	-0.02 (-0.07, 0.03)	0.459	**0.07 (0.00, 0.14)**	**0.039**	-0.06 (-0.16, 0.04)	0.221	-0.01 (-0.09, 0.07)	0.750	-0.04 (-0.13, 0.06)	0.431	0.00 (-0.11, 0.10)	0.988
	5cx-MEPP	**0.06 (0.00, 0.11)**	**0.038**	0.01 (-0.06, 0.09)	0.746	0.00 (-0.11, 0.12)	0.931	0.09 (0.00, 0.19)	0.050	0.04 (-0.07, 0.15)	0.456	0.06 (-0.06, 0.18)	0.362
	5OH-MEHP	0.04 (-0.01, 0.09)	0.099	0.02 (-0.05, 0.09)	0.520	0.01 (-0.09, 0.11)	0.873	**0.09 (0.00, 0.17)**	**0.042**	0.02 (-0.07, 0.12)	0.672	0.06 (-0.05, 0.16)	0.305
	MiBP	0.03 (-0.02, 0.08)	0.218	0.02 (-0.05, 0.09)	0.561	0.09 (-0.01, 0.19)	0.078	0.00 (-0.08, 0.09)	0.913	0.03 (-0.06, 0.13)	0.483	0.00 (-0.11, 0.11)	0.995
	MnBP	0.05 (-0.01, 0.10)	0.116	0.04 (-0.04, 0.13)	0.313	**0.13 (0.01, 0.25)**	**0.031**	0.03 (-0.07, 0.13)	0.604	0.06 (-0.06, 0.17)	0.332	0.11 (-0.02, 0.24)	0.098
	5oxo-MEHP	0.04 (0.00, 0.09)	0.067	0.03 (-0.04, 0.09)	0.452	0.00 (-0.09, 0.10)	0.935	**0.09 (0.01, 0.17)**	**0.034**	0.03 (-0.06, 0.12)	0.526	0.09 (-0.02, 0.19)	0.100
	MBzP	0.02 (-0.03, 0.07)	0.412	0.01 (-0.06, 0.07)	0.874	**0.12 (0.03, 0.21)**	**0.013**	0.01 (-0.07, 0.10)	0.726	**0.11 (0.02, 0.20)**	**0.019**	0.05 (-0.05, 0.16)	0.335
	MEHP	-0.03 (-0.07, 0.01)	0.134	0.06 (0.00, 0.12)	0.064	0.00 (-0.09, 0.08)	0.956	0.01 (-0.06, 0.09)	0.715	**0.09 (0.00, 0.17)**	**0.042**	0.05 (-0.04, 0.14)	0.300
	OHMEHTP	-0.02 (-0.06, 0.02)	0.409	-0.02 (-0.07, 0.04)	0.581	0.00 (-0.08, 0.08)	0.985	-0.03 (-0.10, 0.03)	0.315	0.00 (-0.08, 0.08)	0.992	0.08 (-0.01, 0.17)	0.075
	MHNP	0.02 (-0.02, 0.07)	0.272	-0.02 (-0.08, 0.05)	0.620	-0.06 (-0.15, 0.03)	0.228	-0.01 (-0.09, 0.06)	0.744	0.01 (-0.08, 0.10)	0.816	0.07 (-0.02, 0.17)	0.136
	MCOP	0.03 (-0.01, 0.07)	0.112	0.00 (-0.06, 0.06)	0.932	**-0.10 (-0.19, -0.02)**	**0.017**	-0.01 (-0.08, 0.07)	0.865	-0.01 (-0.09, 0.07)	0.858	0.02 (-0.07, 0.11)	0.648
	MHNCH	0.04 (-0.01, 0.09)	0.108	0.02 (-0.05, 0.09)	0.579	0.03 (-0.06, 0.13)	0.521	-0.02 (-0.10, 0.06)	0.644	0.01 (-0.09, 0.10)	0.903	0.02 (-0.09, 0.12)	0.767
	MCOCH	0.04 (-0.01, 0.10)	0.084	0.04 (-0.03, 0.11)	0.283	0.04 (-0.06, 0.14)	0.454	-0.02 (-0.11, 0.07)	0.651	-0.02 (-0.12, 0.08)	0.750	0.00 (-0.11, 0.12)	0.934
	OHMIDP	**0.04 (0.00, 0.09)**	**0.047**	0.03 (-0.03, 0.09)	0.359	-0.03 (-0.11, 0.06)	0.554	**0.08 (0.00, 0.15)**	**0.040**	0.01 (-0.07, 0.09)	0.837	-0.01 (-0.11, 0.08)	0.761
	CXMIDP	0.02 (-0.03, 0.07)	0.426	0.05 (-0.02, 0.12)	0.145	-0.07 (-0.17, 0.03)	0.174	0.00 (-0.08, 0.08)	0.968	-0.01 (-0.10, 0.08)	0.826	-0.02 (-0.13, 0.08)	0.674

	OXOMIDP	0.02 (-0.03, 0.06)	0.447	0.03 (-0.04, 0.09)	0.397	-0.02 (-0.11, 0.07)	0.664	-0.01 (-0.09, 0.06)	0.768	0.01 (-0.07, 0.10)	0.774	0.04 (-0.05, 0.14)	0.378

The model is adjusted for age, sex, household level of education, passive smoking

Association between mediators with outcomes

Table S 6. Associations of ln transformed mediators with asthma and allergy health outcomes and FeNO (ln transformed) among teenagers.

Exposure	Asthma (last year)		Rhinitis (ever)		Eczema (ever)		Skin allergy (last 5 year)		Any allergy (last 5 year)		Lower respiratory infection (last year)		FeNO	
	OR (95%CI)	p value	OR (95%CI)	p value	OR (95%CI)	p value	OR (95%CI)	p value	OR (95%CI)	p value	OR (95%CI)	p value	β (95%CI)	p value
8-OHdG (µg/L)	1.18 (0.82, 1.71)	0.390	**1.33 (1.01, 1.80)**	**0.048**	1.25 (0.93, 1.70)	0.154	0.98 (0.71, 1.35)	0.914	1.06 (0.81, 1.39)	0.676	1.00 (0.67, 1.52)	0.982	0.00 (-0.09, 0.10)	0.956
Total basophil (n/µL)	1.26 (0.87, 1.84)	0.227	1.06 (0.80, 1.42)	0.675	1.19 (0.88, 1.64)	0.267	1.06 (0.77, 1.47)	0.732	1.12 (0.85, 1.50)	0.423	1.01 (0.68, 1.53)	0.948	0.04 (-0.07, 0.15)	0.445
Total eosinophil (n/µL)	**1.53 (1.06, 2.23)**	**0.025**	**1.51 (1.12, 2.04)**	**0.007**	1.17 (0.85, 1.62)	0.341	1.30 (0.91, 1.86)	0.144	1.11 (0.82, 1.50)	0.508	1.09 (0.71, 1.65)	0.699	**0.34 (0.23, 0.44)**	**0.000**
IL8 (pg/mL)	0.99 (0.68, 1.41)	0.950	1.04 (0.79, 1.38)	0.765	0.79 (0.56, 1.09)	0.160	**0.50 (0.32, 0.74)**	**0.001**	**0.59 (0.42, 0.81)**	**0.002**	1.11 (0.73, 1.64)	0.613	0.03 (-0.08, 0.14)	0.555
IFN$_{\gamma}$ (pg/mL)	0.91 (0.67, 1.20)	0.529	1.04 (0.84, 1.28)	0.728	0.98 (0.77, 1.23)	0.890	0.97 (0.76, 1.22)	0.797	0.87 (0.70, 1.07)	0.207	0.79 (0.55, 1.09)	0.180	0.07 (-0.02, 0.15)	0.109
IL6	0.83 (0.60, 1.18)	0.297	1.06 (0.81, 1.39)	0.697	0.97 (0.73, 1.30)	0.818	0.78 (0.57 ,1.06)	0.111	0.80 (0.61, 1.06)	0.119	1.17 (0.79, 1.77)	0.460	0.05 (-0.05, 0.16)	0.307

The model is adjusted for age, sex, household level of education, passive smoking

Mixture analysis

FeNO

Table S 7. Associations between exposure biomarker concentrations and FeNO in teenagers estimated by the different statistical methods.

Exposure	BKMR			BMA				ENET	
	% change (95%CrI)	groupPIP	condPIP	% change (95%CrI)	Group PIP	Conditional PIP	PIP	% change	Selection probability
Pb (blood)	-3 (-13, 8)	0.385	0.156	0 (0, 0)	0.019	0.342	0.007	0	0.03
Mn (blood)	-4 (-12, 6)	0.385	0.109	0 (0, 0)	0.019	0.000	0.000	0	0.03
Cu (blood)	8 (-4, 22)	0.385	0.387	0 (0, 0)	0.019	0.474	0.009	0	0.1
Cd (urine)	0 (-10, 11)	0.385	0.146	0 (0, 0)	0.019	0.132	0.003	0	0.01
Th (urine)	-5 (-15, 6)	0.385	0.201	0 (0, 0)	0.019	0.053	0.001	0	0.1
3-PBA	-11 (-23, 3)	0.48	0.635	0 (0, 0)	0.047	0.602	0.028	0	0.28
2,4-D	5 (-7, 18)	0.48	0.181	0 (0, 0)	0.047	0.290	0.014	0	0.04
TCPY	3 (-9, 17)	0.48	0.183	0 (0, 0)	0.047	0.108	0.005	0	0.07
PCB118	-1 (-13, 12)	0.306	0.245	0 (0, 0)	0.016	0.125	0.002	0	0.01
PCB187	8 (-12, 33)	0.306	0.457	0 (0, 0)	0.016	0.594	0.010	0	0.06
PCB170	2 (-14, 20)	0.306	0.298	0 (0, 0)	0.016	0.281	0.005	0	0.07
OXC	-2 (-15, 13)	0.372	0.251	0 (0, 0)	0.058	0.191	0.011	0	0.02
TN	-7 (-21, 10)	0.372	0.299	0 (0, 0)	0.058	0.148	0.009	0	0.19
p,p'-DDE	2 (-8, 12)	0.372	0.126	0 (0, 0)	0.058	0.130	0.008	0	0.04
p,p'-DDT	-3 (-12, 7)	0.372	0.11	0 (0, 0)	0.058	0.061	0.004	0	0.06
HCB	0 (-11, 12)	0.372	0.112	0 (0, 0)	0.058	0.304	0.018	0	0
β-HCH	1 (-7, 8)	0.372	0.102	0 (0, 0)	0.058	0.200	0.012	0	0.01
PFOA	1 (-11, 15)	0.352	0.159	0 (0, 0)	0.044	0.230	0.010	0	0
PFNA	0 (-9, 10)	0.352	0.147	0 (0, 0)	0.044	0.402	0.018	0	0.01
PFHxS	-4 (-14, 8)	0.352	0.349	0 (0, 0)	0.044	0.184	0.008	0	0.07
PFOS	4 (-11, 22)	0.352	0.345	0 (0, 0)	0.044	0.195	0.009	0	0.01
1-NAP	-2 (-13, 9)	0.315	0.214	0 (0, 0)	0.021	0.167	0.004	0	0.02
2-NAP	3 (-9, 15)	0.315	0.254	0 (0, 0)	0.021	0.310	0.007	0	0.05
2-PHEN	-1 (-13, 13)	0.315	0.28	0 (0, 0)	0.021	0.024	0.001	0	0.01
3-PHEN	0 (-11, 12)	0.315	0.253	0 (0, 0)	0.021	0.500	0.011	0	0.01
TTMA	4 (-6, 17)	0.315	1	0 (0, 0)	0.008	1.000	0.008	0	0.03
DPHP	2 (-10, 15)	0.377	0.176	0 (0, 0)	0.036	0.194	0.007	0	0.05
BDCIPP	0 (-7, 7)	0.377	0.1	0 (0, 0)	0.036	0.069	0.003	0	0.04
BCIPHIPP	-2 (-13, 10)	0.377	0.305	0 (0, 0)	0.036	0.194	0.007	0	0.05
EHPHP	3 (-8, 15)	0.377	0.138	0 (0, 0)	0.036	0.222	0.008	0	0.09
BBOEHEP	-2 (-10, 6)	0.377	0.103	0 (0, 0)	0.036	0.139	0.005	0	0.1
5-OH-EHDPHP	5 (-7, 18)	0.377	0.179	0 (0, 0)	0.036	0.181	0.007	0	0.05
BPA	0 (-9, 10)	0.411	0.191	0 (0, 0)	0.021	0.415	0.009	0	0.02
BPF	-3 (-13, 8)	0.411	0.287	0 (0, 0)	0.021	0.049	0.001	0	0.04
BPS	7 (-3, 18)	0.411	0.522	0 (0, 0)	0.021	0.537	0.011	0	0.29
MEP	-1 (-7, 5)	0.509	0.023	0 (0, 0)	0.305	0.052	0.016	0	0.13
5cx-MEPP	-2 (-8, 5)	0.509	0.037	0 (0, 0)	0.305	0.033	0.010	0	0.06
5OH-MEHP	-3 (-11, 4)	0.509	0.034	0 (0, 0)	0.305	0.016	0.005	0	0.04

MiBP	1 (-8, 10)	0.509	0.036	0 (0, 0)	0.305	0.031	0.010	0	0.13
MnBP	28 (6, 54)	0.509	0.564	3 (0, 25)	0.305	0.752	0.230	3	0.7
MBzP	5 (-6, 17)	0.509	0.111	0 (0, 0)	0.305	0.108	0.033	0	0.42
MEHP	0 (-4, 3)	0.509	0.019	0 (0, 0)	0.305	0.028	0.009	0	0.01
OHMEHTP	-2 (-8, 4)	0.509	0.041	0 (0, 0)	0.305	0.067	0.021	0	0.09
MHNP	0 (-4, 3)	0.509	0.013	0 (0, 0)	0.305	0.023	0.007	0	0
MCOP	0 (-6, 5)	0.509	0.025	0 (0, 0)	0.305	0.026	0.008	0	0
MHNCH	0 (-5, 6)	0.509	0.025	0 (0, 0)	0.305	0.016	0.005	0	0.01
OHMIDP	1 (-4, 6)	0.509	0.027	0 (0, 0)	0.305	0.030	0.009	0	0.04
CXMIDP	0 (-4, 4)	0.509	0.021	0 (0, 0)	0.305	0.018	0.006	0	0.03
OXOMIDP	0 (-4, 4)	0.509	0.024	0 (0, 0)	0.305	0.031	0.010	0	0.01

Abbreviations: BKMR = Bayesian kernel machine regression; BMA = Bayesian model averaging; ENET = elastic net.

Figure S 4. Overall effect of the mixture on FeNo estimated by BKMR.

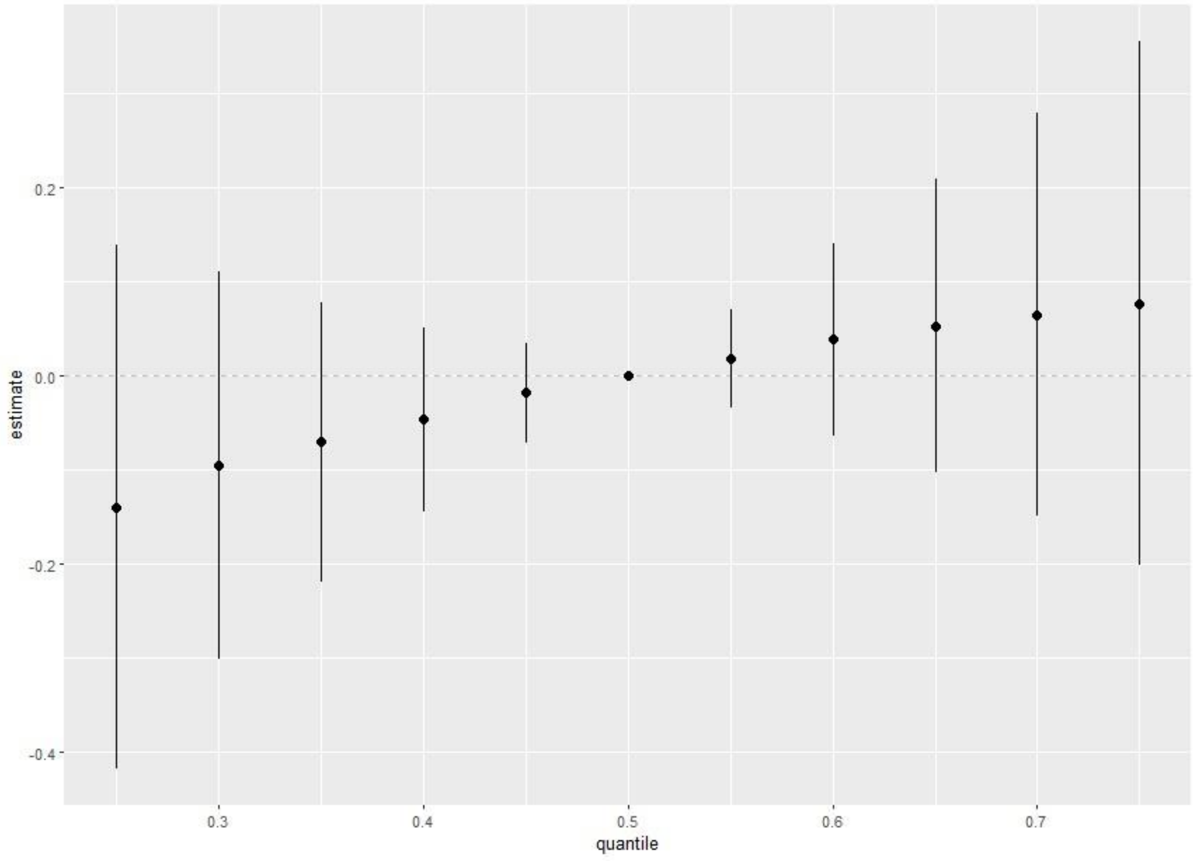

Asthma (last year)

Table S 8. Associations between exposure biomarker concentrations and asthma (last year) in teenagers estimated by the different statistical methods.

Exposure	BKMR			BMA				ENET	
	OR (95%Crl)	group PIP	cond PIP	OR (95%Crl)	Group PIP	CondPIP	PIP	OR	selection probability
Pb (blood)	1 (0.854, 1.17)	0.449	0.128	0.999 (0.94, 1.062)	0.027	0.143	0.004	1	0.03
Mn (blood)	0.958 (0.791, 1.161)	0.449	0.143	1.001 (0.92, 1.089)	0.027	0.207	0.006	1	0.03
Cu (blood)	1.086 (0.902, 1.307)	0.449	0.462	1.014 (0.742, 1.385)	0.027	0.355	0.01	1	0.25
Cd (urine)	0.962 (0.796, 1.163)	0.449	0.125	0.999 (0.957, 1.044)	0.027	0.111	0.003	1	0.01
Th (urine)	1.022 (0.829, 1.26)	0.449	0.141	1.002 (0.906, 1.108)	0.027	0.22	0.006	1	0.04
3-PBA	1.063 (0.863, 1.31)	0.449	0.289	1.001 (0.964, 1.039)	0.027	0.235	0.006	1	0.05
2,4-D	0.972 (0.767, 1.232)	0.449	0.278	0.999 (0.971, 1.029)	0.027	0.133	0.004	1	0.1
TCPY	1.071 (0.849, 1.351)	0.449	0.433	1.004 (0.917, 1.1)	0.027	0.665	0.018	1	0.09
PCB118	0.729 (0.488, 1.087)	0.758	0.746	0.797 (0.314, 2.026)	0.224	0.926	0.207	0.955	0.75
PCB187	1.011 (0.761, 1.344)	0.758	0.104	0.998 (0.949, 1.05)	0.224	0.026	0.006	1	0.04
PCB170	1.02 (0.696, 1.495)	0.758	0.15	0.995 (0.887, 1.115)	0.224	0.059	0.013	1	0.11
OXC	1.026 (0.845, 1.246)	0.471	0.128	1 (0.94, 1.063)	0.07	0.122	0.009	1	0
TN	0.988 (0.802, 1.215)	0.471	0.198	0.996 (0.91, 1.091)	0.07	0.125	0.009	1	0.09
p,p'-DDE	1.001 (0.835, 1.2)	0.471	0.125	0.995 (0.908, 1.091)	0.07	0.24	0.017	1	0.05
p,p'-DDT	1.02 (0.876, 1.189)	0.471	0.128	1 (0.98, 1.02)	0.07	0.102	0.007	1	0.03
HCB	0.944 (0.707, 1.259)	0.471	0.279	0.982 (0.741, 1.301)	0.07	0.304	0.021	1	0.2
β-HCH	0.981 (0.802, 1.2)	0.471	0.141	0.997 (0.924, 1.077)	0.07	0.121	0.009	1	0.1
PFOA	1.007 (0.803, 1.263)	0.414	0.201	1 (0.935, 1.07)	0.04	0.092	0.004	1	0.04
PFNA	1.004 (0.781, 1.29)	0.414	0.236	0.999 (0.915, 1.09)	0.04	0.441	0.018	1	0
PFHxS	1.014 (0.81, 1.27)	0.414	0.326	0.999 (0.954, 1.046)	0.04	0.114	0.005	1	0.02
PFOS	0.983 (0.692, 1.397)	0.414	0.236	0.995 (0.896, 1.104)	0.04	0.407	0.016	1	0.08
1-NAP	0.969 (0.762, 1.231)	0.437	0.24	1 (0.975, 1.026)	0.007	0.276	0.002	1	0.01
2-NAP	1.039 (0.835, 1.293)	0.437	0.269	1 (1, 1)	0.007	0	0	1	0.09
2-PHEN	0.991 (0.719, 1.365)	0.437	0.278	1.001 (0.96, 1.043)	0.007	0.454	0.003	1	0.06
3-PHEN	1.008 (0.756, 1.344)	0.437	0.213	1 (0.973, 1.028)	0.007	0.27	0.002	1	0.03
TTMA	0.974 (0.763, 1.244)	0.454	1	1 (0.971, 1.03)	0.008	1	0.008	1	0.07
DPHP	0.986 (0.833, 1.167)	0.462	0.108	1 (0.972, 1.028)	0.044	0.086	0.004	1	0
BDCIPP	1.002 (0.861, 1.164)	0.462	0.121	1 (0.985, 1.015)	0.044	0.127	0.006	1	0.03
BCIPHIPP	1.094 (0.906, 1.322)	0.462	0.201	1.003 (0.951, 1.057)	0.044	0.281	0.012	1.001	0.28
EHPHP	1.013 (0.854, 1.201)	0.462	0.131	1 (0.971, 1.031)	0.044	0.116	0.005	1	0
BBOEHEP	1.028 (0.853, 1.238)	0.462	0.313	1.001 (0.966, 1.037)	0.044	0.187	0.008	1	0.07
5-OH-EHDPHP	1.016 (0.828, 1.245)	0.462	0.126	1.001 (0.962, 1.041)	0.044	0.236	0.01	1	0.03
BPA	1.004 (0.847, 1.191)	0.479	0.237	0.999 (0.96, 1.04)	0.031	0.436	0.014	1	0.09
BPF	0.963 (0.789, 1.176)	0.479	0.241	1 (0.98, 1.02)	0.031	0.161	0.005	1	0.03
BPS	0.996 (0.817, 1.215)	0.479	0.522	0.999 (0.965, 1.034)	0.031	0.403	0.013	1	0.1
MEP	1.021 (0.865, 1.205)	0.431	0.157	1.001 (0.972, 1.03)	0.058	0.096	0.006	1	0.21
5cx-MEPP	0.985 (0.83, 1.167)	0.431	0.072	0.996 (0.897, 1.106)	0.058	0.164	0.009	1	0.13
5OH-MEHP	0.984 (0.819, 1.182)	0.431	0.053	0.999 (0.96, 1.04)	0.058	0.071	0.004	1	0.05
MiBP	0.993 (0.884, 1.115)	0.431	0.033	1 (0.984, 1.017)	0.058	0.031	0.002	1	0.02
MnBP	0.999 (0.838, 1.191)	0.431	0.057	1 (0.965, 1.037)	0.058	0.087	0.005	1	0.04

MBzP	1.016 (0.901, 1.145)	0.431	0.072	1 (0.994, 1.006)	0.058	0.008	0	1	0.04
MEHP	0.991 (0.884, 1.11)	0.431	0.04	0.999 (0.97, 1.03)	0.058	0.085	0.005	1	0.05
OHMEHTP	1.002 (0.907, 1.106)	0.431	0.051	0.999 (0.963, 1.036)	0.058	0.124	0.007	1	0.06
MHNP	0.946 (0.781, 1.148)	0.431	0.22	0.998 (0.941, 1.06)	0.058	0.057	0.003	0.996	0.3
MCOP	0.987 (0.86, 1.133)	0.431	0.088	0.998 (0.943, 1.057)	0.058	0.11	0.006	1	0.06
MHNCH	1.005 (0.906, 1.116)	0.431	0.028	1 (0.986, 1.014)	0.058	0.031	0.002	1	0.06
OHMIDP	1.009 (0.91, 1.118)	0.431	0.03	1 (0.977, 1.023)	0.058	0.075	0.004	1	0.04
CXMIDP	0.984 (0.866, 1.118)	0.431	0.044	0.999 (0.945, 1.056)	0.058	0.066	0.004	1	0.02
OXOMIDP	0.997 (0.88, 1.129)	0.431	0.055	1 (0.98, 1.02)	0.058	0.045	0.003	1	0.06

Abbreviations: BKMR = Bayesian kernel machine regression; BMA = Bayesian model averaging; ENET = elastic net.

Figure S 5. Univariate exposure–response functions of each pollutant on asthma estimated by BKMR.

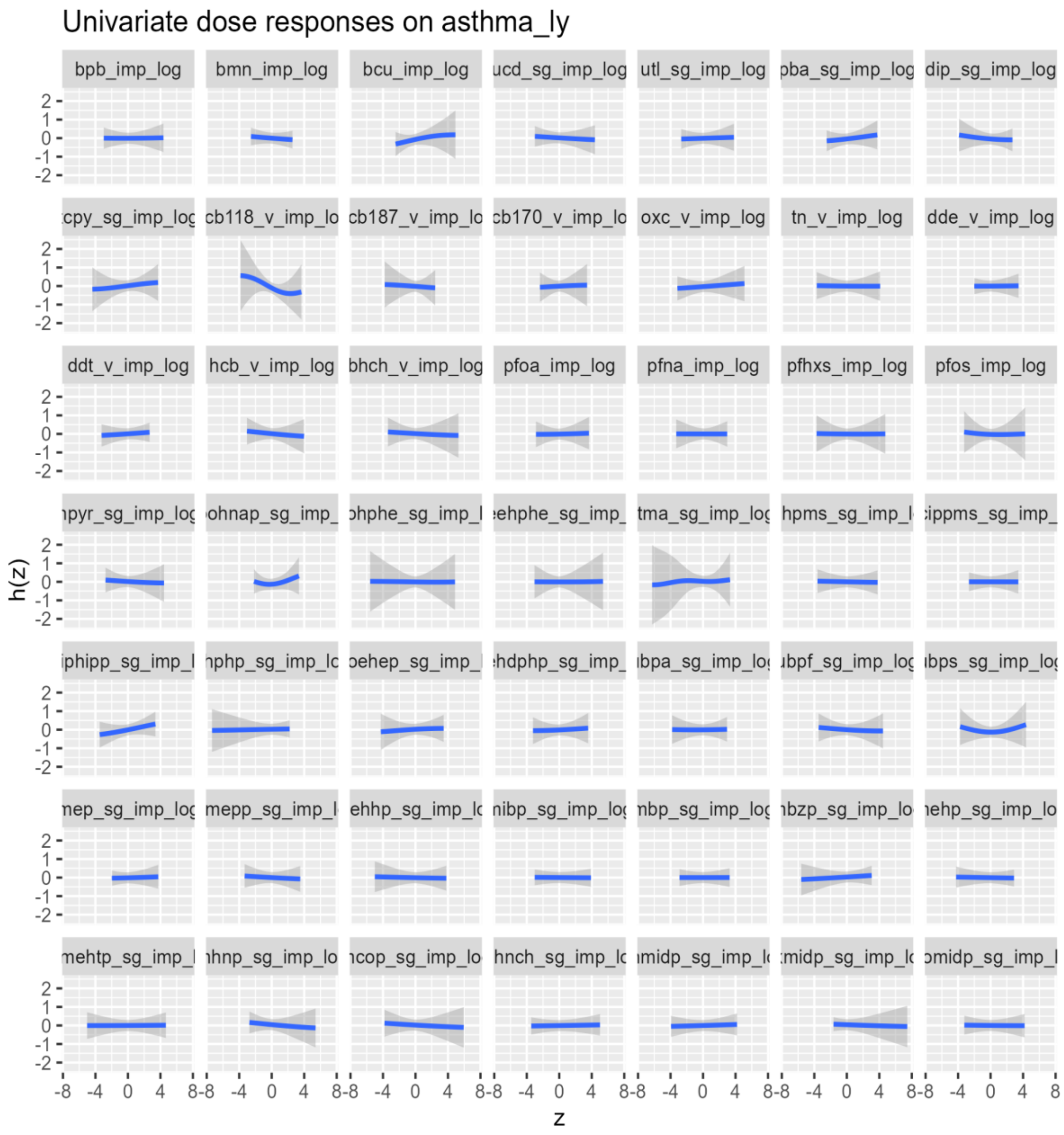

Figure S 6. Overall effect of the mixture on asthma estimated by BKMR.

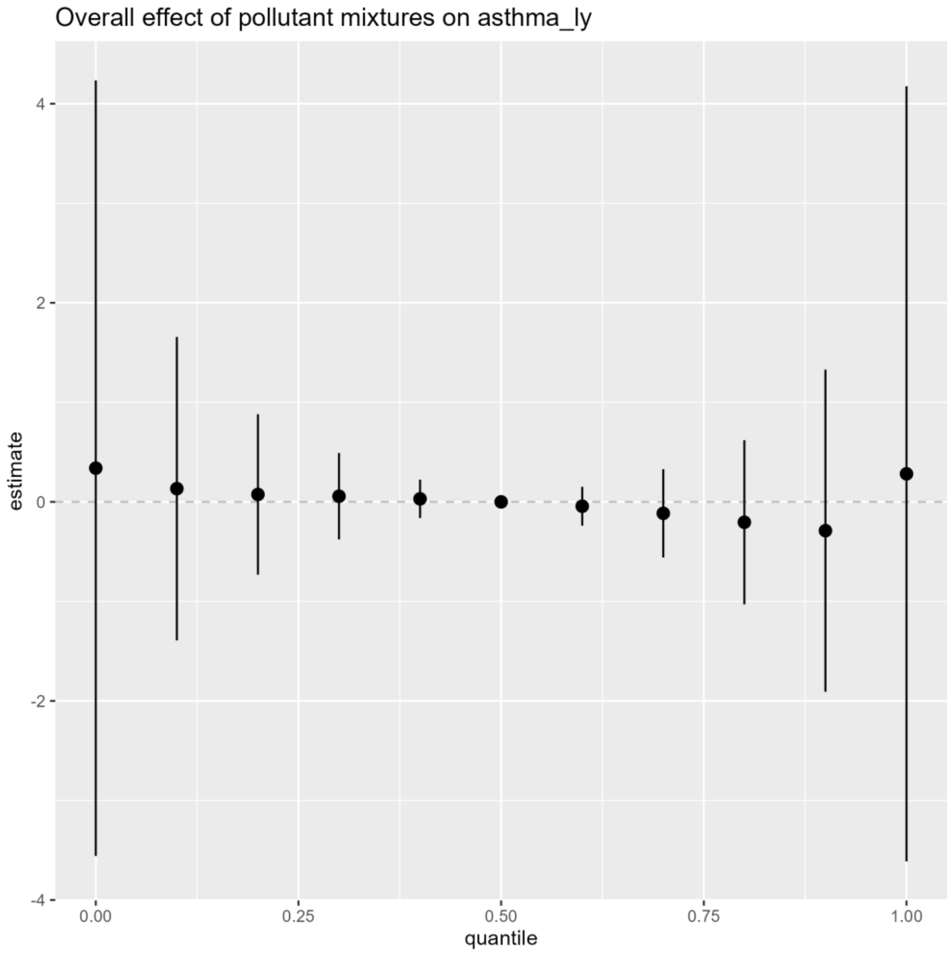

Figure S 7. Individual pollutant effects on asthma estimated by BKMR (the points are point estimates (β) and the bars are 95% CIs).

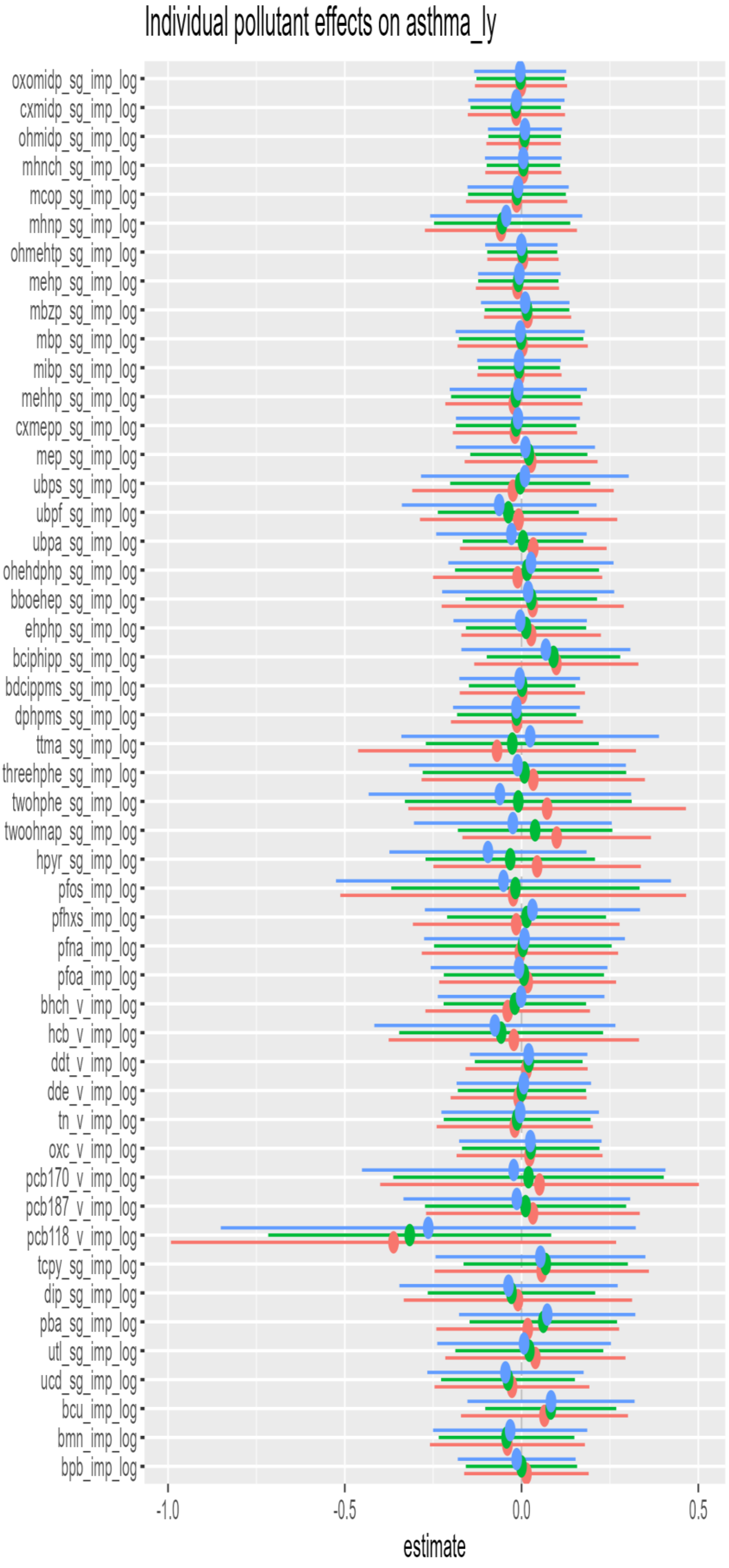

Rhinitis

Table S 9. Associations between exposure biomarker concentrations and rhinitis (ever) in teenagers estimated by the different statistical methods.

Exposure	BKMR			BMA				ENET	
	OR (95%CrI)	group PIP	cond PIP	OR (95%CrI)	Group PIP	CondPIP	PIP	OR	selection probability
Pb (blood)	1.012 (0.842, 1.216)	0.705	0.078	0.998 (0.936, 1.065)	0.083	0.065	0.005	1	0.06
Mn (blood)	0.929 (0.754, 1.145)	0.705	0.056	1 (1, 1)	0.083	0	0	1	0
Cu (blood)	1.058 (0.825, 1.356)	0.705	0.609	1.012 (0.785, 1.305)	0.083	0.144	0.012	1	0.18
Cd (urine)	1.038 (0.837, 1.286)	0.705	0.067	1.002 (0.941, 1.066)	0.083	0.1	0.008	1	0.03
Th (urine)	0.868 (0.656, 1.15)	0.705	0.19	0.935 (0.544, 1.608)	0.083	0.793	0.066	0.931	0.45
3-PBA	1.048 (0.828, 1.327)	0.493	0.294	1 (0.989, 1.011)	0.021	0.078	0.002	1	0.03
2,4-D	1.003 (0.764, 1.316)	0.493	0.463	0.997 (0.937, 1.06)	0.021	0.781	0.016	1	0.14
TCPY	0.933 (0.734, 1.186)	0.493	0.244	1 (0.976, 1.024)	0.021	0.141	0.003	1	0
PCB118	0.92 (0.662, 1.277)	0.524	0.358	0.999 (0.954, 1.047)	0.036	0.173	0.006	1	0.01
PCB187	0.938 (0.673, 1.309)	0.524	0.287	1 (0.971, 1.03)	0.036	0.261	0.009	1	0.02
PCB170	1.158 (0.761, 1.761)	0.524	0.355	1.006 (0.911, 1.111)	0.036	0.566	0.021	1	0.09
OXC	0.926 (0.701, 1.224)	0.565	0.132	1 (0.963, 1.038)	0.045	0.188	0.008	1	0
TN	1.063 (0.802, 1.408)	0.565	0.171	1 (0.982, 1.018)	0.045	0.037	0.002	1	0.01
p,p'-DDE	0.944 (0.726, 1.228)	0.565	0.183	0.994 (0.908, 1.089)	0.045	0.556	0.025	1	0.11
p,p'-DDT	0.917 (0.746, 1.127)	0.565	0.245	1 (0.986, 1.013)	0.045	0.036	0.002	0.993	0.25
HCB	0.988 (0.765, 1.276)	0.565	0.14	1 (0.97, 1.031)	0.045	0.055	0.002	1	0.01
β-HCH	1.068 (0.861, 1.323)	0.565	0.13	1.001 (0.968, 1.034)	0.045	0.129	0.006	1	0.03
PFOA	1.073 (0.826, 1.393)	0.508	0.2	1.002 (0.935, 1.073)	0.027	0.267	0.007	1	0
PFNA	1.03 (0.78, 1.361)	0.508	0.272	1 (0.967, 1.033)	0.027	0.206	0.006	1	0
PFHxS	0.943 (0.772, 1.153)	0.508	0.245	0.999 (0.961, 1.039)	0.027	0.278	0.007	1	0.04
PFOS	1.003 (0.733, 1.373)	0.508	0.283	0.999 (0.964, 1.036)	0.027	0.249	0.007	1	0
1-NAP	0.961 (0.749, 1.233)	0.662	0.157	0.999 (0.958, 1.042)	0.069	0.09	0.006	1	0.08
2-NAP	1.189 (0.883, 1.602)	0.662	0.565	1.018 (0.868, 1.195)	0.069	0.796	0.055	1.027	0.43
2-PHEN	1.048 (0.77, 1.427)	0.662	0.137	1.001 (0.965, 1.038)	0.069	0.078	0.005	1	0.02
3-PHEN	1.007 (0.727, 1.394)	0.662	0.141	1 (0.976, 1.024)	0.069	0.036	0.003	1	0.04
TTMA	0.939 (0.713, 1.236)	0.585	1	1 (0.99, 1.011)	0.002	1	0.002	1	0.01
DPHP	1.068 (0.806, 1.415)	0.524	0.241	1 (0.986, 1.014)	0.041	0.038	0.002	1	0.02
BDCIPP	0.968 (0.8, 1.172)	0.524	0.179	1 (0.986, 1.014)	0.041	0.171	0.007	1	0.02
BCIPHIPP	1.031 (0.853, 1.247)	0.524	0.113	1 (0.991, 1.008)	0.041	0.047	0.002	1	0
EHPHP	0.924 (0.732, 1.168)	0.524	0.2	0.996 (0.924, 1.073)	0.041	0.511	0.021	1	0.14
BBOEHEP	0.958 (0.781, 1.175)	0.524	0.136	1 (0.978, 1.021)	0.041	0.172	0.007	1	0.03
5-OH-EHDPHP	0.985 (0.791, 1.225)	0.524	0.131	1 (0.986, 1.014)	0.041	0.061	0.002	1	0
BPA	1.043 (0.866, 1.256)	0.658	0.121	1 (0.986, 1.014)	0.023	0.151	0.003	1	0.02
BPF	0.801 (0.572, 1.121)	0.658	0.752	0.997 (0.947, 1.05)	0.023	0.562	0.013	0.988	0.33
BPS	1.028 (0.871, 1.213)	0.658	0.127	1 (0.983, 1.018)	0.023	0.288	0.007	1	0.01
MEP	0.942 (0.8, 1.109)	0.588	0.066	0.999 (0.964, 1.035)	0.247	0.033	0.008	0.995	0.23
5cx-MEPP	0.963 (0.761, 1.219)	0.588	0.08	0.998 (0.938, 1.063)	0.247	0.041	0.01	1	0.02
5OH-MEHP	0.958 (0.766, 1.199)	0.588	0.07	0.999 (0.968, 1.032)	0.247	0.031	0.008	1	0.01
MiBP	0.991 (0.738, 1.332)	0.588	0.083	1 (0.986, 1.014)	0.247	0.008	0.002	1	0.03
MnBP	1.148 (0.798, 1.653)	0.588	0.084	1.002 (0.948, 1.06)	0.247	0.044	0.011	1	0.11

MBzP	1.083 (0.905, 1.296)	0.588	0.068	1 (0.984, 1.017)	0.247	0.013	0.003	1	0.15
MEHP	0.984 (0.87, 1.113)	0.588	0.032	1 (0.978, 1.022)	0.247	0.025	0.006	1	0.03
OHMEHTP	1.007 (0.891, 1.139)	0.588	0.037	1.001 (0.966, 1.038)	0.247	0.059	0.014	1	0.03
MHNP	1.025 (0.874, 1.202)	0.588	0.037	1.001 (0.969, 1.033)	0.247	0.026	0.006	1	0.04
MCOP	1.006 (0.867, 1.167)	0.588	0.04	1 (0.984, 1.016)	0.247	0.011	0.003	1	0.01
MHNCH	0.86 (0.668, 1.107)	0.588	0.213	0.929 (0.676, 1.278)	0.247	0.766	0.189	0.973	0.64
OHMIDP	0.999 (0.858, 1.163)	0.588	0.048	1 (0.988, 1.012)	0.247	0.009	0.002	1	0.03
CXMIDP	1.015 (0.907, 1.137)	0.588	0.036	1.002 (0.945, 1.061)	0.247	0.035	0.009	1	0.06
OXOMIDP	1.017 (0.831, 1.244)	0.588	0.104	1 (0.985, 1.015)	0.247	0.012	0.003	1	0.01

Abbreviations: BKMR = Bayesian kernel machine regression; BMA = Bayesian model averaging; ENET = elastic net.

Figure S 8. Univariate exposure–response functions of each pollutant on rhinitis estimated by BKMR.

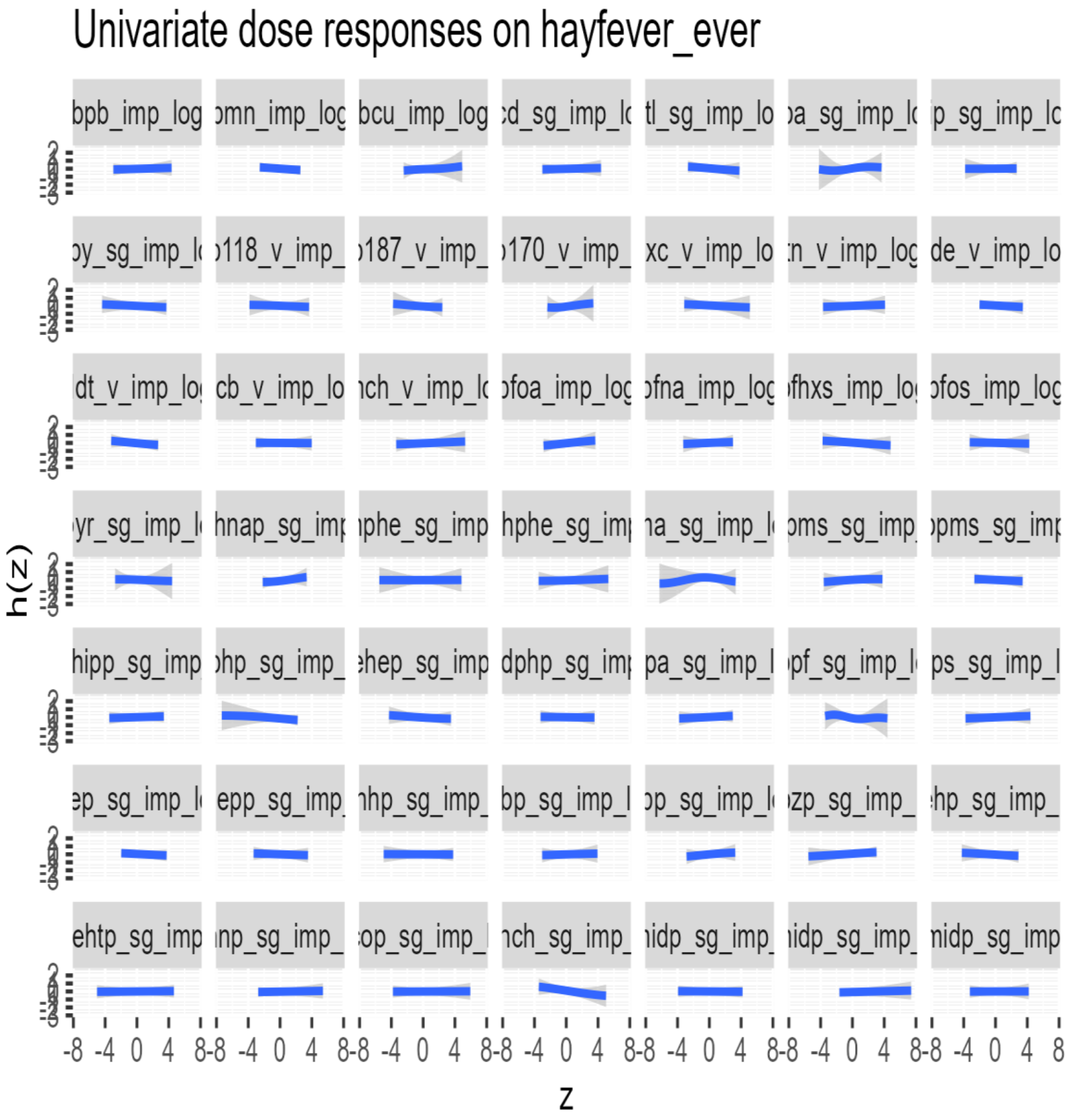

Figure S 9. Overall effect of the mixture on rhinitis estimated by BKMR.

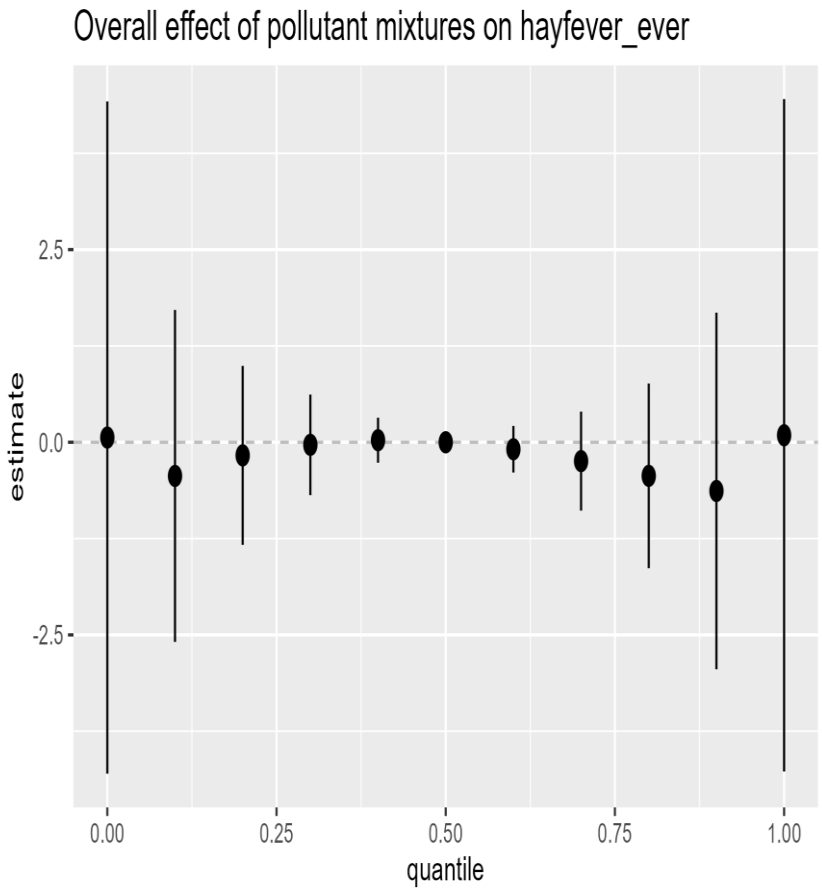

Eczema (ever)

Table S 10. Associations between exposure biomarker concentrations and eczema (ever) in teenagers estimated by the different statistical methods.

Exposure	BKMR			BMA				ENET	
	OR (95%Crl)	group PIP	cond PIP	OR (95%Crl)	Group PIP	CondPIP	PIP	OR	selection probability
Pb (blood)	0.943 (0.639, 1.391)	0.535	0.149	1 (0.954, 1.048)	0.021	0.214	0.005	1	0.06
Mn (blood)	0.999 (0.621, 1.605)	0.535	0.136	0.997 (0.903, 1.102)	0.021	0.34	0.007	1	0.14
Cu (blood)	0.981 (0.611, 1.577)	0.535	0.473	0.998 (0.885, 1.125)	0.021	0.209	0.004	1	0.06
Cd (urine)	1 (0.772, 1.294)	0.535	0.091	1 (0.965, 1.036)	0.021	0.164	0.003	1	0.09
Th (urine)	0.946 (0.671, 1.333)	0.535	0.15	0.999 (0.957, 1.044)	0.021	0.073	0.002	1	0.02
3-PBA	1.032 (0.609, 1.749)	0.483	0.282	1 (0.984, 1.017)	0.011	0.261	0.003	1	0.07
2,4-D	1.083 (0.646, 1.817)	0.483	0.351	0.999 (0.973, 1.026)	0.011	0.361	0.004	1	0.12
TCPY	0.857 (0.49, 1.499)	0.483	0.368	0.999 (0.968, 1.032)	0.011	0.378	0.004	1	0.05
PCB118	1.052 (0.581, 1.902)	0.563	0.292	1 (0.971, 1.03)	0.045	0.049	0.002	1	0.01
PCB187	1.256 (0.415, 3.798)	0.563	0.354	1.002 (0.954, 1.052)	0.045	0.209	0.009	1	0.02
PCB170	1.01 (0.537, 1.901)	0.563	0.353	1.014 (0.858, 1.2)	0.045	0.741	0.033	1	0.33
OXC	1.014 (0.707, 1.456)	0.595	0.131	1.006 (0.904, 1.118)	0.079	0.167	0.013	1	0.18
TN	1.161 (0.638, 2.113)	0.595	0.321	1.026 (0.813, 1.293)	0.079	0.652	0.052	1	0.43
p,p'-DDE	0.794 (0.251, 2.508)	0.595	0.146	1 (0.979, 1.022)	0.079	0.044	0.003	1	0.03
p,p'-DDT	1.031 (0.725, 1.468)	0.595	0.139	1.001 (0.976, 1.027)	0.079	0.102	0.008	1	0.12
HCB	0.887 (0.584, 1.348)	0.595	0.136	0.999 (0.957, 1.044)	0.079	0.035	0.003	1	0.08
β-HCH	0.991 (0.728, 1.35)	0.595	0.127	1 (1, 1)	0.079	0	0	1	0
PFOA	0.857 (0.539, 1.363)	0.456	0.241	1 (0.974, 1.027)	0.011	0.124	0.001	1	0.02
PFNA	1.045 (0.671, 1.626)	0.456	0.287	1 (0.965, 1.037)	0.011	0.41	0.005	1	0.04
PFHxS	1.007 (0.685, 1.48)	0.456	0.243	1 (0.976, 1.025)	0.011	0.151	0.002	1	0.07
PFOS	1.09 (0.709, 1.675)	0.456	0.229	1.001 (0.967, 1.036)	0.011	0.314	0.004	1	0.1
1-NAP	0.913 (0.571, 1.458)	0.587	0.212	0.998 (0.929, 1.07)	0.024	0.295	0.007	1	0.18
2-NAP	0.986 (0.604, 1.607)	0.587	0.276	1.002 (0.952, 1.054)	0.024	0.351	0.008	1	0.34
2-PHEN	1.023 (0.55, 1.901)	0.587	0.243	1 (0.974, 1.026)	0.024	0.094	0.002	1	0.01
3-PHEN	1.027 (0.524, 2.013)	0.587	0.268	0.999 (0.954, 1.046)	0.024	0.28	0.007	1	0.04
TTMA	0.763 (0.421, 1.386)	0.522	1	1 (0.975, 1.024)	0.007	1	0.007	1	0.04
DPHP	1.002 (0.72, 1.394)	0.498	0.151	1 (0.981, 1.019)	0.032	0.063	0.002	1	0.04
BDCIPP	1.119 (0.804, 1.557)	0.498	0.165	1 (0.992, 1.009)	0.032	0.06	0.002	1	0.08
BCIPHIPP	1.097 (0.82, 1.468)	0.498	0.155	1 (0.99, 1.01)	0.032	0.086	0.003	1	0.1
EHPHP	0.976 (0.719, 1.325)	0.498	0.134	0.997 (0.938, 1.06)	0.032	0.534	0.017	1	0.17
BBOEHEP	0.956 (0.68, 1.345)	0.498	0.201	0.999 (0.972, 1.028)	0.032	0.307	0.01	1	0.06
5-OH-EHDPHP	1.084 (0.715, 1.644)	0.498	0.193	1 (0.975, 1.025)	0.032	0.218	0.007	1	0.03
BPA	1.167 (0.707, 1.925)	0.528	0.344	1 (0.977, 1.023)	0.018	0.334	0.006	1	0.02
BPF	0.937 (0.637, 1.378)	0.528	0.369	1 (0.982, 1.018)	0.018	0.37	0.007	1	0.11
BPS	1.02 (0.73, 1.427)	0.528	0.288	1 (0.983, 1.017)	0.018	0.296	0.005	1	0.05
MEP	0.949 (0.407, 2.217)	0.496	0.128	1.001 (0.978, 1.024)	0.065	0.113	0.007	1	0.12
5cx-MEPP	0.937 (0.683, 1.284)	0.496	0.083	0.999 (0.952, 1.049)	0.065	0.096	0.006	1	0.06
5OH-MEHP	0.922 (0.514, 1.652)	0.496	0.071	1 (0.99, 1.01)	0.065	0.013	0.001	1	0.02
MiBP	0.996 (0.829, 1.196)	0.496	0.062	1 (0.988, 1.013)	0.065	0.025	0.002	1	0.04
MnBP	1.054 (0.777, 1.429)	0.496	0.083	1 (1, 1)	0.065	0	0	1	0.1

MBzP	1.019 (0.838, 1.24)	0.496	0.057	1 (0.988, 1.012)	0.065	0.045	0.003	1	0.03
MEHP	1.017 (0.831, 1.245)	0.496	0.069	1.001 (0.964, 1.039)	0.065	0.1	0.006	1	0.09
OHMEHTP	0.997 (0.838, 1.186)	0.496	0.061	1 (0.976, 1.023)	0.065	0.154	0.01	1	0.05
MHNP	0.955 (0.66, 1.381)	0.496	0.06	1 (0.983, 1.017)	0.065	0.036	0.002	1	0.01
MCOP	0.979 (0.787, 1.218)	0.496	0.069	0.999 (0.957, 1.042)	0.065	0.179	0.012	1	0.08
MHNCH	0.996 (0.838, 1.184)	0.496	0.069	0.999 (0.968, 1.031)	0.065	0.086	0.006	1	0.27
OHMIDP	0.986 (0.796, 1.22)	0.496	0.054	1 (0.981, 1.019)	0.065	0.069	0.004	1	0.03
CXMIDP	0.953 (0.742, 1.224)	0.496	0.071	0.999 (0.94, 1.061)	0.065	0.103	0.007	1	0.11
OXOMIDP	0.981 (0.755, 1.275)	0.496	0.061	1 (0.983, 1.017)	0.065	0.052	0.003	1	0.01

Abbreviations: BKMR = Bayesian kernel machine regression; BMA = Bayesian model averaging; ENET = elastic net.

Figure S 10. Overall effect of the mixture on eczema estimated by BKMR.

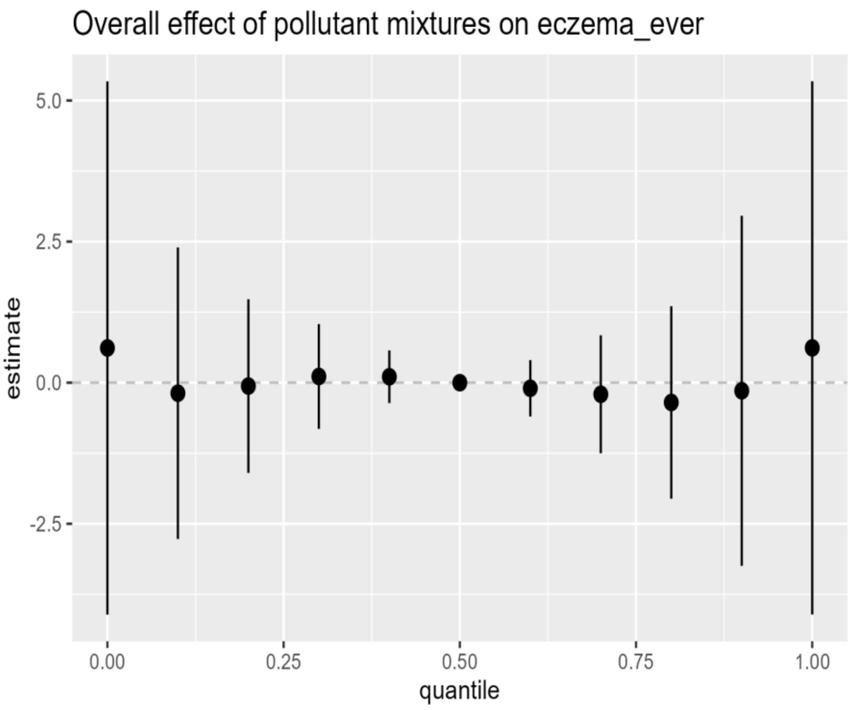

Allergy (skin)

Table S 11. Associations between exposure biomarker concentrations and skin allergy in teenagers estimated by the different statistical methods.

Exposure	BKMR			BMA				ENET	
	OR (95%CrI)	group PIP	cond PIP	OR (95%CrI)	Group PIP	CondPIP	PIP	OR	selection probability
Pb (blood)	0.998 (0.806, 1.236)	0.512	0.142	0.998 (0.93, 1.072)	0.016	0.338	0.006	1	0.07
Mn (blood)	0.894 (0.647, 1.235)	0.512	0.161	0.999 (0.944, 1.058)	0.016	0.165	0.003	1	0.07
Cu (blood)	1.034 (0.804, 1.33)	0.512	0.33	1 (0.932, 1.073)	0.016	0.159	0.003	1	0.07
Cd (urine)	1 (0.755, 1.324)	0.512	0.183	0.997 (0.91, 1.093)	0.016	0.337	0.006	1	0.18
Th (urine)	0.973 (0.757, 1.252)	0.512	0.184	1 (1, 1)	0.016	0	0	1	0.06
3-PBA	1.129 (0.828, 1.54)	0.479	0.354	1.001 (0.973, 1.029)	0.01	0.32	0.003	1	0.21
2,4-D	0.947 (0.721, 1.243)	0.479	0.328	1 (0.985, 1.015)	0.01	0.204	0.002	1	0.03
TCPY	0.901 (0.691, 1.175)	0.479	0.317	0.999 (0.954, 1.046)	0.01	0.476	0.005	1	0.25
PCB118	1.067 (0.777, 1.465)	0.423	0.345	1.001 (0.964, 1.039)	0.008	0.425	0.003	1	0.03
PCB187	1.043 (0.737, 1.477)	0.423	0.349	1 (1, 1)	0.008	0	0	1	0.14
PCB170	1.075 (0.7, 1.652)	0.423	0.307	1.001 (0.96, 1.044)	0.008	0.575	0.004	1	0.07
OXC	1.011 (0.781, 1.31)	0.447	0.155	1 (0.976, 1.025)	0.027	0.085	0.002	1	0.03
TN	1.03 (0.821, 1.292)	0.447	0.147	1.001 (0.965, 1.038)	0.027	0.195	0.005	1	0.06
p,p'-DDE	0.999 (0.799, 1.249)	0.447	0.184	1 (0.979, 1.021)	0.027	0.134	0.004	1	0.02
p,p'-DDT	0.992 (0.793, 1.243)	0.447	0.175	1 (0.986, 1.015)	0.027	0.136	0.004	1	0.04
HCB	0.852 (0.643, 1.128)	0.447	0.208	0.995 (0.873, 1.133)	0.027	0.326	0.009	1	0.24
β-HCH	0.977 (0.8, 1.194)	0.447	0.132	1 (0.973, 1.027)	0.027	0.124	0.003	1	0.01
PFOA	0.906 (0.647, 1.269)	0.449	0.24	0.999 (0.941, 1.06)	0.019	0.198	0.004	1	0.07
PFNA	1.048 (0.751, 1.462)	0.449	0.293	1.001 (0.959, 1.045)	0.019	0.261	0.005	1	0.05
PFHxS	0.952 (0.765, 1.184)	0.449	0.259	0.998 (0.938, 1.062)	0.019	0.385	0.007	1	0.22
PFOS	1.001 (0.782, 1.282)	0.449	0.208	1 (0.974, 1.028)	0.019	0.156	0.003	1	0.02
1-NAP	1.037 (0.799, 1.345)	0.486	0.227	1 (0.97, 1.031)	0.023	0.134	0.003	1	0.04
2-NAP	0.874 (0.672, 1.137)	0.486	0.275	0.998 (0.949, 1.05)	0.023	0.275	0.006	1	0.33
2-PHEN	0.946 (0.683, 1.31)	0.486	0.293	0.999 (0.944, 1.058)	0.023	0.415	0.01	1	0.03
3-PHEN	1.006 (0.744, 1.359)	0.486	0.206	1 (0.958, 1.043)	0.023	0.198	0.005	1	0.02
TTMA	0.885 (0.631, 1.24)	0.454	1	1 (0.988, 1.012)	0.002	1	0.002	1	0.05
DPHP	1.026 (0.851, 1.238)	0.444	0.129	1 (1, 1)	0.025	0	0	1	0.05
BDCIPP	0.971 (0.814, 1.157)	0.444	0.167	1 (0.986, 1.014)	0.025	0.128	0.003	1	0.05
BCIPHIPP	1.066 (0.895, 1.269)	0.444	0.138	1.001 (0.97, 1.033)	0.025	0.439	0.011	1	0.17
EHPHP	0.974 (0.782, 1.215)	0.444	0.112	0.999 (0.951, 1.048)	0.025	0.282	0.007	1	0.08
BBOEHEP	0.931 (0.737, 1.178)	0.444	0.258	1 (0.988, 1.012)	0.025	0.032	0.001	1	0.17
5-OH-EHDPHP	1.023 (0.81, 1.291)	0.444	0.195	1 (0.978, 1.022)	0.025	0.12	0.003	1	0.02
BPA	0.945 (0.734, 1.217)	0.441	0.295	0.999 (0.971, 1.028)	0.012	0.506	0.006	1	0.06
BPF	1.041 (0.819, 1.324)	0.441	0.366	1 (0.983, 1.018)	0.012	0.327	0.004	1	0.08
BPS	1.046 (0.867, 1.263)	0.441	0.339	1 (0.989, 1.011)	0.012	0.167	0.002	1	0.05
MEP	1.01 (0.894, 1.14)	0.502	0.056	1 (1, 1)	0.066	0	0	1	0.02
5cx-MEPP	0.974 (0.794, 1.193)	0.502	0.066	0.997 (0.907, 1.095)	0.066	0.108	0.007	1	0.17
5OH-MEHP	1.011 (0.886, 1.154)	0.502	0.042	1 (0.972, 1.03)	0.066	0.084	0.006	1	0.01
MiBP	1.006 (0.821, 1.234)	0.502	0.089	1 (0.982, 1.019)	0.066	0.045	0.003	1	0.02
MnBP	1.01 (0.809, 1.261)	0.502	0.06	1 (0.976, 1.024)	0.066	0.048	0.003	1	0.05

MBzP	1.003 (0.877, 1.148)	0.502	0.05	1 (0.985, 1.015)	0.066	0.068	0.004	1	0.06
MEHP	1.014 (0.892, 1.153)	0.502	0.061	1 (0.985, 1.016)	0.066	0.032	0.002	1	0.05
OHMEHTP	0.958 (0.833, 1.103)	0.502	0.086	0.998 (0.956, 1.042)	0.066	0.149	0.01	1	0.27
MHNP	0.996 (0.873, 1.137)	0.502	0.062	0.999 (0.962, 1.037)	0.066	0.068	0.004	1	0.03
MCOP	1.011 (0.854, 1.197)	0.502	0.071	0.999 (0.971, 1.029)	0.066	0.061	0.004	1	0.01
MHNCH	1.052 (0.852, 1.298)	0.502	0.059	1 (0.985, 1.015)	0.066	0.047	0.003	1	0.03
OHMIDP	0.987 (0.855, 1.14)	0.502	0.066	1 (0.973, 1.026)	0.066	0.089	0.006	1	0.02
CXMIDP	0.877 (0.645, 1.191)	0.502	0.169	0.995 (0.875, 1.13)	0.066	0.129	0.009	1	0.31
OXOMIDP	0.987 (0.867, 1.124)	0.502	0.064	1 (0.974, 1.026)	0.066	0.074	0.005	1	0.01

Abbreviations: BKMR = Bayesian kernel machine regression; BMA = Bayesian model averaging; ENET = elastic net

Figure S 11. Overall effect of the mixture on skin allergy estimated by BKMR.

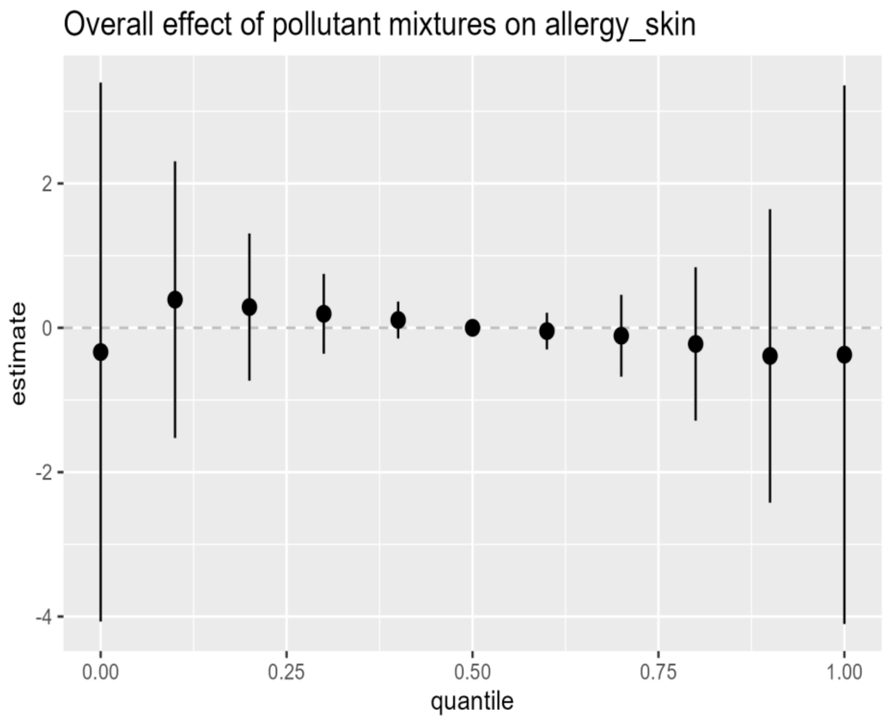

Allergy (Nguyen, Higashi et al.)

Table S 12. Associations between exposure biomarker concentrations and all kinds of allergy in teenagers estimated by the different statistical methods.

Exposure	BKMR			BMA				ENET	
	OR (95%CrI)	group PIP	cond PIP	OR (95%CrI)	Group PIP	CondPIP	PIP	OR	selection probability
Pb (blood)	0.986 (0.8, 1.217)	0.463	0.106	0.998 (0.936, 1.065)	0.017	0.302	0.005	1	0.14
Mn (blood)	1.019 (0.83, 1.251)	0.463	0.117	1 (0.96, 1.042)	0.017	0.132	0.002	1	0.09
Cu (blood)	0.93 (0.733, 1.182)	0.463	0.575	0.999 (0.91, 1.096)	0.017	0.263	0.004	1	0.05
Cd (urine)	1.06 (0.833, 1.35)	0.463	0.085	1 (0.974, 1.027)	0.017	0.097	0.002	1	0.04
Th (urine)	1.129 (0.898, 1.42)	0.463	0.117	1.001 (0.932, 1.075)	0.017	0.32	0.005	1	0.07
3-PBA	1.021 (0.822, 1.27)	0.425	0.339	1 (0.981, 1.02)	0.005	0.62	0.003	1	0.04
2,4-D	0.907 (0.696, 1.181)	0.425	0.295	1 (0.99, 1.01)	0.005	0.211	0.001	1	0.06
TCPY	0.965 (0.756, 1.233)	0.425	0.366	1 (0.989, 1.011)	0.005	0.169	0.001	1	0.06
PCB118	1.076 (0.794, 1.46)	0.412	0.332	1 (0.983, 1.018)	0.008	0.149	0.001	1	0.03
PCB187	1.006 (0.707, 1.43)	0.412	0.328	1 (0.986, 1.014)	0.008	0.328	0.002	1	0.01
PCB170	1.134 (0.77, 1.67)	0.412	0.34	1 (0.974, 1.027)	0.008	0.523	0.004	1	0.06
OXC	1.063 (0.817, 1.382)	0.366	0.183	1 (0.971, 1.03)	0.023	0.162	0.004	1	0.08
TN	1.047 (0.866, 1.266)	0.366	0.135	1 (0.971, 1.03)	0.023	0.234	0.005	1	0.02
p,p'-DDE	0.95 (0.756, 1.195)	0.366	0.17	1 (1, 1)	0.023	0	0	1	0.04
p,p'-DDT	1.017 (0.868, 1.192)	0.366	0.127	1 (0.984, 1.017)	0.023	0.317	0.007	1	0.08
HCB	0.839 (0.625, 1.126)	0.366	0.276	0.998 (0.931, 1.071)	0.023	0.161	0.004	1	0.27
β-HCH	0.997 (0.836, 1.189)	0.366	0.109	1 (0.979, 1.021)	0.023	0.125	0.003	1	0.03
PFOA	0.932 (0.675, 1.288)	0.472	0.258	0.997 (0.907, 1.096)	0.02	0.291	0.006	1	0.22
PFNA	1.013 (0.779, 1.318)	0.472	0.177	0.999 (0.964, 1.036)	0.02	0.281	0.006	1	0.05
PFHxS	0.859 (0.649, 1.136)	0.472	0.442	0.997 (0.928, 1.072)	0.02	0.365	0.007	1	0.35
PFOS	1.036 (0.831, 1.293)	0.472	0.123	1 (0.987, 1.013)	0.02	0.063	0.001	1	0.04
1-NAP	1.08 (0.836, 1.395)	0.42	0.232	1 (0.971, 1.031)	0.013	0.307	0.004	1	0.06
2-NAP	0.976 (0.766, 1.244)	0.42	0.205	1 (0.988, 1.012)	0.013	0.146	0.002	1	0.06
2-PHEN	1.011 (0.749, 1.363)	0.42	0.274	1 (0.968, 1.034)	0.013	0.343	0.004	1	0.02
3-PHEN	0.964 (0.708, 1.314)	0.42	0.289	1 (0.973, 1.027)	0.013	0.204	0.003	1	0.03
TTMA	0.997 (0.798, 1.244)	0.377	1	1 (0.982, 1.018)	0.005	1	0.005	1	0.07
DPHP	1.037 (0.804, 1.337)	0.501	0.145	1 (0.985, 1.015)	0.021	0.079	0.002	1	0.02
BDCIPP	0.889 (0.732, 1.079)	0.501	0.298	0.999 (0.978, 1.022)	0.021	0.188	0.004	1	0.42
BCIPHIPP	1.026 (0.873, 1.207)	0.501	0.097	1 (0.988, 1.012)	0.021	0.204	0.004	1	0.07
EHPHP	0.936 (0.661, 1.325)	0.501	0.122	1 (0.975, 1.025)	0.021	0.147	0.003	1	0.07
BBOEHEP	0.918 (0.73, 1.155)	0.501	0.24	0.999 (0.964, 1.035)	0.021	0.263	0.006	1	0.37
5-OH-EHDPHP	1.049 (0.848, 1.298)	0.501	0.099	1 (0.986, 1.015)	0.021	0.118	0.003	1	0.03
BPA	0.955 (0.793, 1.151)	0.385	0.315	1 (0.988, 1.012)	0.004	0.538	0.002	1	0.11
BPF	1.04 (0.843, 1.283)	0.385	0.282	1 (0.993, 1.008)	0.004	0.248	0.001	1	0.09
BPS	1.094 (0.905, 1.323)	0.385	0.403	1 (0.992, 1.008)	0.004	0.214	0.001	1	0.13
MEP	1.041 (0.769, 1.409)	0.41	0.1	1 (0.994, 1.006)	0.041	0.021	0.001	1	0.04
5cx-MEPP	0.981 (0.743, 1.294)	0.41	0.104	1 (0.978, 1.023)	0.041	0.04	0.002	1	0.04
5OH-MEHP	1.045 (0.86, 1.27)	0.41	0.078	1.001 (0.959, 1.045)	0.041	0.188	0.008	1	0.14
MiBP	1.119 (0.865, 1.447)	0.41	0.137	1.001 (0.968, 1.035)	0.041	0.136	0.006	1	0.24
MnBP	0.974 (0.714, 1.328)	0.41	0.052	1 (0.982, 1.019)	0.041	0.043	0.002	1	0.03

MBzP	1.012 (0.888, 1.153)	0.41	0.054	1 (0.983, 1.017)	0.041	0.175	0.007	1	0.03
MEHP	1.022 (0.898, 1.164)	0.41	0.07	1.001 (0.969, 1.034)	0.041	0.133	0.005	1	0.17
OHMEHTP	0.997 (0.896, 1.111)	0.41	0.051	1 (0.992, 1.008)	0.041	0.037	0.002	1	0.03
MHNP	1.035 (0.84, 1.275)	0.41	0.075	1 (0.981, 1.019)	0.041	0.085	0.003	1	0.03
MCOP	1.001 (0.897, 1.116)	0.41	0.042	1 (0.986, 1.015)	0.041	0.048	0.002	1	0.04
MHNCH	0.996 (0.889, 1.117)	0.41	0.074	1 (1, 1)	0.041	0	0	1	0.05
OHMIDP	0.992 (0.854, 1.151)	0.41	0.043	1 (0.984, 1.016)	0.041	0.083	0.003	1	0.01
CXMIDP	0.997 (0.892, 1.114)	0.41	0.072	1 (0.973, 1.027)	0.041	0.059	0.002	1	0.08
OXOMIDP	0.975 (0.832, 1.144)	0.41	0.048	1 (0.986, 1.014)	0.041	0.052	0.002	1	0.09

Abbreviations: BKMR = Bayesian kernel machine regression; BMA = Bayesian model averaging; ENET = elastic net.

Figure S 12. Overall effect of the mixture on allergy (any kinds) estimated by BKMR.

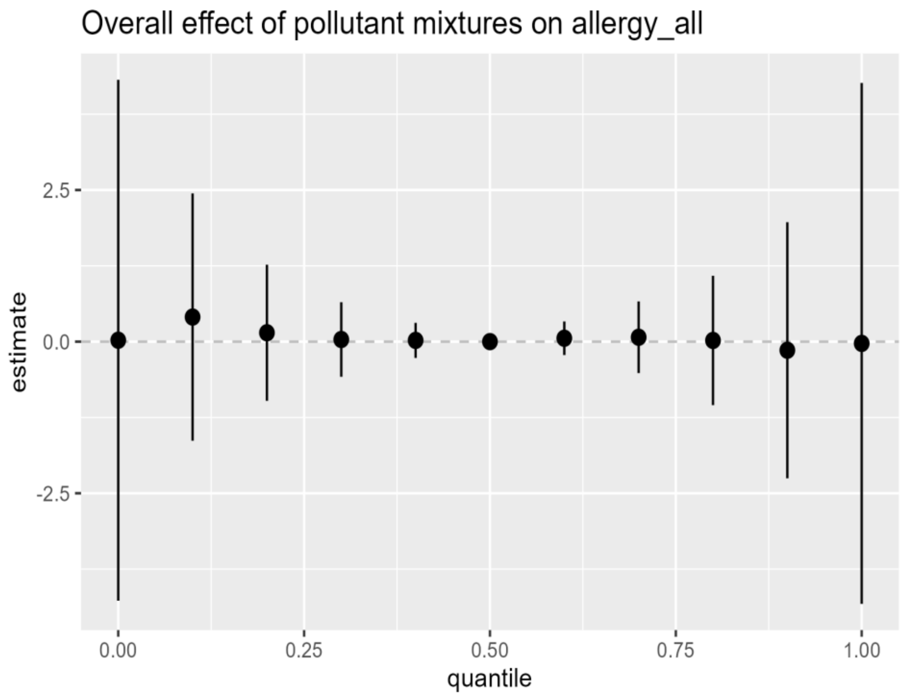

Lower respiratory tract infection

Table S 13. Associations between exposure biomarker concentrations and lower respiratory infection (last year) in teenagers estimated by the different statistical methods.

Exposure	BKMR			BMA				ENET	
	OR (95%CrI)	group PIP	cond PIP	OR (95%CrI)	Group PIP	CondPIP	PIP	OR	selection probability
Pb (blood)	0.922 (0.667, 1.273)	0.479	0.247	0.991 (0.821, 1.195)	0.025	0.649	0.016	1	0.06
Mn (blood)	0.901 (0.664, 1.223)	0.479	0.246	0.999 (0.927, 1.077)	0.025	0.115	0.003	1	0.05
Cu (blood)	0.984 (0.789, 1.226)	0.479	0.249	0.999 (0.88, 1.134)	0.025	0.153	0.004	1	0
Cd (urine)	1.015 (0.807, 1.276)	0.479	0.146	1 (1, 1)	0.025	0	0	1	0.04
Th (urine)	0.987 (0.764, 1.276)	0.479	0.112	0.999 (0.94, 1.062)	0.025	0.083	0.002	1	0.01
3-PBA	0.946 (0.694, 1.291)	0.475	0.442	1 (0.975, 1.025)	0.015	0.117	0.002	1	0.07
2,4-D	0.964 (0.717, 1.296)	0.475	0.253	1.001 (0.965, 1.038)	0.015	0.43	0.007	1	0
TCPY	0.939 (0.695, 1.27)	0.475	0.305	0.998 (0.941, 1.059)	0.015	0.453	0.007	1	0.09
PCB118	1.038 (0.741, 1.454)	0.538	0.24	1 (0.947, 1.057)	0.021	0.25	0.005	1	0.02
PCB187	1.158 (0.681, 1.97)	0.538	0.521	1.002 (0.932, 1.079)	0.021	0.403	0.008	1	0.02
PCB170	1.109 (0.669, 1.836)	0.538	0.238	1.002 (0.925, 1.086)	0.021	0.347	0.007	1	0.01
OXC	1.009 (0.794, 1.282)	0.622	0.053	1 (0.963, 1.038)	0.04	0.066	0.003	1	0
TN	1.052 (0.837, 1.321)	0.622	0.061	1.002 (0.947, 1.06)	0.04	0.151	0.006	1	0.02
p,p'-DDE	0.877 (0.563, 1.366)	0.622	0.151	0.999 (0.955, 1.045)	0.04	0.075	0.003	1	0.04
p,p'-DDT	1.029 (0.73, 1.451)	0.622	0.097	1 (1, 1)	0.04	0	0	1	0
HCB	0.545 (0.291, 1.019)	0.622	0.6	0.97 (0.651, 1.444)	0.04	0.708	0.028	1	0.41
β-HCH	0.99 (0.856, 1.144)	0.622	0.038	1 (1, 1)	0.04	0	0	1	0
PFOA	0.967 (0.645, 1.449)	0.699	0.157	1.004 (0.885, 1.139)	0.037	0.136	0.005	1	0.09
PFNA	1.298 (0.636, 2.65)	0.699	0.241	1.015 (0.813, 1.267)	0.037	0.55	0.02	1	0.32
PFHxS	1.001 (0.816, 1.227)	0.699	0.102	1 (0.974, 1.027)	0.037	0.047	0.002	1	0.07
PFOS	0.887 (0.515, 1.527)	0.699	0.5	1.005 (0.906, 1.114)	0.037	0.279	0.01	1	0.27
1-NAP	0.965 (0.735, 1.266)	0.535	0.174	1.001 (0.959, 1.044)	0.038	0.11	0.004	1	0.03
2-NAP	1.179 (0.848, 1.638)	0.535	0.523	1.01 (0.881, 1.158)	0.038	0.711	0.027	1	0.36
2-PHEN	0.999 (0.738, 1.352)	0.535	0.154	1 (0.968, 1.034)	0.038	0.07	0.003	1	0.04
3-PHEN	1.02 (0.744, 1.397)	0.535	0.149	1.001 (0.958, 1.045)	0.038	0.108	0.004	1	0.02
TTMA	0.924 (0.665, 1.284)	0.486	1	0.998 (0.939, 1.06)	0.009	1	0.009	1	0.09
DPHP	1.02 (0.77, 1.351)	0.571	0.126	1.001 (0.966, 1.036)	0.025	0.171	0.004	1	0.04
BDCIPP	0.936 (0.726, 1.206)	0.571	0.103	1 (0.991, 1.009)	0.025	0.069	0.002	1	0
BCIPHIPP	1.074 (0.8, 1.442)	0.571	0.178	1.001 (0.975, 1.027)	0.025	0.222	0.006	1	0.01
EHPHP	1.006 (0.813, 1.245)	0.571	0.105	1 (0.966, 1.034)	0.025	0.245	0.006	1	0.08
BBOEHEP	1.104 (0.848, 1.437)	0.571	0.342	1.001 (0.975, 1.027)	0.025	0.132	0.003	1	0.09
5-OH-EHDPHP	1.013 (0.754, 1.362)	0.571	0.146	1 (0.975, 1.025)	0.025	0.16	0.004	1	0.06
BPA	1.116 (0.832, 1.497)	0.595	0.547	1.011 (0.88, 1.163)	0.034	0.885	0.03	1	0.34
BPF	1.046 (0.816, 1.341)	0.595	0.182	1 (0.978, 1.023)	0.034	0.115	0.004	1	0.08
BPS	0.926 (0.689, 1.244)	0.595	0.271	1 (1, 1)	0.034	0	0	1	0.09
MEP	0.957 (0.776, 1.179)	0.617	0.064	1 (0.983, 1.017)	0.102	0.021	0.002	1	0.01
5cx-MEPP	0.865 (0.59, 1.27)	0.617	0.215	0.989 (0.809, 1.211)	0.102	0.128	0.013	1	0.39
5OH-MEHP	0.962 (0.692, 1.337)	0.617	0.16	1 (0.98, 1.02)	0.102	0.01	0.001	1	0.09
MiBP	1 (0.744, 1.344)	0.617	0.054	0.999 (0.968, 1.032)	0.102	0.037	0.004	1	0.02
MnBP	0.977 (0.763, 1.251)	0.617	0.045	0.997 (0.921, 1.079)	0.102	0.102	0.01	1	0.04

MBzP	1 (0.84, 1.19)	0.617	0.058	0.999 (0.976, 1.024)	0.102	0.044	0.005	1	0
MEHP	0.992 (0.857, 1.147)	0.617	0.043	1 (0.967, 1.034)	0.102	0.082	0.008	1	0.05
OHMEHTP	0.907 (0.726, 1.132)	0.617	0.099	0.995 (0.919, 1.077)	0.102	0.215	0.022	1	0.12
MHNP	0.999 (0.846, 1.18)	0.617	0.043	0.999 (0.96, 1.041)	0.102	0.051	0.005	1	0.03
MCOP	1.005 (0.843, 1.199)	0.617	0.061	1 (0.967, 1.035)	0.102	0.041	0.004	1	0.02
MHNCH	1.153 (0.903, 1.471)	0.617	0.06	1.002 (0.951, 1.056)	0.102	0.089	0.009	1	0.17
OHMIDP	0.992 (0.857, 1.148)	0.617	0.037	1 (0.964, 1.038)	0.102	0.103	0.011	1	0.02
CXMIDP	0.996 (0.89, 1.116)	0.617	0.031	0.997 (0.897, 1.107)	0.102	0.062	0.006	1	0.07
OXOMIDP	1.049 (0.863, 1.273)	0.617	0.029	1.002 (0.951, 1.055)	0.102	0.088	0.009	1	0.06

Abbreviations: BKMR = Bayesian kernel machine regression; BMA = Bayesian model averaging; ENET = elastic net.

Figure S 13. Overall effect of the mixture on lower respiratory infection estimated by BKMR.

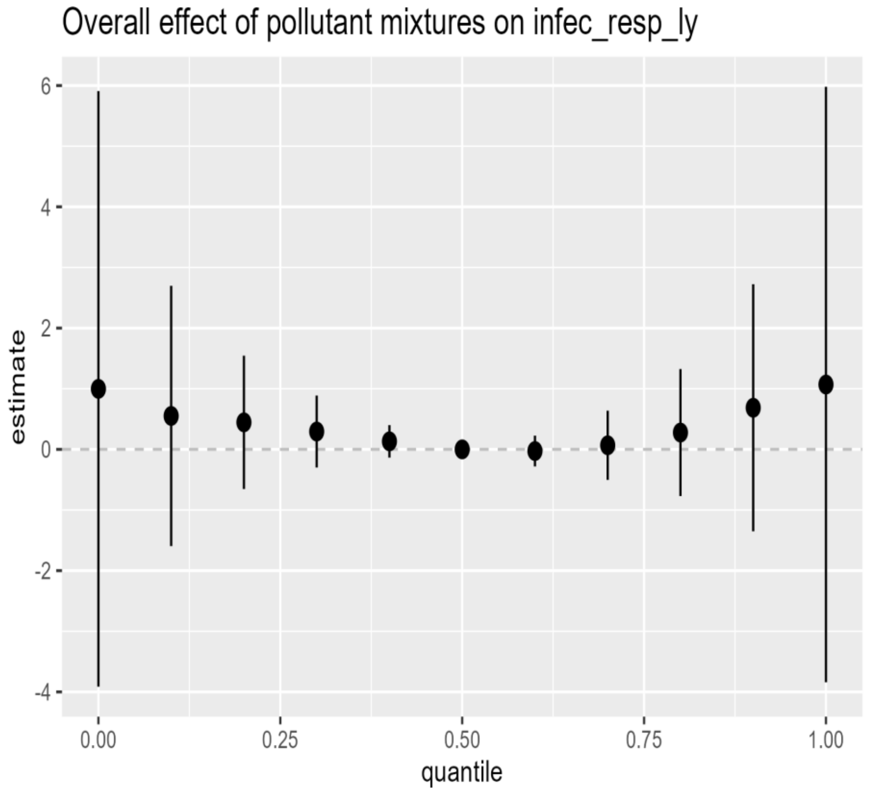

References

Andersen, H. R., L. Dalsager, I. K. Jensen, C. A. G. Timmermann, T. S. Olesen, F. Trecca, F. Nielsen, G. Schoeters, H. B. Kyhl, P. Grandjean, N. Bilenberg, D. Bleses and T. K. Jensen (2021). "Prenatal exposure to pyrethroid and organophosphate insecticides and language development at age 20–36 months among children in the Odense Child Cohort." International Journal of Hygiene and Environmental Health **235**: 113755.

Bastiaensen, M., C. Gys, A. Colles, G. Malarvannan, V. Verheyen, G. Koppen, E. Govarts, L. Bruckers, B. Morrens, C. Franken, E. Den Hond, G. Schoeters and A. Covaci (2021). "Biomarkers of phthalates and alternative plasticizers in the Flemish Environment and Health Study (FLEHS IV): Time trends and exposure assessment." Environmental Pollution **276**: 116724.

Bastiaensen, M., F. Xu, F. Been, N. Van den Eede and A. Covaci (2018). "Simultaneous determination of 14 urinary biomarkers of exposure to organophosphate flame retardants and plasticizers by LC-MS/MS." Analytical and bioanalytical chemistry **410**: 7871-7880.

Covaci, A. and S. Voorspoels (2005). "Optimization of the determination of polybrominated diphenyl ethers in human serum using solid-phase extraction and gas chromatography-electron capture negative ionization mass spectrometry." Journal of Chromatography B **827**(2): 216-223.

De Craemer, S., K. Croes, N. van Larebeke, S. De Henauw, G. Schoeters, E. Govarts, I. Loots, T. Nawrot, V. Nelen, E. Den Hond, L. Bruckers, Y. Gao and W. Baeyens (2017). "Metals, hormones and sexual maturation in Flemish adolescents in three cross-sectional studies (2002–2015)." Environment International **102**: 190-199.

Gys, C., Y. Ait Bamai, A. Araki, M. Bastiaensen, N. Caballero-Casero, R. Kishi and A. Covaci (2020). "Biomonitoring and temporal trends of bisphenols exposure in Japanese school children." Environmental Research **191**: 110172.

Lemke, N., A. Murawski, M. I. H. Schmied-Tobies, E. Rucic, H.-W. Hoppe, A. Conrad and M. Kolossa-Gehring (2021). "Glyphosate and aminomethylphosphonic acid (AMPA) in urine of children and adolescents in Germany – Human biomonitoring results of the German Environmental Survey 2014–2017 (GerES V)." Environment International **156**: 106769.

Nguyen, T. T., T. Higashi, Y. Kambayashi, E. O. Anyenda, Y. Michigami, J. Hara, M. Fujimura, H. Tsujiguchi, M. Kitaoka, H. Asakura, D. Hori, Y. Hibino, T. Konoshita and H. Nakamura (2016). "A Longitudinal Study of Association between Heavy Metals and Itchy Eyes, Coughing in Chronic Cough Patients: Related with Non-Immunoglobulin E Mediated Mechanism." Int J Environ Res Public Health **13**(1).

Schoeters, G., E. Govarts, L. Bruckers, E. Den Hond, V. Nelen, S. De Henauw, I. Sioen, T. S. Nawrot, M. Plusquin, A. Vriens, A. Covaci, I. Loots, B. Morrens, D. Coertjens, N. Van Larebeke, S. De Craemer, K. Croes, N. Lambrechts, A. Colles and W. Baeyens (2017). "Three cycles of human biomonitoring in Flanders − Time trends observed in the Flemish Environment and Health Study." International Journal of Hygiene and Environmental Health **220**(2, Part A): 36-45.

Schoeters, G., V. J. Verheyen, A. Colles, S. Remy, L. R. Martin, E. Govarts, V. Nelen, E. Den Hond, A. De Decker, C. Franken, I. Loots, D. Coertjens, B. Morrens, M. Bastiaensen, C. Gys, G. Malarvannan, A. Covaci, T. Nawrot, S. De Henauw, M. Bellemans, M. Leermakers, N. Van Larebeke, W. Baeyens, G. Jacobs, S. Voorspoels, F. Nielsen and L. Bruckers (2022). "Internal exposure of Flemish teenagers to environmental pollutants: Results of the Flemish Environment and Health Study 2016–2020 (FLEHS IV)." International Journal of Hygiene and Environmental Health **242**: 113972.

Schroijen, C., W. Baeyens, G. Schoeters, E. Den Hond, G. Koppen, L. Bruckers, V. Nelen, E. Van De Mieroop, M. Bilau, A. Covaci, H. Keune, I. Loots, J. Kleinjans, W. Dhooge and N. Van Larebeke (2008). "Internal exposure to pollutants measured in blood and urine of Flemish adolescents in function of area of residence." Chemosphere **71**(7): 1317-1325.

Verheyen, V. J., S. Remy, E. Govarts, A. Colles, L. Rodriguez Martin, G. Koppen, S. Voorspoels, L. Bruckers, E. M. Bijnens, S. Vos, B. Morrens, D. Coertjens, A. De Decker, C. Franken, E. Den Hond, V. Nelen, A. Covaci, I. Loots, S. De Henauw, N. Van Larebeke, C. Teughels, T. S. Nawrot and G. Schoeters (2021). "Urinary Polycyclic Aromatic Hydrocarbon Metabolites Are Associated with Biomarkers of

Chronic Endocrine Stress, Oxidative Stress, and Inflammation in Adolescents: FLEHS-4 (2016-2020)." Toxics **9**(10).
